# Abiotic Stress Phenotypes Are Associated with Conserved Genes Derived from Transposable Elements

**DOI:** 10.3389/fpls.2017.02027

**Published:** 2017-11-28

**Authors:** Zoé Joly-Lopez, Ewa Forczek, Emilio Vello, Douglas R. Hoen, Akiko Tomita, Thomas E. Bureau

**Affiliations:** Department of Biology, McGill University, Montreal, QC, Canada

**Keywords:** transposable elements, phenomics, abiotic stress, exaptation, molecular domestication, high-throughput screen assays, reverse genetics, multiple trait analyses

## Abstract

Plant phenomics offers unique opportunities to accelerate our understanding of gene function and plant response to different environments, and may be particularly useful for studying previously uncharacterized genes. One important type of poorly characterized genes is those derived from transposable elements (TEs), which have departed from a mobility-driven lifestyle to attain new adaptive roles for the host (exapted TEs). We used phenomics approaches, coupled with reverse genetics, to analyze T-DNA insertion mutants of both previously reported and novel protein-coding exapted TEs in the model plant *Arabidopsis thaliana*. We show that mutations in most of these exapted TEs result in phenotypes, particularly when challenged by abiotic stress. We built statistical multi-dimensional phenotypic profiles and compared them to wild-type and known stress responsive mutant lines for each particular stress condition. We found that these exapted TEs may play roles in responses to phosphate limitation, tolerance to high salt concentration, freezing temperatures, and arsenic toxicity. These results not only experimentally validate a large set of putative functional exapted TEs recently discovered through computational analysis, but also uncover additional novel phenotypes for previously well-characterized exapted TEs in *A. thaliana*.

## Introduction

As sessile organisms, plants have complex interactions with their local environment. While a given environment's availability of resources (light, water, nutrients, competition) can affect plant fitness, plants also influence their environment by storing carbon, fixing nitrogen, and producing oxygen. In situations of abiotic stress—defined as environmental conditions that reduce growth and yield below optimum levels—plants respond in a dynamic and complex fashion, involving multiple pathways in various organs, and also involving reversible and irreversible responses (Cramer et al., 2011). Therefore, uncovering phenotypes and making a meaningful connection between the genotype and phenotype presents a challenge, especially for previously uncharacterized genes for which no function or pathway is known.

Various approaches, such as forward genetic screens and yeast one-hybrid assays, have successfully uncovered key genes involved in responses to abiotic stresses (Berthomieu et al., 2003; Koiwa et al., 2006; Assuncao et al., 2010). However, while the introduction of next-generation sequencing technologies has increased genotypic data exponentially for many crop species, the corresponding phenotypic data associated with these genotypes is still lagging (Knecht et al., 2016). This slower progress in developing phenotyping capability may limit our ability to dissect the genetic basis of complex traits such as plant adaptation to a diversity of stresses. Unlike the relatively straightforward case of monogenic traits, traits involving multiple genes and responses to environmental conditions, such as quantitative traits, are typically difficult to decipher (Rahaman et al., 2015). The phenotypic bottleneck is well-described by Furbank and Tester (2011), highlighting the lack of capacity of the plant scientific community to discover the subtle and complex phenotypic effects of genetic modifications. Pereyra-Irujo et al. (2012) also mentioned that phenotyping is an important bottleneck in drought tolerance improvement, and Flood et al. (2016) suggested that the bottleneck in plant sciences has been shifted from genotyping to phenotyping. Phenomics is a rapidly emerging area of science, which aims to characterize phenotypes in a rigorous way and link traits to associated genes and gene variants. Therefore, phenomics technologies are key elements to improving our knowledge of the genotype–phenotype associations of desired traits (Neilson et al., 2015).

A phenomics approach would also be useful to study poorly characterized components of genomes, such as transposable elements (TEs) and TE-related sequences. Transposable elements, also known as transposons, are mobile genetic elements that populate prokaryotic and eukaryotic genomes (Wicker et al., 2007). Long held as parasitic or junk DNA because of their ability to transpose and persist in host genomes without necessarily conferring an apparent selective advantage, recent studies have thoroughly undermined this paradigm and shown that TEs play functional roles in genomes and gene networks (Feschotte, 2008; Lisch, 2013; Joly-Lopez and Bureau, 2014; Wendel et al., 2016). A striking example is the process of TE exaptation (also known as domestication or co-option) where the structural, regulatory, or coding sequences of TEs, previously involved in transposition, confers a novel function with a selective advantage for the host (Feschotte and Pritham, 2007; Sinzelle et al., 2009; Hoen and Bureau, 2012; Joly-Lopez and Bureau, 2014). Fixed exapted TE genes (ETEs) have transitioned from evolving under selfish selection, as in the case of TEs that can persist simply by replicating, to evolving under phenotypic selection, as is the case for host genes that persist only by providing a beneficial phenotype. In plants, most ETEs characterized to date are likely developmentally important transcription factors (Bundock and Hooykaas, 2005; Lin et al., 2007; Ouyang et al., 2011).

Despite their adaptive potential, a systematic search for plant ETEs had been lacking. Indeed, the majority of genes that turned out to be ETEs had been uncovered by forward genetics screens, and their ancestry as TEs discovered only subsequently (e.g., Hudson et al., 2003; Bundock and Hooykaas, 2005; Kapitonov and Jurka, 2005). Recently however, a systematic computational search exploited differences between the genomic signatures of TEs and ETEs to predict a large set of previously unknown ETEs, including 36 in the model plant *Arabidopsis thaliana*—more than double the total number previously discovered (Hoen and Bureau, 2015). These novel ETEs have similar genomic characteristics to known ETEs and the ones derived from DNA transposons represent cases where the entire coding sequence of the transposase has been exapted. These novel ETEs are also conserved between *A. thaliana* and other members of the family Brassicaceae (Haudry et al., 2013; Hoen and Bureau, 2015). Such conservation, coupled with readily available and mature genomic resources for *A. thaliana*, sets the stage to identify promising traits associated with these ETEs that may be useful in agriculturally exploited species of the Brassicaceae or even more distantly related plants.

In this study we aimed to uncover, for the ETEs in *A. thaliana*, phenotypic profiles using a high-throughput phenomics platform (http://mp3.biol.mcgill.ca) under four abiotic stress conditions, namely high salt, phosphate limitation, freezing temperature, and arsenic toxicity. These were selected because of their negative impact on agricultural productivity: (1) high salinity is one of the most severe environmental factors eroding the productivity of crops, with at least 30% of the world's irrigated areas estimated to be salt-affected (Demiral et al., 2011; Vijayvargiya and Kumar, 2011); (2) inorganic phosphate (Pi) is often the limiting factor for plant growth and development, and is mostly provided via the intensive use of costly fertilizers (Cordell et al., 2009); (3) damage caused by freezing temperatures can lead to reduced crop yield and quality (Moellering et al., 2010); (4) the erosion of naturally arsenic-rich soil, combined with growing industrial processes, are primary sources of arsenic pollution in water used for agriculture (Rodriguez-Lado et al., 2013).

## Materials and methods

### Exapted TE and reference genes

The exapted TEs (ETEs) analyzed in this study were selected from the study conducted by Hoen and Bureau (2015). Reference genes used in the assays were pre-selected from the literature as already having a phenotype under similar or identical phenotyping conditions. The availability of T-DNA insertion mutant alleles for each ETE and reference gene was identified through The Arabidopsis Information Resource (TAIR; Stanford University, Stanford, CA USA) genome browser (version 10) (Figure S1). Alleles were selected according to the likelihood that they would disrupt the ETE or reference gene coding potential (Table S1). If available or applicable, ETEs, or reference genes with previously described T-DNA insertion mutants were used.

### Plant seeds

All seeds (ETE mutants and reference lines) were obtained directly from the Arabidopsis Biological Resource Center (Ohio State University, Columbus, OH USA) or through TAIR portal. Wild-type *A. thaliana* ecotype Col-0 seeds were originally obtained from Lehle Seeds (Round Rock, TX USA). Bulked seeds were dried for 10 days after harvest and stored at 4°C. Seeds were vapor-sterilized in a desiccator jar as describe in Bent (2000).

### Oligonucleotide primers

Oligonucleotide primers were designed using the SIGnAL T-DNA primer design tool (http://signal.salk.edu/tdnaprimers.2.html) using default parameters, except Ext5 = 400 was used. Primers were ordered *en masse* (Integrated DNA Technologies, IA USA) at a 25-nmole scale in a 96-well format. Three primers were used in each PCR with two target-specific T-DNA flanking primers and a primer that anneals to the left-border of the T-DNA (Table S1).

### Genotyping

Experimentally validated homozygous lines were used in the study. Seeds were either grown on square Petri plates containing 40 ml of ½ strength MS (Murashige and Skoog) media, 2.5 mM MES, 1% sucrose, and 0.8% agar or were sown individually into cells (3 cm^2^) of a plug tray (8 × 12) containing soil (2 parts Pro-Mix: 1 part vermiculite: 1 part perlite; Premier Tech Horticulture, QC Canada) pre-moistened with a fertilizer solution (20N: 20P: 20K). After stratification for 3 days in the dark at 4°C, plates or trays were transferred to a growth chamber (Conviron, MB Canada; model A1000) with constant 16 h light (120 μmole quanta/m^2^/s): 8 h dark at 22°C and 70% humidity. A leaf from 3-week-old seedlings (diameter <5 mm) was harvested and genomic DNA extracted using a modified NaOH-based protocol (Klimyuk et al., 1993).

PCR was performed in a 25 μl total volume consisting of 1 μl of genomic DNA template (40–110 ng/μl), 1X PCR buffer, 2 mM MgCl_2_, 0.1 mM dNTPs, 0.5 U of native *taq* polymerase (Invitrogen, CA USA), and 0.4 μM of each primer (see above). Reactions were performed on 0.2 ml 96-well thermal cyclers (Applied Biosystems, ON Canada; model Veriti) using the program of 94°C for 1 min, 40 cycles: 94°C for 1 min, 60°C for 1 min, and 72°C for 2 min, 72°C final extension for 10 min, and a 4°C hold. Reaction products were run on 1.2% agarose gels and visualized using a gel documentation station (Bio-Rad, CA USA; model EZ System). Homozygous lines were identified by comparing the observed vs. predicted band sizes. Reactions with wild-type and no DNA were used as controls. Lines that did not yield clear homozygous individual using three primers (see above) were re-genotyped using two separate reactions, one with only the gene specific primer pair and the other the T-DNA and one gene-specific primer pair. Lines that still did not yield any clear homozygous individuals were replanted and re-genotyped. Validated unambiguous homozygous seedlings were repotted or transferred into 4 cm square pots, staked, and allowed to grow to seed set.

### Plant growth facilities

Plants were grown at the Phytotron located at McGill University, Department of Biology (Montreal, Quebec, Canada; http://www.biology.mcgill.ca/Phytotron).

### Image acquisition platform

For the salt, arsenic and phosphate assays, image acquisition and analyses were performed at the McGill Plant Phenomics Platform (MP3; Montreal, Quebec, Canada; http://mp3.biol.mcgill.ca) using a customized version of the LemnaTec scanalyzer High-Throughput Screening (MP3-HTS) (LemnaTec GmbH, Wuerselen, Germany), where up to 60 plates per run can be analyzed. In this study, images were only taken with the visible light camera piA2400-17gc (VIS) and the fluorescent light camera scA1400-17gc (FLUO). For the freezing assay, image acquisition was done using a Nikon D3100 camera with AF-Micro Nikkor 60 mm 1:2.8G ED mounted on a static support. Image files were stored on a Dell R910 server (MP3 server) with 256 GB of RAM and two MD1200 storage devices (72 TB).

### Image and statistical software

A custom image analysis algorithm was developed using java 1.7.0-45 (http://www.java.com) and ImageJ library v1.48 (http://imagej.nih.gov/ij/) (Schneider et al., 2012) for salt, arsenic, phosphate and freezing. The statistical analysis script was written in R v3.0.2 (Team, 2013) (http://www.r-project.org/) for all the assays. A modified version of the R script published in Camargo et al. (2014) was used to generate the figures **Figure 2**, Figures S2–S5. PostgreSQL v 9.3.1.was used to build the database. The analyses were implemented on the MP3 server. *P*-values were adjusted for multiple comparisons using the method of Benjamini & Hochberg of false discovery rate control (Team, 2013). Only lines having an FDR-adjusted *P*-value lower than 0.05 were considered to have a stress-related phenotype.

### Salt stress assay

The salt assay was adapted from the methods described by Zhang et al. (2011). Three reference salt sensitive genes were used in the assays: *SOS2* (At5g35410; SALK_016683) and *SOS3* (At5g24270; SALK_137171, CS859749) as reported by Shi et al. (2000), and *DDF1* (At1g12610; SALK_127759, SALK_114390) as described by Magome et al. (2008). Seeds (ETE mutant lines, wild-type, and reference lines) were sown on square Petri plates with grid (Simport, Canada) containing MS medium buffered at pH 5.6 with 1% v/w sucrose, 2.5 mM 2-(N-morpholino) ethane sulfonic acid (MES), and 0.8% agar. After 3 days stratification at 4°C in darkness, the plates were placed in a growth chamber (Conviron model A1000, Canada) programmed for a 16 h light (120 μmole quanta/m^2^/s), 8 h dark photocycle (22°C, 70% humidity). To induce salt stress, 4-day old seedlings were transferred to MS medium supplemented with 150 mM NaCl (Zhang et al., 2011), a concentration corresponding approximately to the LD50 for wild-type. Assays were performed using 36 seeds per plate for each of the three independent biological replicates. For the standard growth condition, lines were grown for 12 days on MS medium without NaCl.

Image acquisition was performed using the VIS and FLUO cameras. Visible light RGB images were taken for each Petri dish using a “one frame one plate” setup with top light illumination. Pixels having saturation (S) of the HSB color space higher than 49 were retained (74 for the standard growth condition). A grayscale transformation was then done keeping only the pixels with values between 0 and 140 and from 150 to 255. The RGB-values of the selected pixels were tagged as foreground to identify the objects using an adapted version of the “combined contour tracing and region labeling” algorithm proposed by Burger and Burge (2008). Objects having an area >40 px and an Euclidean distance lower than 130 to the center of the grid cell were selected. Only the largest object in each grid cell was kept. As a result, each seedling was represented by one object. The hue channel of the HSB color space was divided in 16 categories where each object pixel was classified (Berger et al., 2012). Fluorescent light images were acquired using the same frame configuration as visible light. Intensity images were converted to HSB color space. Pixels with S higher than 20 were tagged as foreground. After identifying the objects using the Burger and Burge adapted algorithm, objects having an area >20 were selected (Burger and Burge, 2008). Only the largest object within each grid cell was kept as representation of the seedling. The same color classification was done as above. An Euclidean distance matrix of the objects was calculated with the color classes of the visible light camera joined to the classes of the fluorescent camera using as input for a hierarchical cluster analysis. The resulting hierarchical cluster tree was divided into two groups. The number of seedlings belonging to each group was counted and tabulated per line. The most representative lines of each group were analyzed to determine the characteristic of each group. To detect the candidate lines, a Fisher's exact test was used on 2 × 2 contingency tables built for each of the lines having the target line and the wild-type as columns and the clustered groups as rows.

### Phosphate limitation stress assay

The phosphate assay was based on the methods described by Misson et al. (2004). Three reference phosphate responsive genes were used in the assays: *LPR1* (At1g23010; *lpr1-2*, SALK_102704) and *LPR2* (At1g71040; SALK_138670) as reported by Svistoonoff et al. (2007), and *IPK1* (At5g42810; SALK_065337) as first characterized by Stevenson-Paulik et al. (2005). Seeds sown on square Petri plates (Simport, Canada) containing 1/10 strength MS medium (0.15 mM MgSO_4_, 2.1 mM NH_4_NO_3_, 1.9 mM KNO_3_, 0.5 mM NaH_2_PO_4_, 0.3 mM CaCl_2_, 0.5 μM KI, 10 μM Fe, 10 μM H_3_BO_3_, 10 μM MnSO_4_, 3 μM ZnSO_4_, 0.01 μM CuSO_4_, 0.01 μM CoCl_2_, 0.1 μM Na_2_MoO_4_, 2 μM EDTA, 0.03 μM thiamine, 0.24 μM pyridoxine, 0.4 μM nicotinic acid, 55 μM inositol, and 14 mM MES; pH 5.8), 0.5% sucrose, and 0.8% agar (Murashige and Skoog, 1962). In the Pi limiting medium, a concentration of 5 μM NaH_2_PO_4_ was added instead of 0.5 mM NaCl was used to replace the equivalent amount of sodium provided by NaH_2_PO_4_. After 3 days at 4°C in the dark, plates were placed in a vertical position in a growth chamber (Conviron model A1000, Canada) programmed for a 14 h light (22°C), 10 h (18°C) dark photocycle (70% humidity, 120 μmole quanta/m^2^/s) and grown for 9 days, after which images were acquired. Six seeds per plate were used for each of two independent biological replicates, for a total of 12 plants per line. The standard growth condition consists of growing seedlings as described above on the 1/10 strength MS medium with 5 μM NaH_2_PO_4_.

Image acquisition was performed using the VIS camera. A setting of one frame for each Petri dish and bottom light illumination was used. Images were transferred to the MP3 server for further analysis. Every image was converted to a grayscale image and the mean auto local threshold algorithm was applied to make a draft separation of the background. The grid embossed on the Petri dish was digitally removed from the image to not confound downstream analysis. Horizontal lines >50 px and vertical lines >240 px were removed. After eroding 1 px size and removing top and bottom border lines from 50 to 260 and 1,698 to 1,798 Y coordinates, the resultant image was re-converted to RGB using the original image as template. A list of objects was created using the same algorithm as used for the salt assay and only the objects having an area >109 px, a circularity lower than 0.70, and an Euclidean distance lower than 145 to the center of the grid cell were retained, representing the root portion of the plant. For the shoot part of the plant, only the pixels having the bright (B) of the HSB color space higher than 23 and lower than 138 were kept from the original images. A list of object was created and only the objects having an area >10 px, a circularity rate >0.099 and an Euclidean distance to the center of the grid cell lower than 130 were considered to belong to the shoot part of the seedlings. The shoot and root objects of each plant were merged into one object using the location of the shoot as a start point and the vertical lines of the grid on the plate as reference. The results provided a list of objects where each of them represents one plant. A one-way ANOVA on the major axis calculated from the digital plant was used between each line and wild-type. The major axis is defined as the axis where a physical body requires less effort to rotate. It extends from the centroid to the widest part of the object (Burger and Burge, 2008).

### Freezing stress assay

The freezing stress assay was performed as previously described (Xin and Browse, 1998). The protocol without acclimation and with a freezing temperature of −8°C proved to be a good trade-off both in terms of resolution (discriminating survivability) and stress effect (number of affected seedlings per plate) for high throughput. Two lines for the freezing sensitive reference gene *SFR2* (At3g06510; SALK 106253C, and SALK 000226) were used in the assays, as described by Moellering et al. (2010). Seeds were sown on 0.9% agar round plates (100 × 15 mm) with Gamborg's B5 basal salt medium (Gamborg et al., 1968) (PhytoTechnology Laboratories, USA) and 1.5% sucrose. After 2 days of stratification at 4°C in the dark, plates were transferred to a growth chamber (Conviron model A1000, USA) set at 22°C and constant 24 h light (120 μmole quanta/m^2^/s and 70% humidity) for 10 days. Then, plates were sat on ice in a freezing chamber (Conviron model PGW36, Canada) for 16 h at −1°C in the dark. Ice chips (Hoshizaki ice machine, model F-450 BAB, USA) were then sprinkled directly on seedlings, after which the temperature of the freezing chamber was decreased at a rate of 1°C per hour until it reached −8°C and then held for a duration of 2 h. After the freezing treatment, plates were thawed at 4°C in the dark for 12 h. Then, seedlings were recovered at 22°C under constant 24 h light for 48 h. Afterwards, image acquisition was done as described below. Three independent biological replicates were performed using 60 seeds per plate each time. For the standard growth condition, lines were grown on Gamborg's B5 basal salt medium for 10 days as described above.

For the image analysis method, pixels with blue (B) of RGB color space lower than 127 and (red(R) − blue(B))/(red(R) + blue(B)) (Kawashima and Nakatani, 1998) >0.20 were considered as foreground. The identification of the objects from the foreground was performed using the same algorithm as in the salt assay. Only the objects having an area between 10,000 and 40,000 px were considered as representations of seedlings. The hue channel of the HSB color space was divided in 32 categories where each object pixel was classified (Berger et al., 2012) and the categories were re-converted to RGB. The objects were then divided into two groups according to the amount of leaf damage represented by the yellowish that was calculated from the number of pixels in the color classes 4,12,20,28,36 (hue channel of the HSB color spectrum) over the total number of pixels. The number of seedlings belonging to each group (less or more of 50% of damage) were counted and tabulated per line. The statistical analysis is the same as in the salt assay.

### Arsenic stress assay

The arsenic stress assay was based on a protocol described by Lee et al. (2003). The arsenic resistant gene *ARS5* (At5g42790, *ars5-2*, CS440215) was used as a reference gene, as described by Sung et al. (2009). Seeds were sown on minimal medium containing 2.5 mM H_3_PO_4_, 5 mM KNO_3_, 2 mM MgSO_4_, 1 mM Ca(NO_3_)_2_, 1 mM MES, 0.5% sucrose, and 0.8% agar (pH 5.7). Macronutrients were replaced with 750 μM potassium arsenate (Sigma-Aldrich Life Science. USA), a concentration determined to be the mean lethal dose (LD50) for wild-type seedlings. Plates were stratified for 2 days at 4°C in the dark and then transferred to a growth chamber (Conviron model A1000, Canada) set at 22°C, constant light (120 μmole quanta/m^2^/s) and 70% humidity for 5 days after which images were acquired. Three independent biological replicates were performed, where 36 seeds per line were used in each replicate. For the standard growth condition, lines were grown for 5 days on minimal medium without potassium arsenate.

Image acquisition was performed using the VIS and FLUO cameras. The Petri dish covers were not removed during image acquisition to avoid contamination. A setting of one frame one plate for each Petri dish and bottom light illumination was used for the VIS camera. Pixels having red (R) lower than 254, green (G) lower than 246, and blue (B) lower than 254 of the RGB color space were retained. Identification of the objects from the foreground was done using the same algorithm as in the salt assay (see above). Objects having an area >10 px, a circularity ratio >0.099 and an Euclidean distance lower than 130 to the center of the grid cell were selected. Only the largest object within each grid cell was kept. As a result, each seedling was represented by one object. The fluorescent light images were acquired using the same frame configuration as the visible light images. The intensity images were converted to HSB color space. Pixels with S higher than 50 were tagged as foreground. After identifying the objects using the Burger and Burge adapted algorithm (Burger and Burge, 2008), objects having an area >20 were selected. Only the largest object within each grid cell was kept as representation of the seedling. The color classification, clustering, statistical test and filtering used were as described in the salt assay.

### Repository of phenotypes

The phenotypic results of the mutant lines for each assay are available on our phenomics database at http://mp3.biol.mcgill.ca/dteassays.

## Results and discussion

### Reverse genetics approach

As compelling as the features gleaned by computational methods are in arguing a functional contribution for ETEs, it is nevertheless prudent to confirm genotype-phenotype relationships by experimentation. We exploited the availability of T-DNA large insertion mutant populations for *A. thaliana* (Alonso et al., 2003) to establish one to four homozygous putative knockout lines per candidate ETE, which we used for phenotyping in trait assays (Figure S1, Table S1). The confirmation of the homozygosity of the mutant alleles was achieved using PCR-based genotyping (Table S1). In addition to wild-type *A. thaliana* (Col-0) and T-DNA mutant lines from reference genes known to affect each trait, we assayed 103 independent T-DNA mutant lines representing 50 of the 67 novel and previously characterized ETEs (Table S2). The remaining novel ETEs could not be assayed due to the limited availability or absence of available T-DNA mutant lines within regions likely to significantly alter the function of the gene. Among these 50 genes, about half (24) were known ETEs with the remainder (26) being novel (Hoen and Bureau, 2015).

### Phenotypic data analysis

**Figure 1:**
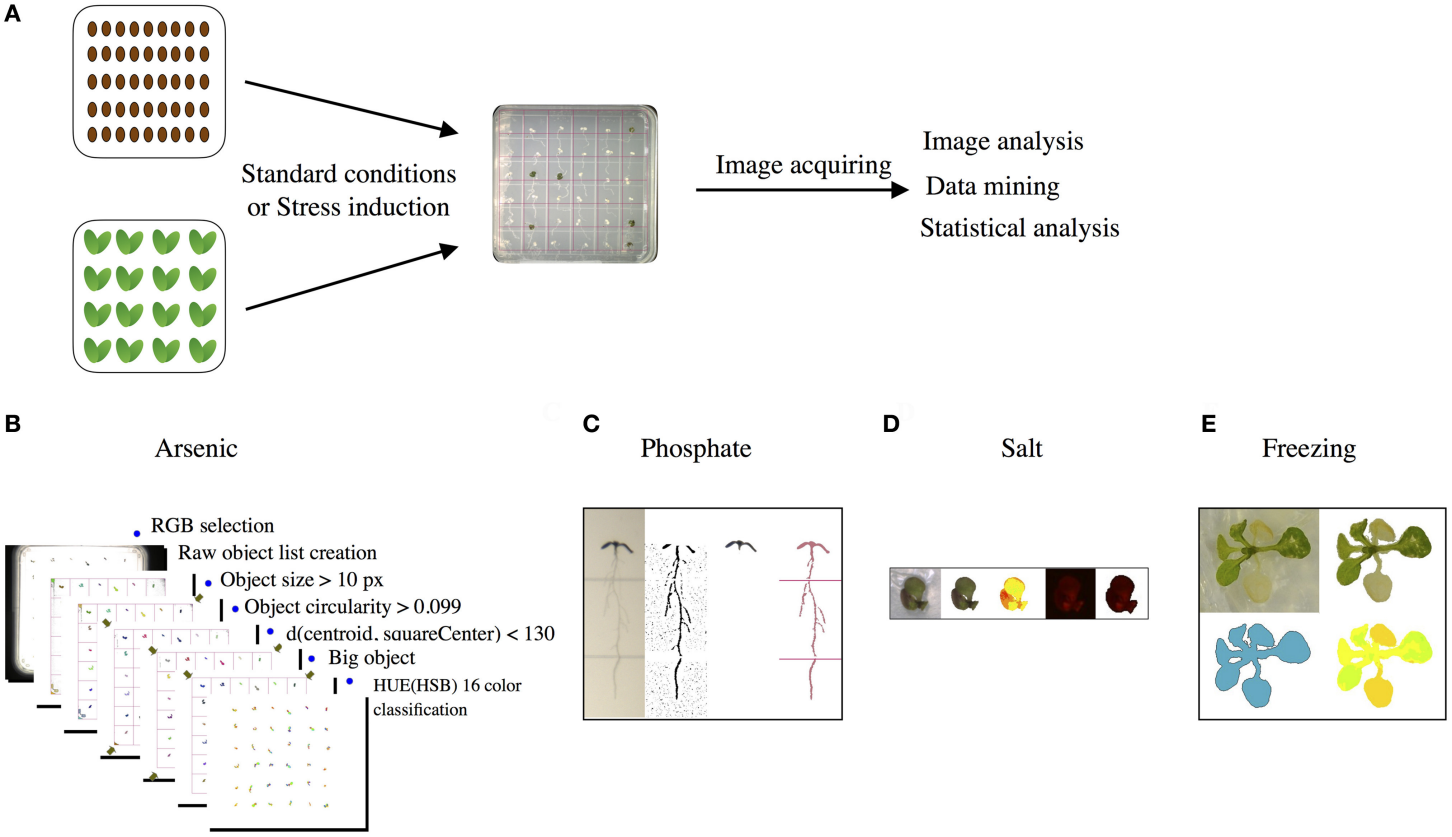
Example of image processing analyses for plants grown under standard growth conditions or stress conditions. **(A)** Overview of the phenotypic analysis for all the assays. The brown ovals represent seeds and the green hearts represent seedlings. **(B)** Image analysis pipeline detail for arsenic stress including plant segmentation, features calculation and color classification. **(C)** Main steps of the phosphate image analysis process. **(D)** Color classification steps from visible (third first squares) and fluorescent (last two squares) light in salt stress. **(E)** Yellowish quantification process from images in freezing condition.

We looked at the response of the ETE T-DNA mutant lines grown under standard laboratory growth conditions, and then subjected to four abiotic stress conditions, namely phosphate limitation, freezing temperature, and exposure to semi-lethal concentrations of either arsenic or salt. To make this study more amenable to high-throughput, we designed plated-based assays for each condition (standard and abiotic stress) to assess the *A. thaliana* ETE T-DNA mutant lines for phenotypes at the seedling stage, and acquired images of each plate using an automated phenomics platform. We also developed a custom phenotypic data analysis pipeline using the acquired images as input for each assay (Figure 1) (see section Materials and Methods). The use of a phenomics platform increased the reproducibility of the measurements, such that different measurements like leaf size, color, and root length, were more comparable (Fahlgren et al., 2015; Vello et al., 2015; Flood et al., 2016) The pipeline of a phenomics analysis requires three steps, namely: image analysis, data mining, and statistical analysis (Figure 1A). Image analysis has two major components: first, segmentation, where the image is partitioned into sets of pixels to select those representing the plant (i.e., the digital plant), and second, the calculation of the morpho-colorimetric features (e.g., projected leaf area), based on the digital plant. We developed specialized segmentation algorithms for each experimental condition (e.g., salinity, freezing), accounting for differences in medium composition, experimental requirements, and growth patterns in each protocol. For example, while for most protocols the plastic plate lids were removed to capture plate images, for the arsenic experiment they were not removed because of safety reasons and, as a result, back light rather than a top light was used to avoid reflection off the lids. Another example is the type of information of interest for each condition: for the phosphate limitation assay the pixels representing the roots were our main interest, whereas for the salinity assay it was the shoots (see section Materials and Methods).

We then used data mining techniques to identify proxies for the main phenotypic traits that were previously identified in studies of phenotypic responses in *A. thaliana* under the different stress conditions used in this study (Xin and Browse, 1998; Lee et al., 2003; Misson et al., 2004; Verslues et al., 2006; Zhang et al., 2011). Data mining techniques do not require human intervention to evaluate the phenotype and are amenable to high-throughput protocols. For the arsenic and salt assays, a cluster of color classification has been shown to be optimal to assess germination and survivability, respectively (Berger et al., 2012; Vello et al., 2015). In the former case, a seed is represented by a brown color whereas a seedling's predominant color is green. In the latter case, the seedlings that are alive retain some green areas (Figure 1). The data mining results for the arsenic and salt assays were validated by a blind manual scoring of 216 seedlings of random T-DNA lines, and showed over 90% agreement (Figure S2). For the freezing assay, a color classification of yellowish tones was used as a proxy for leaf damage. Finally, no color classification was required for the phosphate limitation assay since the root elongation was the main phenotypic trait (Figure 1). The statistical approaches that were used to analyze the data mining results are described in the following section.

#### Analysis of ETE T-DNA mutant lines using a main phenotypic trait

**Figure 2:**
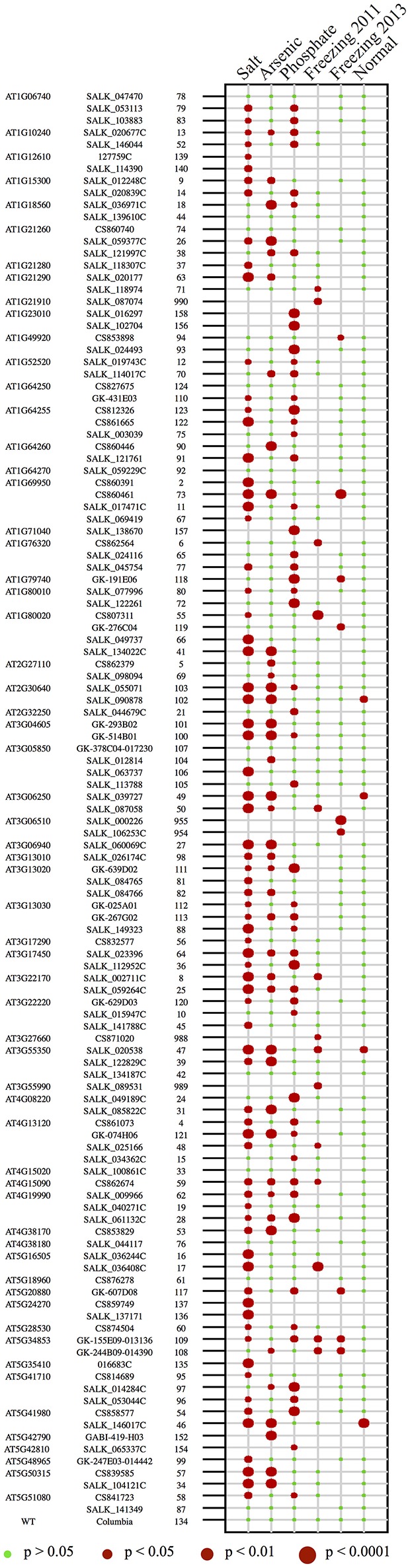
*P*-value for all 103 lines, including reference genes, for the five growth conditions. Summary of the FDR-corrected *P*-value for each of the abiotic stress assays, in addition to standard growth conditions. Each number corresponds to a T-DNA mutant line, as indicated in the left column. The lines are also organized by exapted TE genes (top left column gene IDs). The size of the dots corresponds to a different *P*-value threshold. The absence of a red dot indicates that the line was found to be non-significant after our analysis. The results for the freezing assay were separated by the year during which the lines were tested.

To detect potential phenotypic differences between wild-type and the T-DNA mutant lines, we first looked at one main phenotypic trait per assay (e.g., germination, survival), as described above (Figure 1; see section Materials and Methods). With respect to the analysis, we assessed the phenotypic differences between all 103 T-DNA mutant lines and wild-type by using Fisher's exact tests for the salt, arsenic, and freezing assays. For the phosphate limitation assay, an ANOVA test was chosen since the root length represents a quantitative measure compared to the other parameters. The *P*-values were adjusted for multiple comparisons testing by applying Benjamini & Hochberg's false discovery rate control method (see section Materials and Methods). Our results reveal that 93 of the 103 T-DNA mutant lines displayed a significant phenotype under at least one condition (Figure 2). In addition, the T-DNA lines corresponding to the reference genes that were selected for their known mutant phenotypic effect under a specific abiotic stress also showed a significant phenotype, serving as positive controls (Figure 2).

#### Analysis of ETE T-DNA mutant lines using multiple morpho-colorimetric phenotypic traits

To further characterize the ETE T-DNA mutant lines, we used another approach that includes multiple phenotypic traits and takes full advantage of the rich image data produced by the phenomics platform. In order to simultaneously examine multiple phenotypic traits, we looked at differences between wild-type and our T-DNA mutant lines using eight morpho-colorimetric features (see Table 1): area, perimeter, circularity, compactness, major axis, minor axis, eccentricity, and gray intensity peak (hisgreypeak). For each condition, all the plants screened for a given T-DNA line were given a morpho-colorimetric profile based on these eight features. The number of plants (*n)* varied for each assay based on the experimental setup (e.g., *n* = 12 in phosphate-limitation; *n* = 108 in salt). The digital plants were then clustered first based on profile similarity and then using a hierarchical clustering, to produce a dendrogram and heatmap based on feature similarity.

**Table 1:** Definitions of the morpho-colorimetric features.

**Features**	**Definitions**	**References**
Area	Number of pixel of the digital plant	
Perimeter	Length of the outer contour of the digital plant	Burger and Burge, 2008
Circularity	Ratio between the circumference square and the area	Camargo et al., 2014
Compactness	Ratio between the area and the perimeter	Burger and Burge, 2008
Major axis	Axis where a physical body requires less effort to rotate. It extends from the centroid (center of gravity) to the widest part of the object, in this case the digital plant	Burger and Burge, 2008
Minor axis	Axis perpendicular bisector to the main axis	
Eccentricity	Ratio between the major axis and the minor axis of the digital plant. The minor axis extends from the centroid to the narrowest part perpendicular to the major axis	Burger and Burge, 2008
Gray intensity peak (hisgreypeak)	Intensity value having the bigger frequency from the pixels of the digital plant. In other words, it is the higher peak of the intensity value histogram	

**Figure 3:**
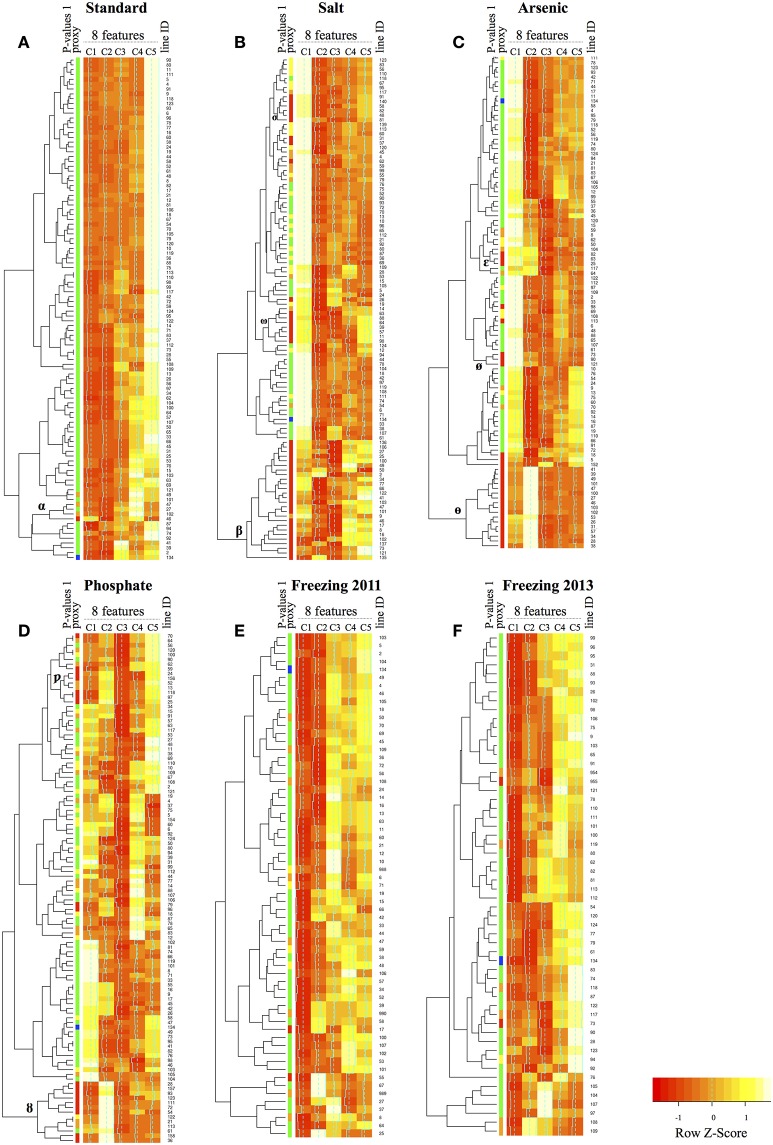
Heatmaps for the eight morpho-colorimetric features. Heatmaps corresponding to the standard growth **(A)** conditions, as well as the four abiotic stress conditions **(B–F)**. The freezing assay was conducted over two years and thus has two heatmaps **(E,F)**. The eight morpho-colorimetric features used for this analysis are listed here and defined in Table 1: area, perimeter, circularity, compactness, major axis, minor axis, eccentricity, and gray intensity peak (hisgreypeak). For each heatmap, the clusters within each line were arbitrarily divided into five groups (labeled C1-C5) to facilitate visualization. To compare the cluster analysis with the “standard definition of trait” results, we added a column showing the *P*-values for each line, and wild-type (line 134, in blue). For each line, the density of plants belonging to each cluster is color coded, ranging from pale yellow for low density, to red for high density.

Figure 3 shows the heatmaps for the tested conditions. For each heatmap, the clusters within each line were arbitrarily divided into five groups (labeled C1–C5) to facilitate the analysis. To compare the cluster analysis with the results of our analysis based on one main pre-defined phenotypic trait, we added a column showing the *P*-values for each mutant line and wild-type (see Figure 3 legend). Under standard growth conditions, a handful of mutant lines with a significant *P*-value were closely positioned on the dendrogram (group labeled “α”) and their clustering showed a noticeably different profile from wild-type, especially in the case of line 46 (blue, line 134, Figure 3).

For the heatmaps representing lines under stress conditions, we also detect groups of lines having profiles that differ from wild-type, in addition to having low *P*-values under the “standard definition of trait” analysis (Figure 3). Clusters with different profiles and low *P*-values did not group together in the dendrogram but rather formed asymmetric groups along the dendrogram (see labeled groups in Figure 3; e.g., labeled “β,” “ω,” and “σ” in salt).

Within these groups of clusters, T-DNA mutant lines corresponding to the same gene almost always clustered together, as well as genes from the same or related gene families (Table S3). For example, in the heatmap for the salt assay, the group of clusters “β” encompasses all the T-DNA lines corresponding to the reference genes from the SALT OVERLY SENSITIVE (*SOS*) pathway included in our assay, whereas the T-DNA lines of the other reference gene *DWARF AND DELAYED FLOWERING 1* (*DDF1*), which is a transcription factor involved in a different pathway as of *SOS* genes, has a profile more similar to that of the lines in the group of clusters “σ” (Figure 3B). Also included in the group of clusters “β” are two lines for genes of the *MUSTANG-A (MUGA)* and *MUSTANG-B (MUGB)* families, the *FAR-RED ELONGATED HYPOCOTYLS 3 (FHY3)* family, as well as an overrepresentation of ETEs derived from the hAT transposon superfamily. Whereas, in the groups of clusters “ω” and “σ” we find T-DNA mutant lines for ETEs derived from class I retrotransposons (Tables S2, S3). A similar trend was also observed for the arsenic heatmap, where T-DNA mutant lines for the same gene were grouped together (Figure 3; “θ”). Moreover, T-DNA mutant lines that displayed significant phenotypes under both salt and arsenic stresses also were found in the same group (Table S3). We did however find five instances in the salt heatmap in which two T-DNA mutant lines for the same ETE were found in different groups of clusters from the large group “β” (lines 11, 14, 57, 39 in ω; 48 in “σ”) (Figure 3, Table S3).

Overall, we found that the multiple parameter analysis can detect phenotypes and clustering supports the classical trait analysis, as shown by differences between mutant line clusters and wild-type.

### Evidence of genotype-phenotype causality

In addition to the 103 ETE T-DNA mutant lines, we also included “reference lines” corresponding to T-DNA mutant lines of well-characterized genes associated with one of the four stresses (Table S2). In total, more than 36,500 seedlings, representing 119 lines, were screened and analyzed (Table S2).

**Figure 4:**
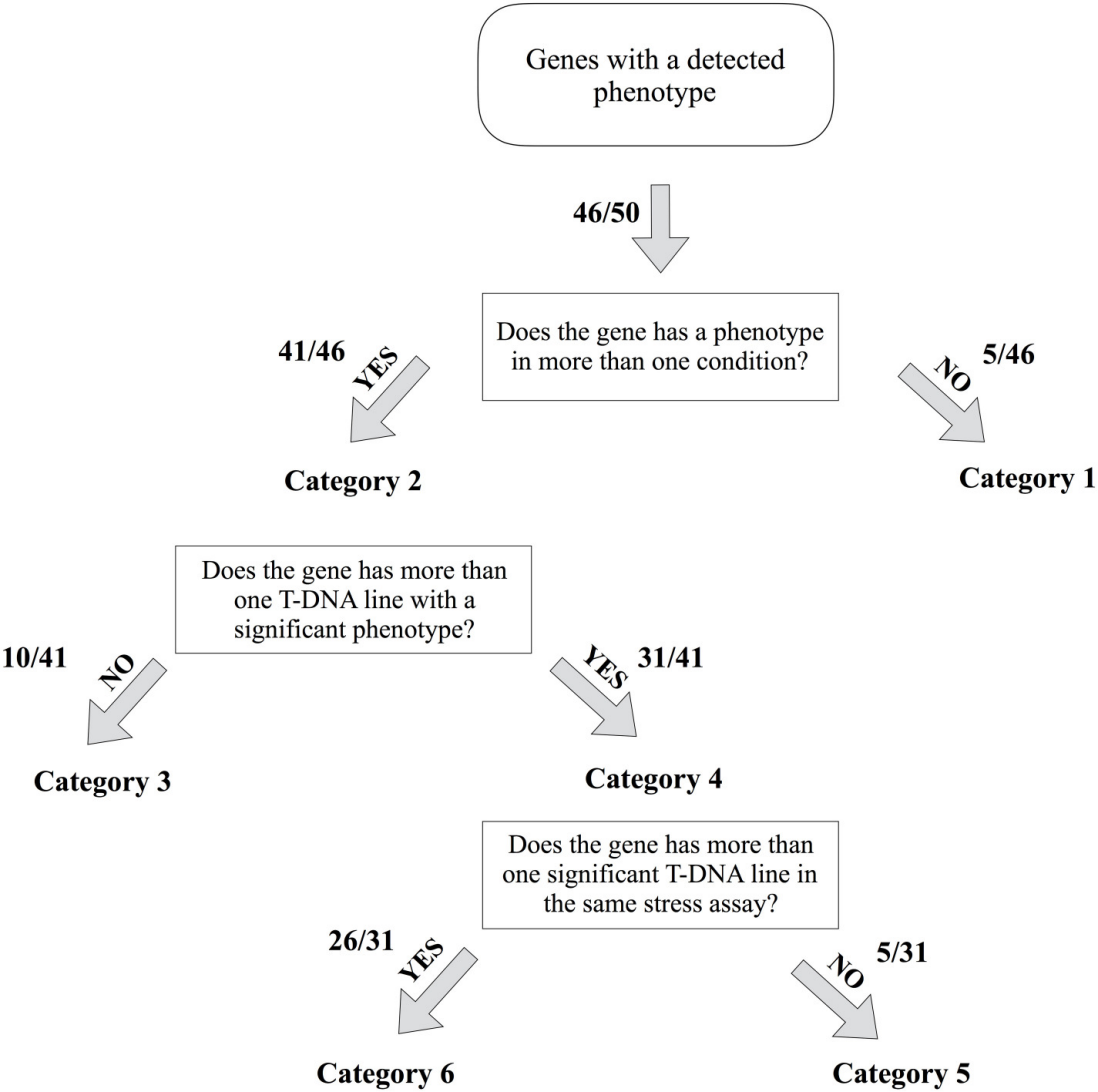
Flowchart diagram for the mutant line categories. Flowchart to categorize the different exapted TE genes for which at least one T-DNA allele displayed a phenotype. Exapted TE genes are placed in six distinct categories based on the answer “yes/no” to three questions. The number of genes belonging to each category is indicated.

Of the 50 ETEs included in our study, 46 had at least 1 T-DNA mutant line with a significant phenotype under one of the four stress conditions using the main phenotypic trait analysis. To guide our analysis of these TE-derived genes, we created a flowchart to group ETEs into categories (Figure 4). For example, ETEs for which one or more T-DNA mutant lines displayed a phenotype in only one growth condition were labeled as “Category 1,” whereas ETEs having one or more T-DNA mutant lines displaying phenotypes in more than one condition were labeled as “Category 2.” To strengthen the evidence that the phenotypes we detected for the T-DNA mutant lines were linked to the mutated ETEs, we labeled as “Category 3” the ETEs for which only one T-DNA mutant allele was available, or where a mutant allele failed to show a phenotype due to a suboptimal location of the gene-specific T-DNA insertion. ETEs for which two or more T-DNA mutant alleles were available and found significant under a given condition were labeled as “Category 4” (Figure 4). Finally, to single out ETEs that had a detected mutant phenotype principally caused by the exposure to abiotic stresses, we identified ETEs for which more than one T-DNA mutant line was found significant in the same stress assay (if not, counted in “Category 5”). This filtering approach led to a subset of 26 ETEs (“Category 6”) (Figure 4). This stringent subset represents experimentally well-supported ETEs derived from different TE superfamilies, almost half of which (12/26) are previously reported ETEs, while the remainder (14/26) are novel (Hoen and Bureau, 2015).

We found four T-DNA mutant lines that differed significantly from wild-type under standard growth conditions, which correspond to four different ETEs including the known ETEs *FRS7* (line 49; *P* = 0.006) and *MUG2* (line 102; *P* = 0.005) (Table S2; Figure 3). Nonetheless, these two genes still figure in the count number of “Category 6” for ETEs responding to abiotic stress because they had two T-DNA mutant lines showing a strong phenotype under one or more stresses, with *P*-values differing by an order of magnitude of 3 (*FRS7*) and up to 19 (*MUG2*) under a given stress compared to standard growth conditions (Table S2). Also, we previously showed, using the same T-DNA mutant lines as in this study, that *MUG1* and *MUG2* single mutants show subtle but significant phenotypes under standard growth conditions [37]. However, the phenotypes were detected at a later developmental stage than in this study, and therefore could explain why mutant lines for *MUG1* did not appear to be significantly different from wild-type under standard growth conditions (Figure 3).

### Exapted TEs with responses under abiotic stress assays

We were interested in singling out ETEs that appeared to have a strong and more specific response to abiotic stress. We considered a response to be strong and specific if a gene had: (1) at least two T-DNA lines found significant in the same stress assay, and (2) an FDR corrected *P*-value lower than 0.01 (see section Materials and Methods). Applying these filters resulted in a list of 25 ETEs, with 24 of them belonging to “Category 6” and one to “Category 1” (Table 2). While almost all the ETEs in “Category 6” (24/26) met both filtering criteria for one abiotic stress condition, half of them (12/24) met the *P*-value filtering of 0.01 for more than one stress condition (Table 2). This suggests that while some ETEs may play a role related to a specific type of stress, others may be responsive to stress conditions in general.

**Table 2:** Summary of stress exapted TEs with at least 2 T-DNA lines.

					**Salt**	**Arsenic**	**Phosphate**	**Freezing**
**Locus**	**Exapted TE**	**Locus name**	**Superfamily of**	**Category (see**	**% survival (*P*-value)**	**% germination (*P*-value)**	**Avg. root length (*P*-value)**	**% + 50% damage (*P*-value)**
	**family**		**ancestral TE**	**Figure 4)**				
Wild–type	–	–	–		66.00%	64.00%	345.54	50% (2011) 74% (2013)
At1g21260			LTR	Cat. 6		26% (1.20E−07) 38% (4.89E−04)		
At2g27110	*FRS3*	*FRS3*	MULE	Cat. 1		88% (1.94E−04) 47% (3.58E−02)		
At1g06740	*MUGA*	*MUG3*	MULE	Cat. 6	43% (1.34E−03) 50% (2.73E−02)			
At1g15300	–	–	hAT	Cat. 6	43% (1.34E−03) 43% (1.34E−03)			
At1g64255	–	–	MULE	Cat. 6	28% (2.37E−07) 48% (1.06E−02)			
At1g69950	–	–	hAT	Cat. 6	15% (6.45E−13) 29% (3.58E−07) 36% (2.28E−05)			41% (8.59E−05) (2013)
At1g80020	–	–	hAT	Cat. 6	25% (1.82E−08) 34% (1.16E−05) 47% (1.06E−02)			87% (2.60E−05) (2011) 47% (1.15E−03) (2013)
At2g30640	*MUGA*	*MUG2*	MULE	Cat. 6	27% (7.55E−08) 32% (8.95E−06)	6% (2.66E−19) 8% (2.69E−17)		
At3g04605	*MUGA*	*MUG1*	MULE	Cat. 6	21% (4.15E−10) 22% (1.04E−09)	14% (2.00E−13) 17% (1.66E−09)		
At3g13020	–	–	hAT	Cat. 6	39% (3.15E−04) 40% (3.62E−04) 50% (2.04E−02)	37% (4.89E−04) 46% (3.59E−02)		
At3g13030	–	–	hAT	Cat. 6	35% (2.16E−05) 47% (1.06E−02) 49% (1.47E−02)		513.87 (5.91E−03) 451.20 (2.57E−02) 409.42 (2.99E−02)	
At3g17450	–	–	hAT	Cat. 6	35% (2.16E−05) 49% (2.04E−02)		679.03 (5.41E−06) 552.01 (3.42E−03)	
At3g22170	*FHY3*	*FHY3/ CPD45*	MULE	Cat. 6	26% (4.02E−08) 31% (1.60E−06)			
At3g22220	–	–	hAT	Cat. 6	46% (7.79E−03) 50% (2.82E−02)		490.36 (8.83E−03) 391.97 (4.95E−02)	
At3g55350	–	–	PIF–Harbinger	Cat. 6	23% (7.96E−09) 41% (5.39E−04)	10% (6.28E−16) 15% (6.42E−13)		
At4g13120	–	–	hAT	Cat. 6	10% (1.51E−16) 41% (5.44E−04) 43% (1.34E−03)		563.49 (6.04E−03) 445.79 (2.67E−02)	
At4g19990	*FHY3*	*FRS1*	MULE	Cat.6	40% (5.44E−04) 48% (1.45E−02) 49% (1.47E−02)	37% (4.89E−04) 40% (1.34E−03)	554.35 (2.25E−05) 470.36 (6.04E−03)	
At5g16505	*MUGA*	*MUG4*	MULE	Cat. 6	13% (2.67E−14) 19% (3.47E−11)			
At1g10240	*FRS10*	*FRS11*	MULE	Cat. 6			483.80 (6.04E−03) 483.14 (9.04E−03)	
At1g52520	*FRS6*	*FRS6*	MULE	Cat. 6			506.89 (8.31E−04) 442.00 (1.28E−02)	
At1g76320	*FHY3*	*FRS4/CPD25*	MULE	Cat. 6			452.98 (2.97E−03) 444.35 (3.53E−03)	
At3g06250	*FRS7*	*FRS7*	MULE	Cat. 6	27% (4.13E−07) 35% (1.37E−05)			
At5g34853	*MUGB*	*MUG8*	MULE	Cat. 6				17% (1.07E−03) (2011) 19% (1.10E−03) (2011) 47% (1.15E−03) (2013)
At5g41980	–	–		Cat. 6	16% (5.40E−09) 46% (7.79E−03)			
At5g50315	–	–		Cat. 6	34% (1.90E−05) 38% (1.15E−04)	23% (9.42E−09) 34% (3.23E−05)		

*Table showing the list of ETEs that were considered having strong and robust phenotypic responses under one or more abiotic stress conditions. To be included, at least 2 T-DNA lines needed to be significant (P < 0.05) for the same assay, and additionally, at least one of them needed to have a P-value lower than 0.01. Genes belonging to Category 6 showed significant phenotypes under more than one assay and if only one stress is showed, indicates that the lines did not have a P-value lower than 0.01 for that stress. All the P-values can be found in Table S2. The names of the exapted TE families follow the phylogeny as shown in Joly-Lopez et al. (2016)*.

From the ETEs listed in Table 2, we have detected previously unreported phenotypic responses under abiotic stress conditions for a number of well-characterized ETEs. Among the known ETEs, we found that all four members of the *MUGA* gene family have a salt tolerance phenotype and, in addition, *MUG1* (At3g04605) and *MUG2* (At2g30640) have a mutant phenotype in the arsenic assay. Moreover, the related *FHY3* ETE (At3g22170) also has a salt tolerance phenotype, whereas *FAR1-related sequences* (*FRS*) genes have a phosphate limiting phenotype. *FRS1* is the only ETE for which we found a strong mutant phenotype under three abiotic stress conditions (i.e., salt, arsenic, and phosphate) (Table 2). With respect to the *FAR1/FHY3* gene family, it is worth mentioning that even though the ETE *FAR-RED IMPAIRED RESPONSE* (*FAR1;* At4g15090) did exhibit a significant mutant phenotype in three assays (i.e., salt, arsenic, and phosphate), it was not included in the filtered list as only one previously uncharacterized T-DNA line was available when we conducted this study (Table 3). A number of other ETEs, belonging to “Category 1” and “Category 3” also had one T-DNA mutant line available for this study, but based on their phenotype, it would be worth conducting subsequent experiments with additional T-DNA mutant lines for these genes (Table 3). The ETE gene families *MUGA, MUGB*, and *FAR1/FHY3* are all derived from *Mutator*-like elements (MULEs) DNA transposons (Yu et al., 2000; Lisch et al., 2001; Cowan et al., 2005). Of the 26 novel ETEs tested in this study, 13 passed the final triage, excluding four where only one T-DNA mutant line was available (Tables 2, 3). Although the majority of them are derived from *h*AT and Pif-Harbinger DNA transposons, we also identified one ETE derived from a Ty1/*copia* retrotransposon (At1g21260). Nine of these novel ETEs present in the final triage have strong phenotypes in more than one assay (Table 2). Taken together, the identified and previously reported phenotypes suggest that many of the triaged ETEs are pleiotropic (Ouyang et al., 2011). Stress-related phenotype among members of the same ETE family were also detected, as in the case of *MUGA* (salt) and *FAR1*/*FHY3* (phosphate), which is also observed for TE-mediated stress induced gene expression (Makarevitch et al., 2015).

**Table 3:** Summary of stress exapted TEs with only 1 T-DNA line.

					**Salt**	**Arsenic**	**Phosphate**	**Freezing**
**Locus**	**Exapted TE family**	**Locus name**	**Superfamily of ancestral TE**	**Category (see Figure 4)**	**% survival (*****P*****-value)**	**% germination (*****P*****-value)**	**Avg. root length (*****P*****-value)**	**%** + **50% damage (*****P*****-value)**
At3g06940	*MUGB*	*MUG5*	MULE	Cat. 3	31% (1.60E-06)	29% (1.09E-06)		
At3g13010			hAT	Cat. 3	38% (1.20E-04)	36% (8.57E-04)		
At4g15090	*FHY3*	*FAR1*	MULE	Cat. 3	43% (1.34E-03)	40% (1.34E-03)	524.58 (6.79E-04)	
At4g38170	*FRS3*	*FRS9*	MULE	Cat. 3	44% (4.00E-03)	28% (5.47E-07)		
At5g48965	*MUGB*	*MUG6*	MULE	Cat. 1	46% (5.33E-03)			
At5g20880			LTR	Cat. 3	47% (7.79E-03)		495.58 (7.30E-03)	
At3g17290			hAT	Cat. 1	47% (1.06E-02)			
At5g28530	*FRS10*	*FRS10*	MULE	Cat. 3	49% (2.03E-02)		474.69 (1.63E-02)	
At1g79740			hAT	Cat. 3			543.92 (4.59E-05)	
At2g32250	*FHY3*	*FRS2*	MULE	Cat. 1			545.35 (6.04E-03)	

*Table showing the list of ETEs belonging to Category 3, which showed significant phenotypes but for which only one T-DNA line was available in this study. The names of the exapted TE families follow the phylogeny as shown in Joly-Lopez et al. (2016)*.

## Discussion

The overall goal of this study was to use a reverse genetics approach coupled with phenomics to assess the phenotypic functionality for putative protein-coding ETEs (Hoen and Bureau, 2015), under four abiotic stresses of agricultural importance. Our results show that we can successfully uncover new phenotypes for known and novel ETEs using our approach, and that a large number of *A. thaliana* ETE mutants exhibit significant phenotypes under one or more of these four stresses, suggesting that these ETEs are functional.

We opted for a phenomics approach to increase the throughput, accuracy, precision, and objectivity of the analysis of the mutant phenotypes. Indeed, the phenomics platform can increase the repeatability of the measurements by applying the same optical, lighting, and spatial conditions as can be observed in our phenotypic database (http://mp3.biol.mcgill.ca/dteassays). This advantage allowed us to impartially and exactly quantify plant phenotypes through computer vision algorithms such as clustering of color classification or morpho-colorimetric analysis successfully used in this and other studies (Skirycz et al., 2010; Plessis et al., 2011; Hairmansis et al., 2014; Poire et al., 2014; Humplik et al., 2015; Neilson et al., 2015).

In a recent publication, Ubbens and Stavness (2017) highlighted the genotype-to-phenotype gap that persists in modern plant breeding: current phenotyping capacity lags far behind the influx of genomic data generated by next generation sequencing (NGS) technologies (Yang et al., 2014). Consequently, new advances are needed both in image capture technologies (cameras, robotics, conveyor systems, etc.), in image processing algorithms to transform images into useful quantitative measurements of phenotypes, and techniques such as machine learning to analyze these measurements, such as the Deep Plant Phenomics platform, an open-source tool providing pre-trained neural networks for common plant phenotyping tasks (Ubbens and Stavness, 2017). Furthermore, most currently available image analysis pipelines are geared toward pot-grown plants, so there is opportunity for important advances in the analysis of plate-grown seedlings, a particularly important approach since seedlings can be grown and measured far more quickly than adult plants, helping to alleviate the genotype-to-phenotype gap. In this study, we developed several image-based phenotyping pipelines customized for each abiotic stress assay and show their efficacy in detecting the phenotypic impacts of genomic mutations.

Overall, T-DNA mutant lines revealed to be significant using a main phenotypic trait analysis also clustered together when analyzing multiple morpho-colorimetric phenotypic traits. The fact that none of these lines clustered with wild-type suggests that these significant T-DNA lines have a phenotypic profile that is distinct (Figures S3–S7). While most of the ETEs for which two T-DNA lines were available were found in the same cluster, we found five instances in the salt heatmap in which two T-DNA mutant lines were found in different groups of clusters (lines 11, 14, 57, 39 in ω; 48 in “σ”) (Figure 3, Table S3). These differences are likely due to the fact that each T-DNA mutant line is independent and thus yield plants that are not significantly different from one another when we consider a main phenotypic trait, but can be detected using multiple features. If we take, for example, the ETE At1g69950, its two T-DNA lines under salt stress are in distinct clusters (73 in “β”; 11 in “ω”). It is understandable why when we look at their phenotypic profiles (Figure S4), where the phenotype of line 73 is characterized by a larger hisgreypeak, while the phenotype of line 11 is characterized by a larger major and minor axes.

Our results revealed that more than 86% of the T-DNA mutant lines displayed a phenotypic response in at least one stress condition (Table S2). The presence of a phenotype for the novel ETEs is consistent with these sequences derived from TEs having departed from their once mobile lifestyle for one beneficial to the host. In other words, the phenotypic response suggests that the null hypothesis that the candidates tested are TEs with no host function is rejected. Also, this study has revealed the presence of external phenotypes for these ETEs, using a phenomics approach coupled with genomic tools, and future studies should look at the internal phenotype, such as the cellular function and molecular phenotyping (e.g., metabolomics, proteomics, and transcriptomics).

To highlight ETEs that showed a strong response specific to the abiotic stresses tested in this study, we applied two filters based on the number of T-DNA lines and the statistical strength of the phenotype, and obtained a list of 25 ETEs (Figure 4, Table 2). Although subsequent experiments will provide more details as to whether these phenotypes are induced or constitutive, the strength of the response observed for some mutant lines suggests that they play previously unsuspected but important roles under these stress conditions. Within the stringent subset, we found a number of known ETEs that displayed a very marked phenotype in the stress assays, with phenotypes sometimes comparable to those of the reference genes used in our study. In the case of the well-characterized *FHY3* family of ETEs, several approaches, including genome-wide DNA binding site analysis (e.g., ChIP-seq) for *FHY3*, have revealed that *FAR1* and *FHY3* encode proteins that regulate the transcription of hundreds of genes that are not only involved in light signaling, but also in circadian pathways and many other facets of plant development (Li et al., 2011; Ouyang et al., 2011). Furthermore, genes from the *MUGA* family may also be involved in the regulation of different genes in various pathways, as suggested from differential gene expression analysis and preliminary DNA binding site analysis (Joly-Lopez, unpublished data). Based on this, it may not be surprising that these known ETEs displayed a strong phenotype in our stress assays as they have already displayed pleiotropic phenotypes besides the ones uncovered in this study (Joly-Lopez and Bureau, 2014; Wang and Wang, 2015). A number of the novel, previously functionally uncharacterized, ETEs also display strong phenotypes and warrant further investigation.

Among the four stress conditions that we tested, the high salt assay accounted for the majority of the stringent subset of mutant lines. A possible explanation is that this assay was suitable to uncover not only genes associated with responses to salt stress specifically, but also genes that are associated with other osmotic stresses such as drought, heat, and cold. Indeed, although salinity, and dehydration are broadly defined as an osmotic stress and cold and freezing are more specifically defined as temperature stresses, there is overlap between the responsiveness of many genes involved in these two stress categories (Thomashow, 1998; Zhu, 2002; Munns and Tester, 2008). Therefore, some of these ETEs may have a function associated with a specific stress response, while others may be associated with multiple stresses.

When we started this study, we had hypothesized that the novel ETEs were probably functional in *A. thaliana*, but we did not have expectations about their phenotypes under the abiotic stresses we tested, as well as for the known ETEs for which abiotic stress phenotypes were mostly unreported. Of the 50 ETEs we tested, 46 of them (93/103 T-DNA mutant lines) showed a significant phenotype in one or more of the stresses (Figures 2, 4). Among these 46 genes, only four of them (4/93 T-DNA mutant lines) showed phenotypic differences under standard growth conditions, which suggest that the remaining 42 ETEs appear to be phenotypically different specifically under our stress conditions. The subset of 25 ETEs for which a robust link between genotype and phenotype was highlighted in this study corresponded to 56 of the 103 T-DNA mutant lines (Table 2, Table S2). When compared to other large screens conducted in *A. thaliana* to uncover mutants responsive to abiotic stresses, it appears that we may have detected more T-DNA mutant lines. For example, in two studies involving the screening of 250,000 (Koiwa et al., 2006) and 40,000 (Pan et al., 2012) randomly chosen T-DNA mutant lines, of these, 200 and 121 mutants, respectively, were found to display significant changes in responses to abiotic stresses (i.e., salinity and ABA sensitivity), which correspond to about 0.08% and 0.3% of the total number of respective lines (Koiwa et al., 2006; Pan et al., 2012). Another study (Luhua et al., 2013) used T-DNA mutant lines corresponding to selected protein-coding genes of unknown function with differential gene expression under abiotic stresses (Horan et al., 2008), and they found that 12–31% of the mutants displayed a phenotype in at least one stress condition (i.e., heat, salinity, osmotic, UV-B, and cold). These results show that, compared to other host genes, ETEs have a strong tendency to be associated with phenotypic changes under stress conditions. One possibility that could explain this bias may come from the apparent inherent response of TEs to stress conditions or environmental cues (Grandbastien, 1998; Huang et al., 2012). Indeed, large increases in TE activity in plants have been associated with stresses including extreme temperatures (Barah et al., 2013; Cavrak et al., 2014; Makarevitch et al., 2015), chemical exposure (Knight et al., 1996; Castrillo et al., 2013; Makarevitch et al., 2015), and nutrient challenges (Maumus et al., 2009; Castrillo et al., 2013). Despite their mutagenic capacity, irreversible genome restructuring following TE activation is a central tenant of the “genome shock” model of adaptation to environmental challenges, first alluded to by McClintock (1984). Consistent with this model is recent evidence that TE proliferation modifies or rewires regulatory networks altering local to global gene expression patterns (Casacuberta and Gonzalez, 2013; Oliver et al., 2013). In addition, many protein-coding ETEs are thought to be transcription factors giving rise to novel regulons, likely enlisting suites of TE-derived *cis*-factors (Bourque et al., 2008; Feschotte, 2008).

The expression of many of the same TE families also appeared to respond to the same stress conditions (Makarevitch et al., 2015), a trend that was also detected in our results at the phenotypic level for ETEs belonging to same family (e.g., *MUGA*) and in some cases that have been exapted from similar TE sequences (Table 2). For example, all the genes belonging to the *FHY3* family have a phosphate-limitation phenotype, as well as many genes from *FRS* families (Tables 2, 3, Table S2). Furthermore, all four *MUGA* genes are associated with salt stress, and *MUG1* and *MUG2*, which belong to the same family subclade (Joly-Lopez et al., 2016), are also associated with arsenic toxicity. It could be possible that these inherent TE characteristics may have patterned the functionalization of emerging ETEs. Over evolutionary time, fixed ETEs may have evolved diversified roles, but they could have also retained inherent characteristics of their TE ancestral states, such as their response to stress.

In conclusion, we have shown that combining mature resources and methodologies in determining gene function (functional genomics) along with cutting-edge tracking of abiotic stress responses (phenomics and bioinformatics) is successful to uncover known and novel conserved genes derived from TEs that have phenotypes associated with abiotic stresses. In addition to reporting new mutant phenotypes for known ETEs, we have shown that a number of novel ETEs may play important functions in *A. thaliana*. With these ETEs being conserved between multiple Brassicaceae species, it is possible that these functions may likewise be conserved. While we were interested in sequences derived from TEs, our strategy to use a mixed approach of reverse genetics, phenomics, and computer vision should be easily extended to any category of genes. The strategy of using biotechnological approaches and targeting uncharacterized DNA as proposed in this study is an important contribution that could be applied in the overall effort to “leaving no stone unturned” in the mitigation of impending effects of climate changes and in the development of new sustainable agricultural strategies.

## Author contributions

TB: conceived and designed the project. TB and ZJ-L: wrote the manuscript. EF and ZJ-L: designed and performed the experiments; EV performed the phenotyping analyses; DH: assisted in the co-opted TE target selection; AT: performed the genotyping and provided technical assistance. ZJ-L, EF, and EV: contributed equally to the study. All authors discussed the results and provided input on the manuscript.

### Conflict of interest statement

The authors declare that the research was conducted in the absence of any commercial or financial relationships that could be construed as a potential conflict of interest.
