# Supplementary material for: Abiotic Stress Phenotypes Are Associated with Conserved Genes Derived from Transposable Elements
Source: Front Plant Sci. 2017 Nov 28;8:2027. doi: 10.3389/fpls.2017.02027 (PMC5715367; doi:10.3389/fpls.2017.02027)
Supplement: Supplementary file 4 [file Image1.PDF]

Supplementary Figure 1. Location of the T-DNA insertions with respect to each ETE gene. Snapshots from the TAIR's genome browser (http://gbrowse.arabidopsis.org/cgi-bin/gbrowse/arabidopsis) representing the location of each ETE gene in the Arabidopsis thaliana genome. Each ETE is highlighted in yellow and the location of the T-DNA is shown on the track "T-DNA/Transposons. The list of T-DNA lines used in this study, as well as the genomic coordinate of the insertions is shown in Table S1.

AT1G15300

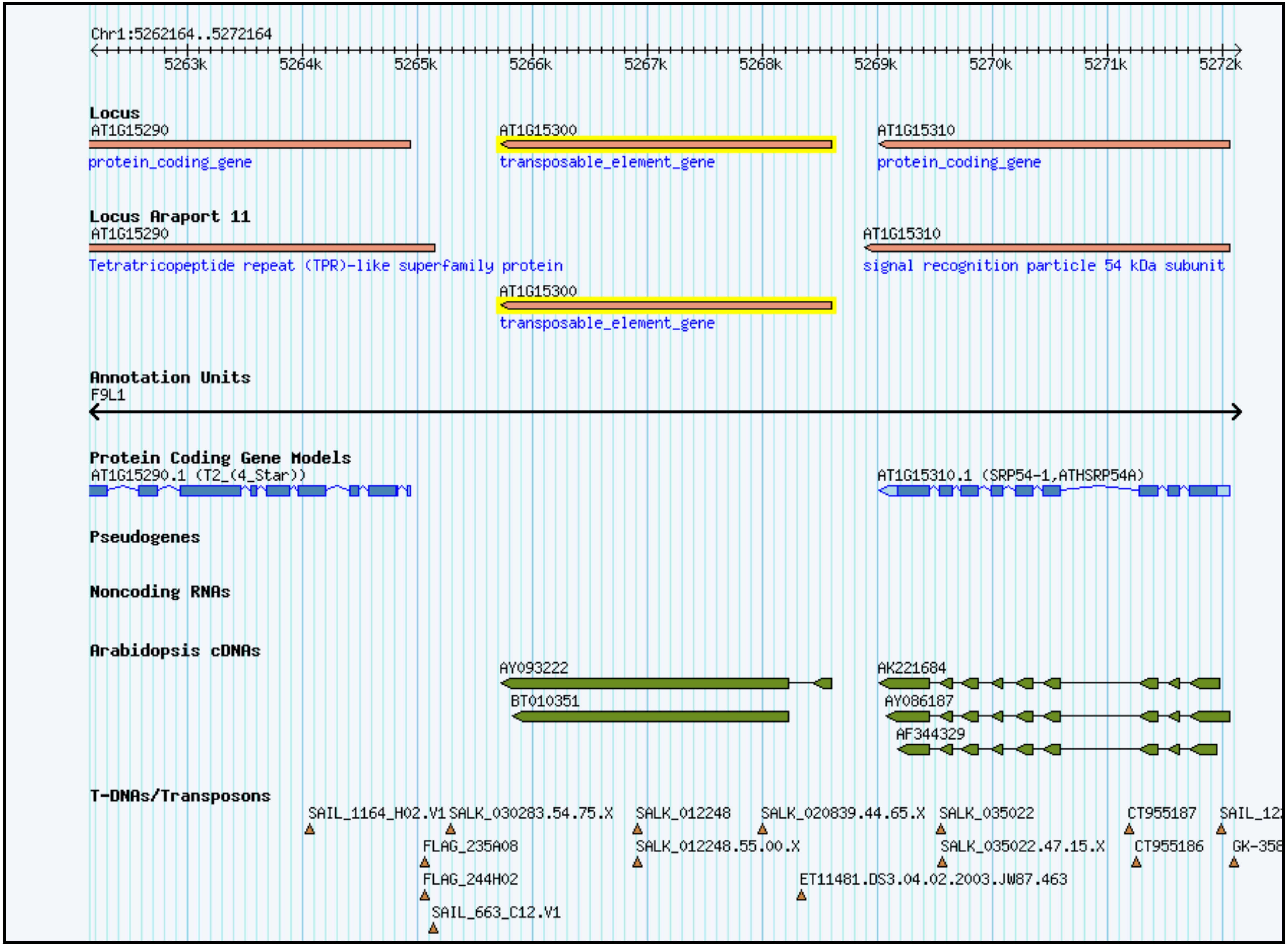

AT1G10240

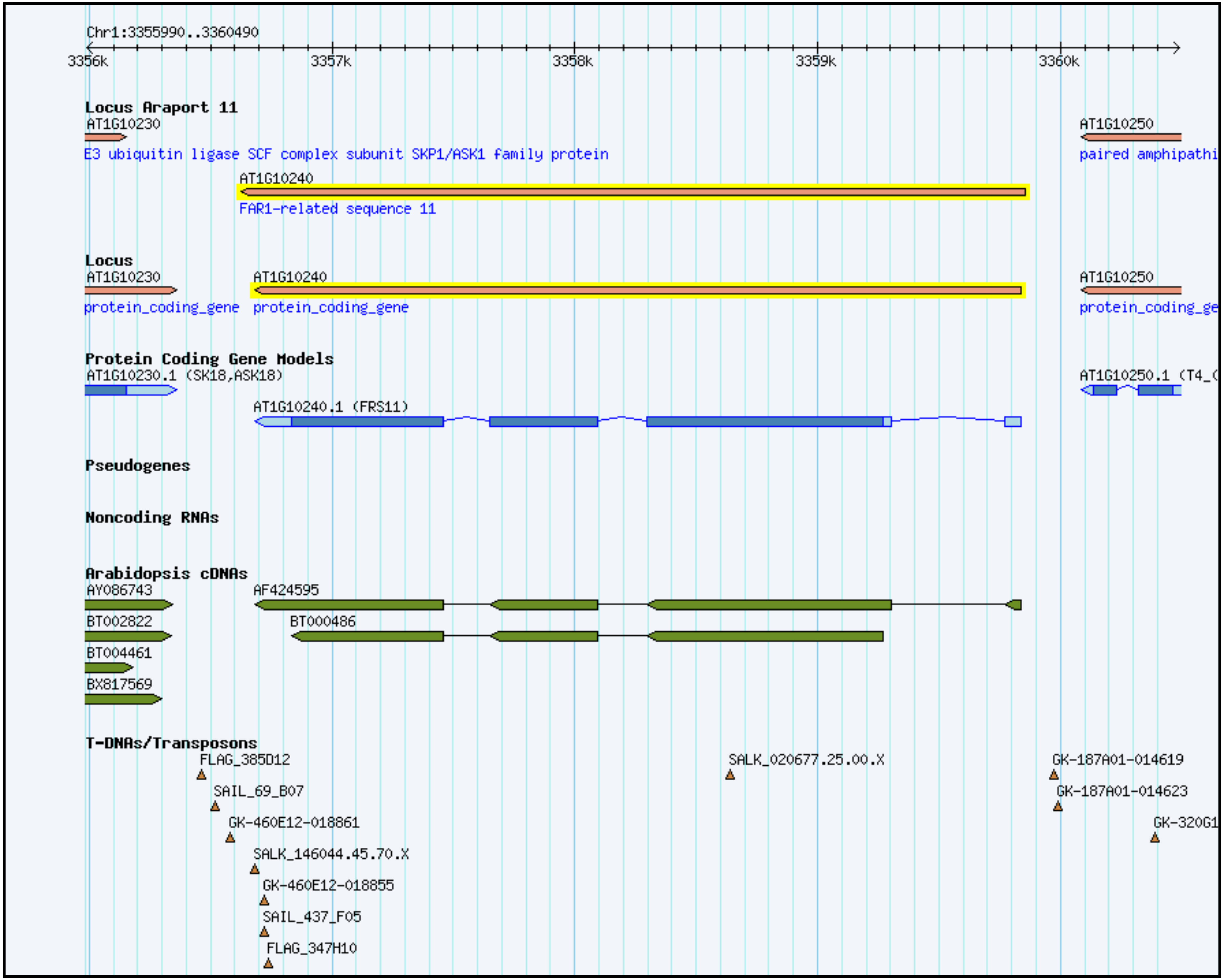
Chr1:3355990..3360490
3356k
3357k
3358k
3359k
3360k
Locus Araport 11
AT1G10230
E3 ubiquitin ligase SCF complex subunit SKP1/ASK1 family protein
AT1G10250
paired amphipathi
AT1G10240
FAR1-related sequence 11
Locus
AT1G10230
protein_coding_gene
AT1G10240
protein_coding_gene
AT1G10250
protein_coding_ge
Protein Coding Gene Models
AT1G10230.1 (SK18,ASK18)
AT1G10250.1 (T4_(
AT1G10240.1 (FRS11)
Pseudogenes
Noncoding RNAs
Arabidopsis cDNAs
AY086743
AF424595
BT002822
BT000486
BT004461
BX817569
T-DNAs/Transposons
FLAG_385D12
SALK_020677.25.00.X
GK-187A01-014619
SAIL_69_B07
GK-187A01-014623
GK-460E12-018861
GK-320G1
SALK_146044.45.70.X
GK-460E12-018855
SAIL_437_F05
FLAG_347H10

AT1G18560

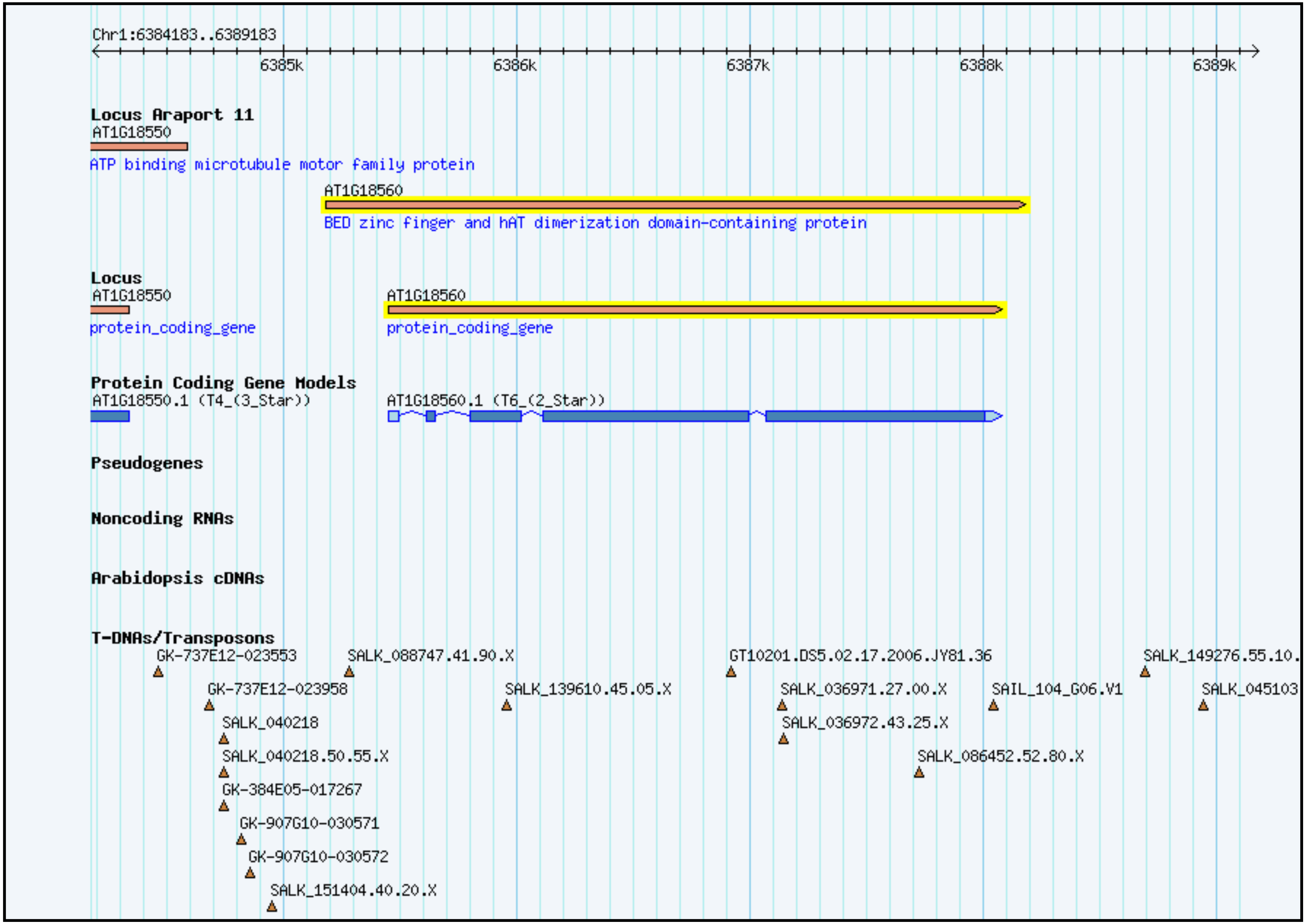
Chr1:6384183..6389183
6385k
6386k
6387k
6388k
6389k
Locus Araport 11
AT1G18550
ATP binding microtubule motor family protein
AT1G18560
BED zinc finger and hAT dimerization domain-containing protein
Locus
AT1G18550
protein_coding_gene
AT1G18560
protein_coding_gene
Protein Coding Gene Models
AT1G18550.1 (T4_(3_Star))
AT1G18560.1 (T6_(2_Star))
Pseudogenes
Noncoding RNAs
Arabidopsis cDNAs
T-DNAs/Transposons
GK-737E12-023553
SALK_088747.41.90.X
GT10201.DS5.02.17.2006.JY81.36
SALK_149276.55.10.
GK-737E12-023958
SALK_139610.45.05.X
SALK_036971.27.00.X
SAIL_104_G06.V1
SALK_045103
SALK_040218
SALK_036972.43.25.X
SALK_040218.50.55.X
SALK_086452.52.80.X
GK-384E05-017267
GK-907G10-030571
GK-907G10-030572
SALK_151404.40.20.X

AT1G21260

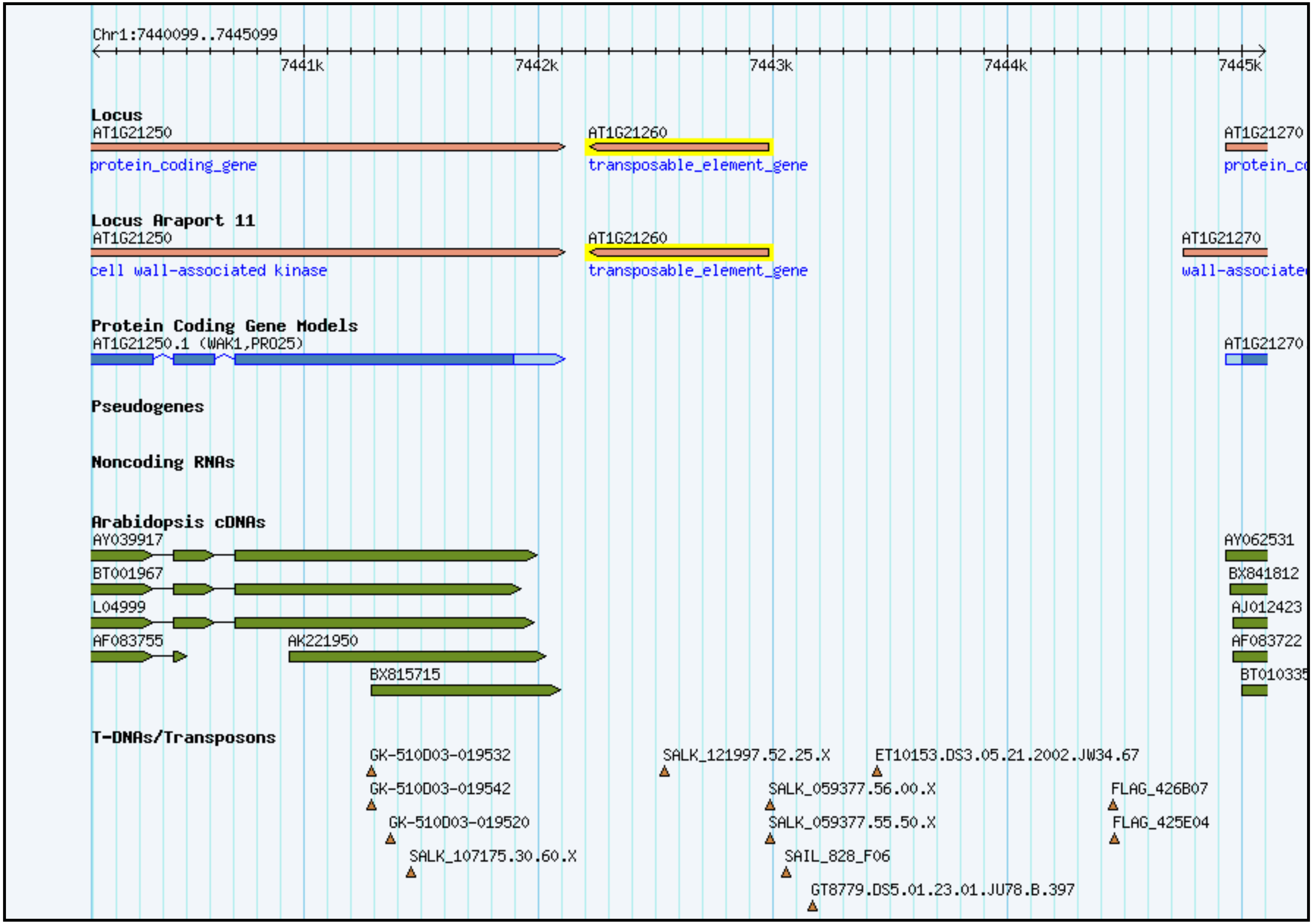

Chr1:7440099..7445099
7441k
7442k
7443k
7444k
7445k
Locus
AT1G21250
protein_coding_gene
AT1G21260
transposable_element_gene
AT1G21270
Locus Araport 11
AT1G21250
cell wall-associated kinase
AT1G21260
transposable_element_gene
AT1G21270
Protein Coding Gene Models
AT1G21250.1 (WAK1,PRO25)
AT1G21270
Pseudogenes
Noncoding RNAs
Arabidopsis cDNAs
AY039917
BT001967
L04999
AF083755
AK221950
BX815715
AY062531
BX841812
AJ012423
AF083722
T-DNAs/Transposons
GK-510D03-019532
GK-510D03-019542
GK-510D03-019520
SALK_107175.30.60.X
SALK_121997.52.25.X
SALK_059377.56.00.X
SALK_059377.55.50.X
SAIL_828_F06
GT8779.DS5.01.23.01.JU78.B.397
ET10153.DS3.05.21.2002.JW34.67
FLAG_426B07
FLAG_425E04

AT1G21280

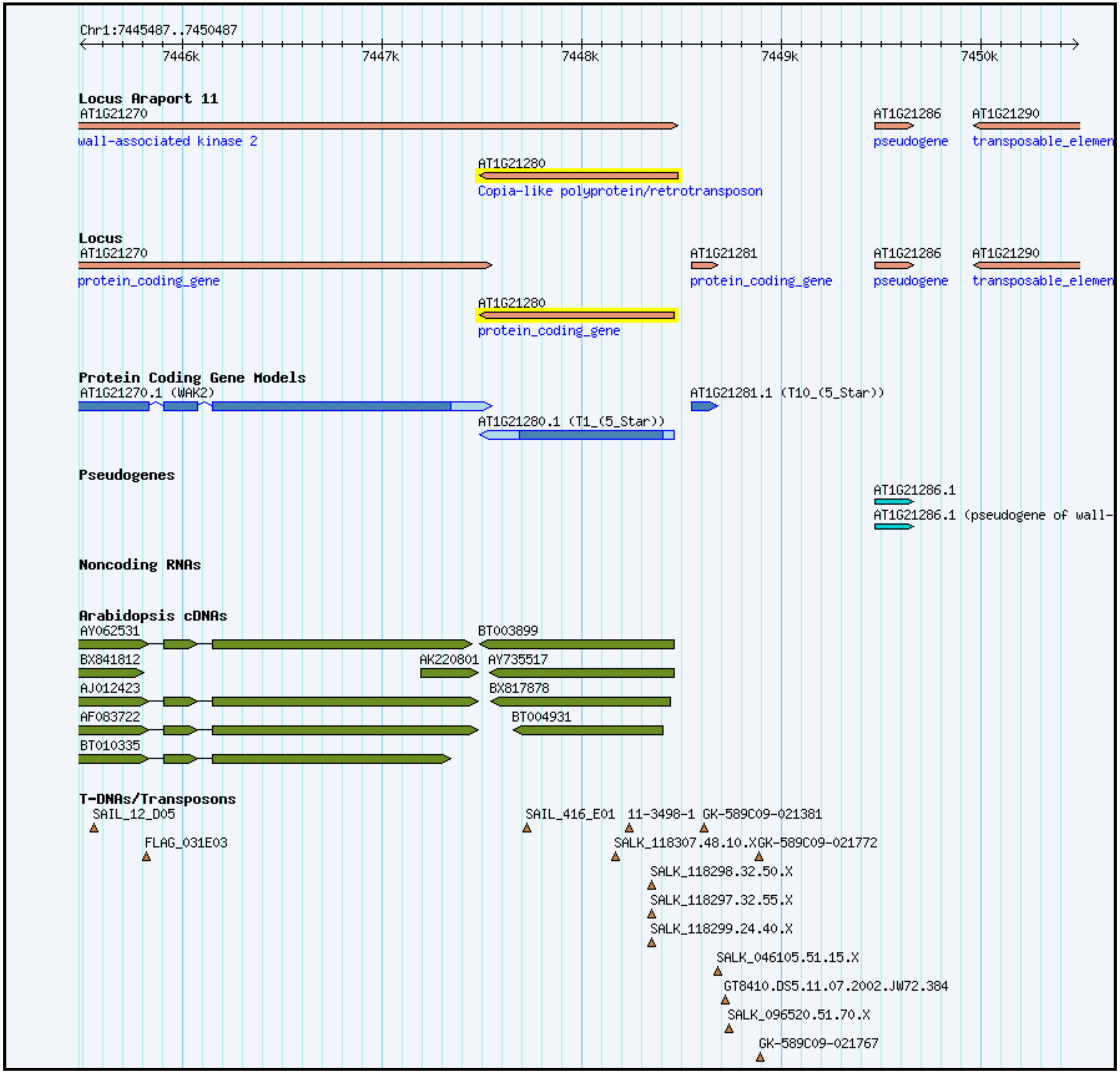
Chr1:7445487..7450487
7446k
7447k
7448k
7449k
7450k
Locus Araport 11
AT1G21270
wall-associated kinase 2
AT1G21286
pseudogene
AT1G21290
transposable_elemen
AT1G21280
Copia-like polyprotein/retrotransposon
Locus
AT1G21270
protein_coding_gene
AT1G21281
protein_coding_gene
AT1G21286
pseudogene
AT1G21290
transposable_elemen
AT1G21280
protein_coding_gene
Protein Coding Gene Models
AT1G21270.1 (WAK2)
AT1G21281.1 (T10_(5_Star))
AT1G21280.1 (T1_(5_Star))
Pseudogenes
AT1G21286.1
AT1G21286.1 (pseudogene of wall-
Noncoding RNAs
Arabidopsis cDNAs
AY062531
BT003899
BX841812
AK220801
AY735517
AJ012423
BX817878
AF083722
BT004931
BT010335
T-DNAs/Transposons
SAIL_12_D05
SAIL_416_E01
11-3498-1
GK-589C09-021381
FLAG_031E03
SALK_118307.48.10.X
GK-589C09-021772
SALK_118298.32.50.X
SALK_118297.32.55.X
SALK_118299.24.40.X
SALK_046105.51.15.X
GT8410.DS5.11.07.2002.JW72.384
SALK_096520.51.70.X
GK-589C09-021767

AT1G21290

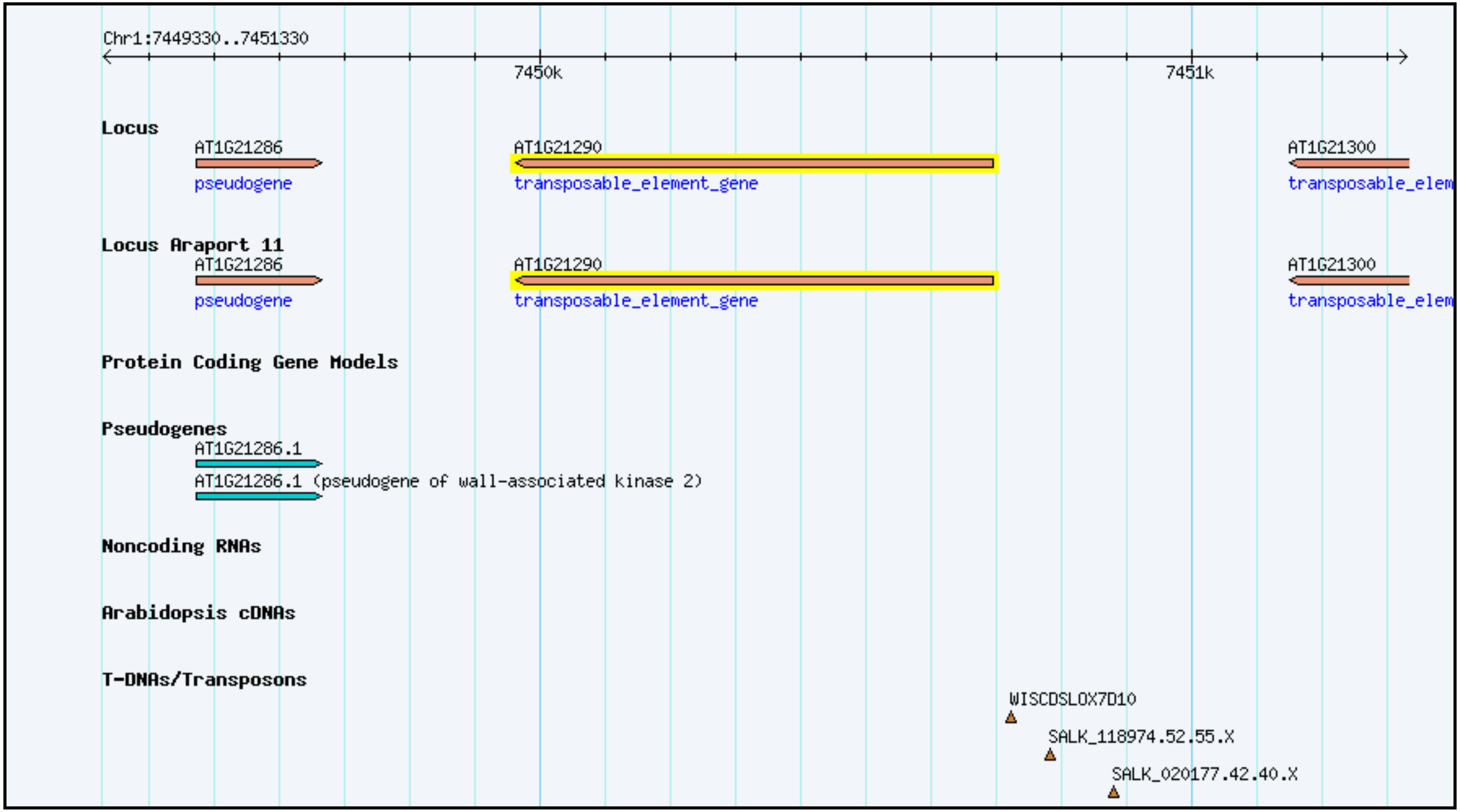
Chr1:7449330..7451330
7450k
7451k
Locus
AT1G21286
pseudogene
AT1G21290
transposable_element_gene
AT1G21300
transposable_elem
Locus Araport 11
AT1G21286
pseudogene
AT1G21290
transposable_element_gene
AT1G21300
transposable_elem
Protein Coding Gene Models
Pseudogenes
AT1G21286.1
AT1G21286.1 (pseudogene of wall-associated kinase 2)
Noncoding RNAs
Arabidopsis cDNAs
T-DNAs/Transposons
WISCDSLOX7D10
SALK_118974.52.55.X
SALK_020177.42.40.X

AT1G49920

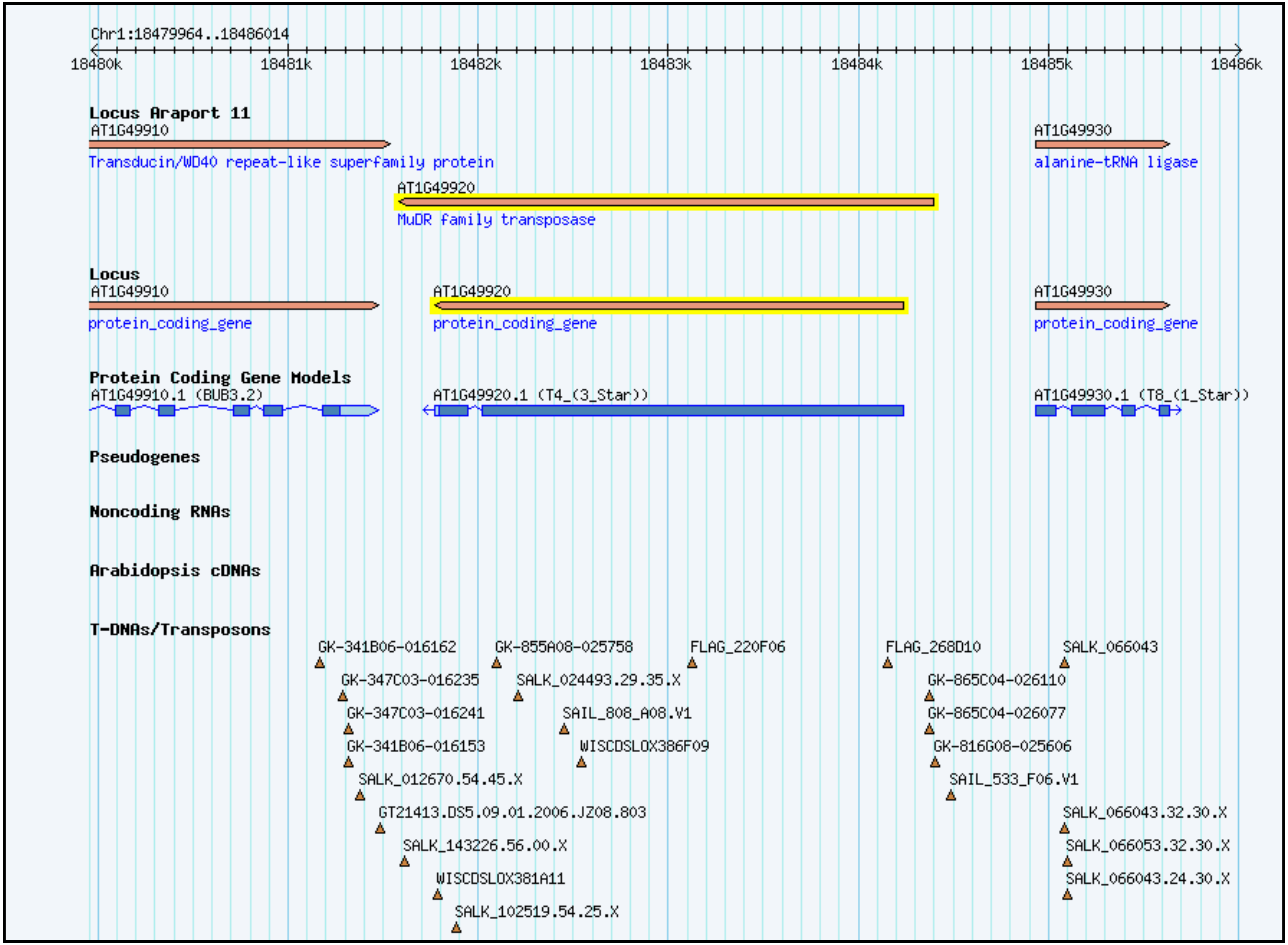

Chr1:18479964..18486014
18480k
18481k
18482k
18483k
18484k
18485k
18486k
Locus Araport 11
AT1G49910
Transducin/WD40 repeat-like superfamily protein
AT1G49930
alanine-tRNA ligase
AT1G49920
MuDR family transposase
Locus
AT1G49910
protein_coding_gene
AT1G49920
protein_coding_gene
AT1G49930
protein_coding_gene
Protein Coding Gene Models
AT1G49910.1 (BUB3.2)
AT1G49920.1 (T4_(3_Star))
AT1G49930.1 (T8_(1_Star))
Pseudogenes
Noncoding RNAs
Arabidopsis cDNAs
T-DNAs/Transposons
GK-341B06-016162
GK-855A08-025758
FLAG_220F06
FLAG_268D10
SALK_066043
GK-347C03-016235
SALK_024493.29.35.X
GK-865C04-026110
GK-347C03-016241
SAIL_808_A08.V1
GK-865C04-026077
GK-341B06-016153
WISCDSLOX386F09
GK-816G08-025606
SALK_012670.54.45.X
SAIL_533_F06.V1
GT21413.DS5.09.01.2006.JZ08.803
SALK_066043.32.30.X
SALK_143226.56.00.X
SALK_066053.32.30.X
WISCDSLOX381A11
SALK_066043.24.30.X
SALK_102519.54.25.X

AT1G52520

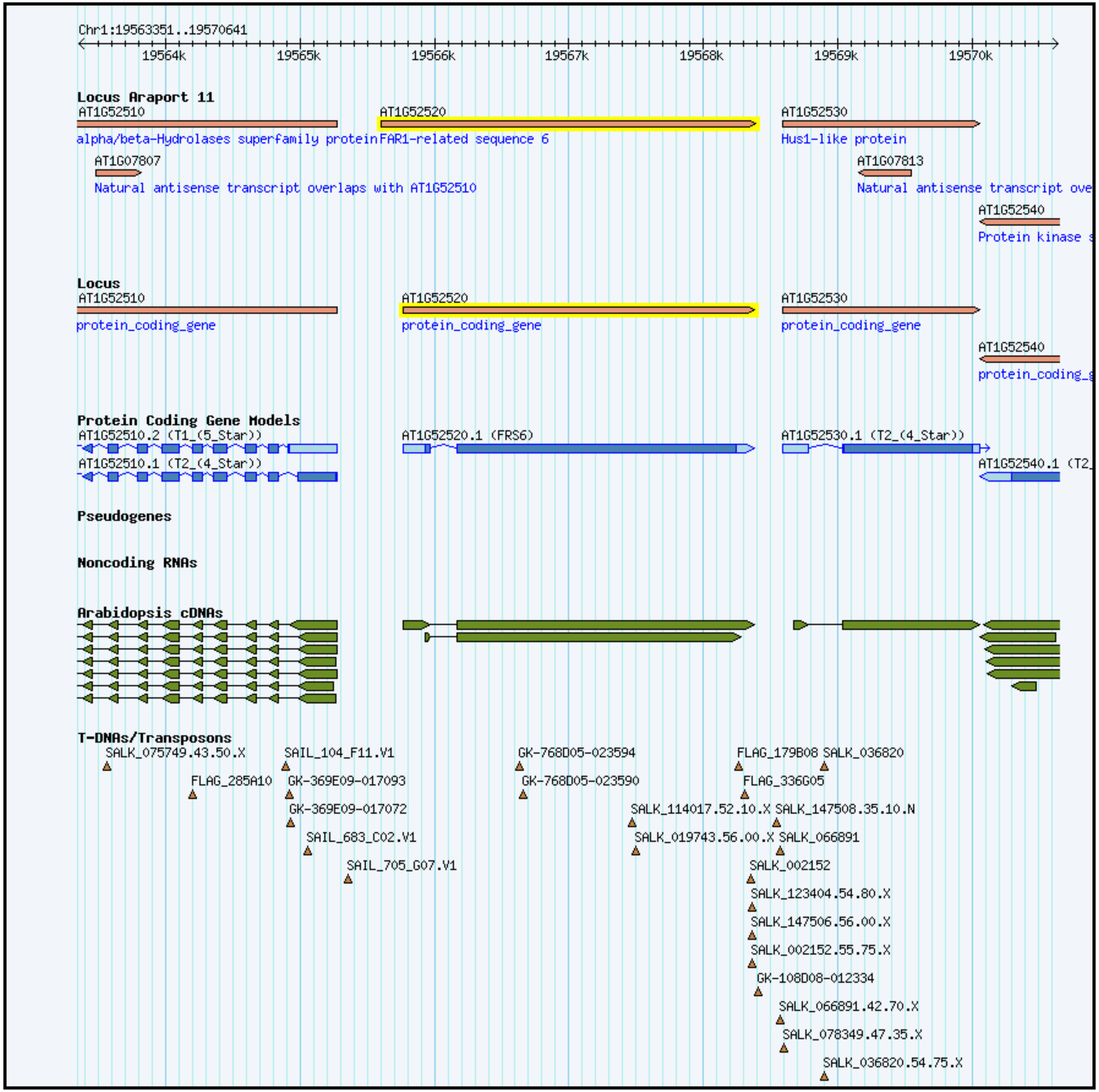

Chr1:19563351..19570641
19564k
19565k
19566k
19567k
19568k
19569k
19570k
Locus Araport 11
AT1G52510
alpha/beta-Hydrolases superfamily protein
AT1G52520
FAR1-related sequence 6
AT1G52530
Hus1-like protein
AT1G07807
Natural antisense transcript overlaps with AT1G52510
AT1G07813
AT1G52540
Locus
AT1G52510
protein_coding_gene
AT1G52520
protein_coding_gene
AT1G52530
protein_coding_gene
AT1G52540
Protein Coding Gene Models
AT1G52510.2 (T1_(5_Star))
AT1G52510.1 (T2_(4_Star))
AT1G52520.1 (FRS6)
AT1G52530.1 (T2_(4_Star))
Pseudogenes
Noncoding RNAs
Arabidopsis cDNAs
T-DNAs/Transposons
SALK_075749.43.50.X
FLAG_285A10
SAIL_104_F11.V1
GK-369E09-017093
GK-369E09-017072
SAIL_683_C02.V1
SAIL_705_G07.V1
GK-768D05-023594
GK-768D05-023590
SALK_114017.52.10.X
SALK_019743.56.00.X
FLAG_179B08
SALK_036820
FLAG_336G05
SALK_147508.35.10.N
SALK_066891
SALK_002152
SALK_123404.54.80.X
SALK_147506.56.00.X
SALK_002152.55.75.X
GK-108D08-012334
SALK_066891.42.70.X
SALK_078349.47.35.X
SALK_036820.54.75.X

AT1G64250

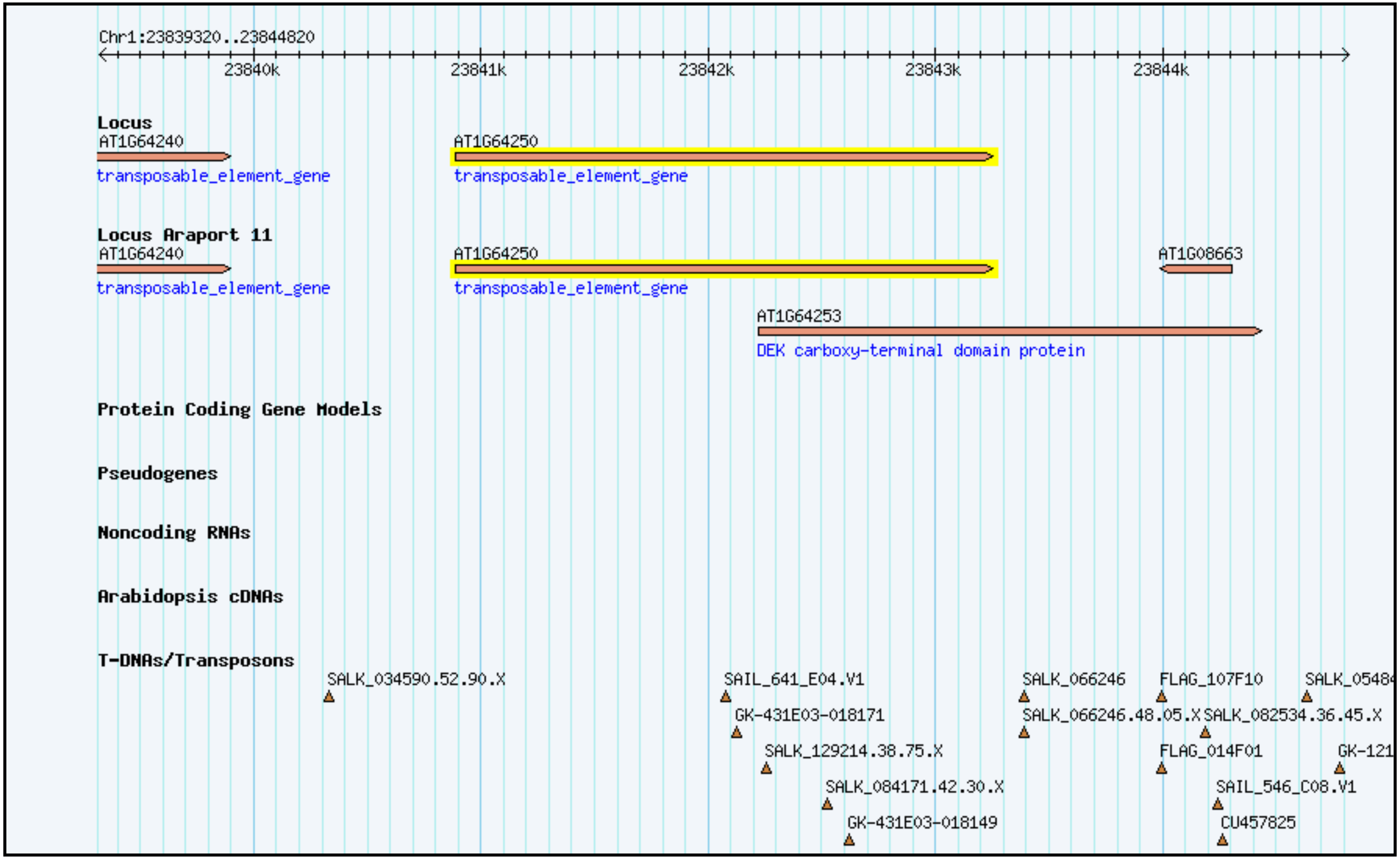
Chr1:23839320..23844820
23840k
23841k
23842k
23843k
23844k
Locus
AT1G64240
transposable_element_gene
AT1G64250
transposable_element_gene
Locus Araport 11
AT1G64240
transposable_element_gene
AT1G64250
transposable_element_gene
AT1G08663
AT1G64253
DEK carboxy-terminal domain protein
Protein Coding Gene Models
Pseudogenes
Noncoding RNAs
Arabidopsis cDNAs
T-DNAs/Transposons
SALK_034590.52.90.X
SAIL_641_E04.V1
GK-431E03-018171
SALK_129214.38.75.X
SALK_084171.42.30.X
GK-431E03-018149
SALK_066246
SALK_066246.48.05.X
FLAG_107F10
FLAG_014F01
SALK_082534.36.45.X
SAIL_546_C08.V1
CU457825

AT1G64255

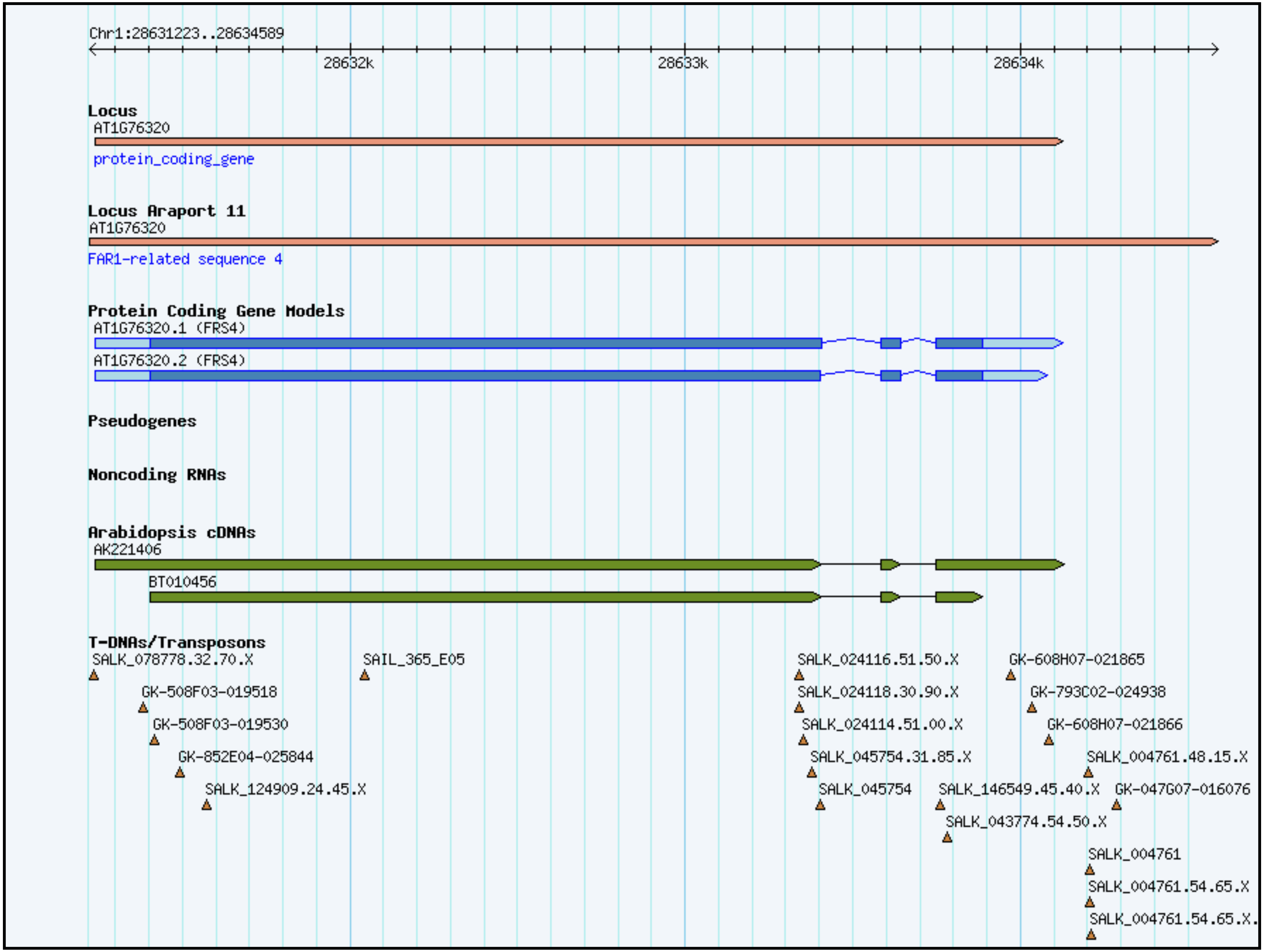

Chr1:28631223..28634589
28632k
28633k
28634k
Locus
AT1G76320
protein_coding_gene
Locus Araport 11
AT1G76320
FAR1-related sequence 4
Protein Coding Gene Models
AT1G76320.1 (FRS4)
AT1G76320.2 (FRS4)
Pseudogenes
Noncoding RNAs
Arabidopsis cDNAs
AK221406
BT010456
T-DNAs/Transposons
SALK_078778.32.70.X
SAIL_365_E05
SALK_024116.51.50.X
GK-608H07-021865
GK-508F03-019518
SALK_024118.30.90.X
GK-793C02-024938
GK-508F03-019530
SALK_024114.51.00.X
GK-608H07-021866
GK-852E04-025844
SALK_045754.31.85.X
SALK_004761.48.15.X
SALK_124909.24.45.X
SALK_045754
SALK_146549.45.40.X
GK-047G07-016076
SALK_043774.54.50.X
SALK_004761
SALK_004761.54.65.X
SALK_004761.54.65.X.

AT1G64260

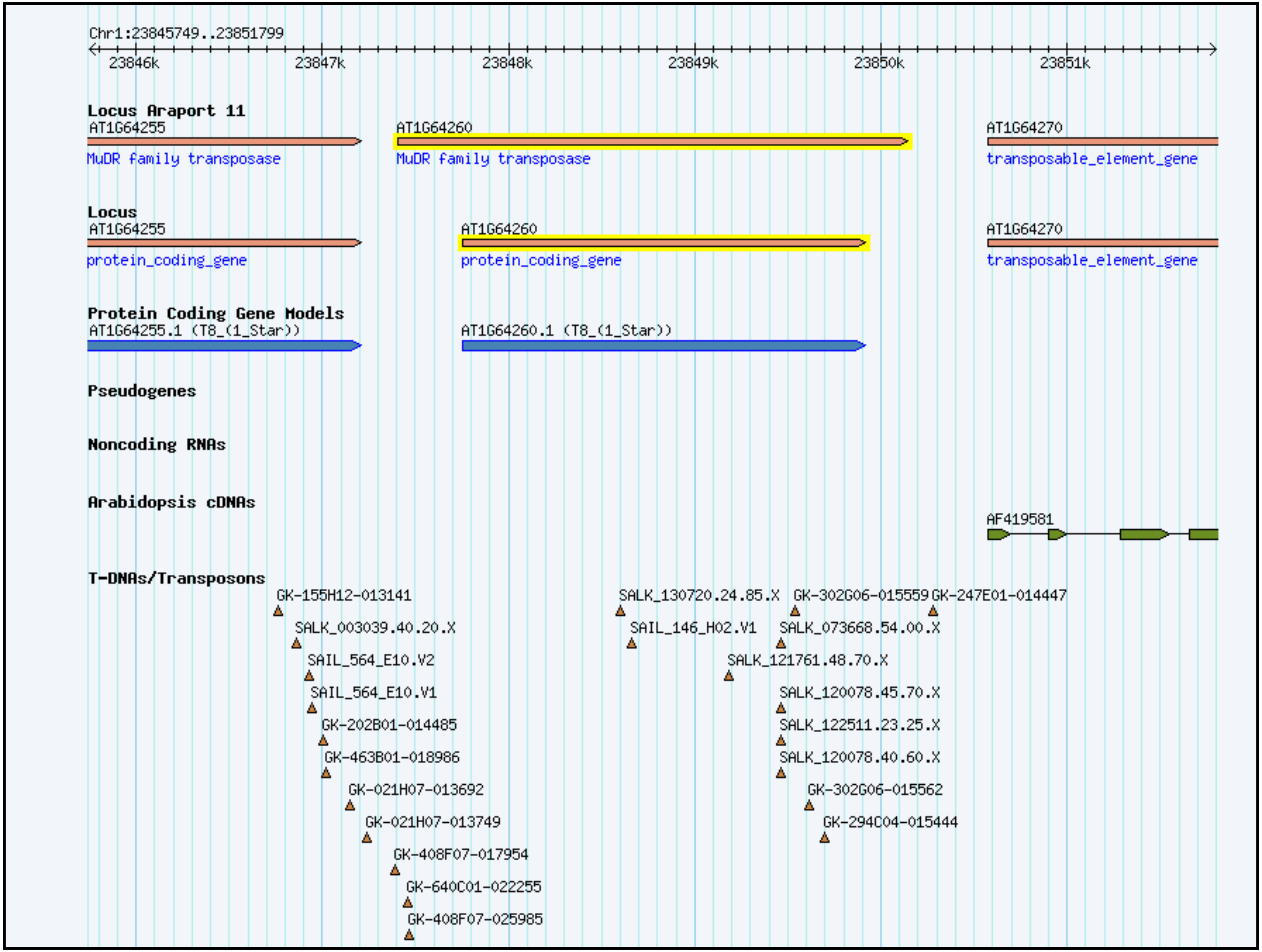

Chr1:23845749..23851799
23846k
23847k
23848k
23849k
23850k
23851k
Locus Araport 11
AT1G64255
MuDR family transposase
AT1G64260
MuDR family transposase
AT1G64270
transposable_element_gene
Locus
AT1G64255
protein_coding_gene
AT1G64260
protein_coding_gene
AT1G64270
transposable_element_gene
Protein Coding Gene Models
AT1G64255.1 (T8_(1_Star))
AT1G64260.1 (T8_(1_Star))
Pseudogenes
Noncoding RNAs
Arabidopsis cDNAs
AF419581
T-DNAs/Transposons
GK-155H12-013141
SALK_003039.40.20.X
SAIL_564_E10.V2
SAIL_564_E10.V1
GK-202B01-014485
GK-463B01-018986
GK-021H07-013692
GK-021H07-013749
GK-408F07-017954
GK-640C01-022255
GK-408F07-025985
SALK_130720.24.85.X
SAIL_146_H02.V1
GK-302G06-015559
GK-247E01-014447
SALK_073668.54.00.X
SALK_121761.48.70.X
SALK_120078.45.70.X
SALK_122511.23.25.X
SALK_120078.40.60.X
GK-302G06-015562
GK-294C04-015444

AT1G64270

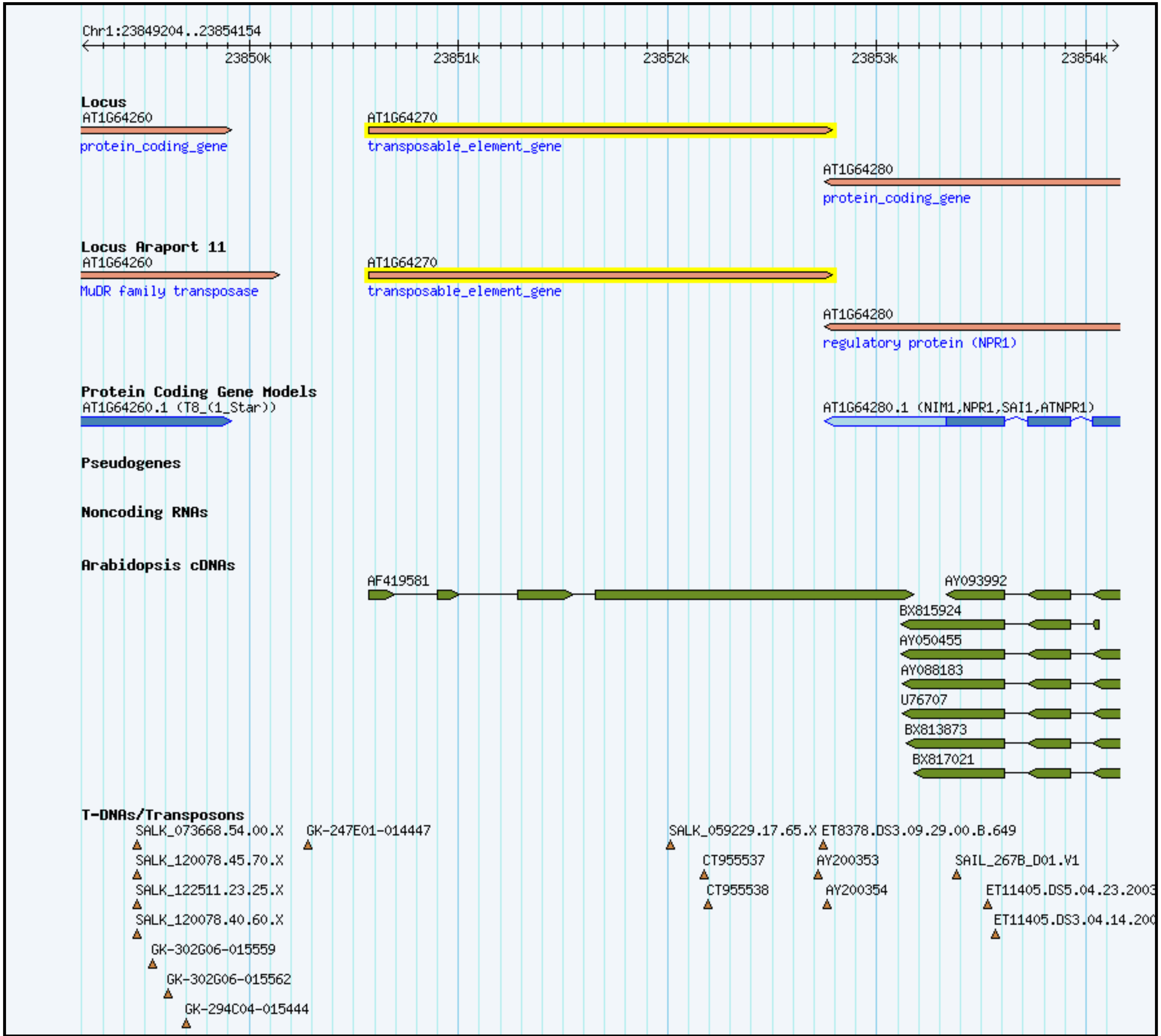

Chr1:23849204..23854154
23850k
23851k
23852k
23853k
23854k
Locus
AT1G64260
protein_coding_gene
AT1G64270
transposable_element_gene
AT1G64280
protein_coding_gene
Locus Araport 11
AT1G64260
MuDR family transposase
AT1G64270
transposable_element_gene
AT1G64280
regulatory protein (NPR1)
Protein Coding Gene Models
AT1G64260.1 (T8_(1_Star))
AT1G64280.1 (NIM1,NPR1,SAI1,ATNPR1)
Pseudogenes
Noncoding RNAs
Arabidopsis cDNAs
AF419581
AY093992
BX815924
AY050455
AY088183
U76707
BX813873
BX817021
T-DNAs/Transposons
SALK_073668.54.00.X
GK-247E01-014447
SALK_059229.17.65.X
ET8378.DS3.09.29.00.B.649
SALK_120078.45.70.X
CT955537
AY200353
SAIL_267B_D01.V1
SALK_122511.23.25.X
CT955538
AY200354
SALK_120078.40.60.X
GK-302G06-015559
GK-302G06-015562
GK-294C04-015444

AT1G69950

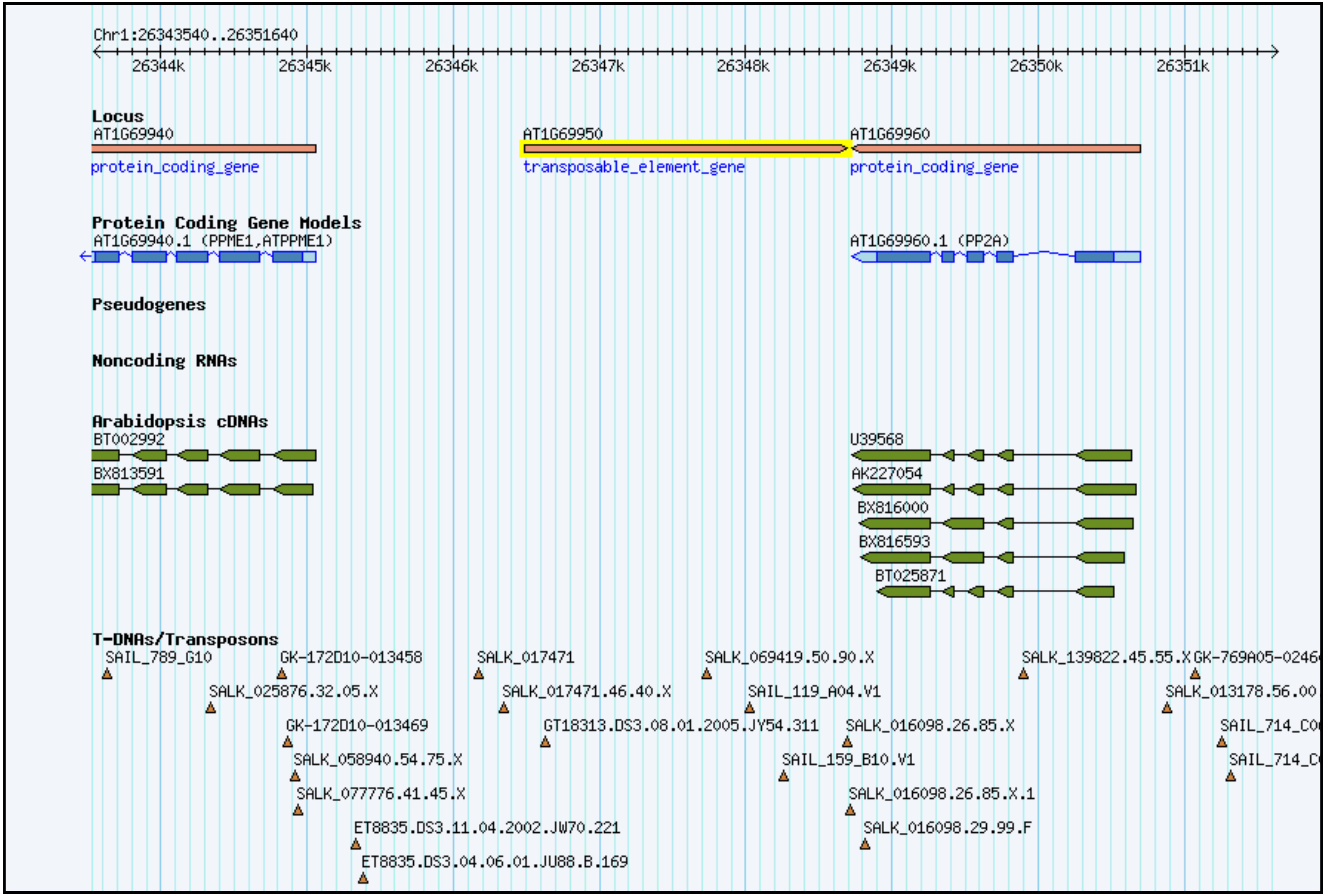
Chr1:26343540..26351640
26344k
26345k
26346k
26347k
26348k
26349k
26350k
26351k
Locus
AT1G69940
protein_coding_gene
AT1G69950
transposable_element_gene
AT1G69960
protein_coding_gene
Protein Coding Gene Models
AT1G69940.1 (PPME1,ATPPME1)
AT1G69960.1 (PP2A)
Pseudogenes
Noncoding RNAs
Arabidopsis cDNAs
BT002992
BX813591
U39568
AK227054
BX816000
BX816593
BT025871
T-DNAs/Transposons
SAIL_789_G10
GK-172D10-013458
SALK_017471
SALK_069419.50.90.X
SALK_139822.45.55.X
SALK_025876.32.05.X
SALK_017471.46.40.X
SAIL_119_A04.V1
GK-172D10-013469
GT18313.DS3.08.01.2005.JY54.311
SALK_016098.26.85.X
SALK_058940.54.75.X
SAIL_159_B10.V1
SALK_077776.41.45.X
SALK_016098.26.85.X.1
ET8835.DS3.11.04.2002.JW70.221
SALK_016098.29.99.F
ET8835.DS3.04.06.01.JU88.B.169

AT1G76320

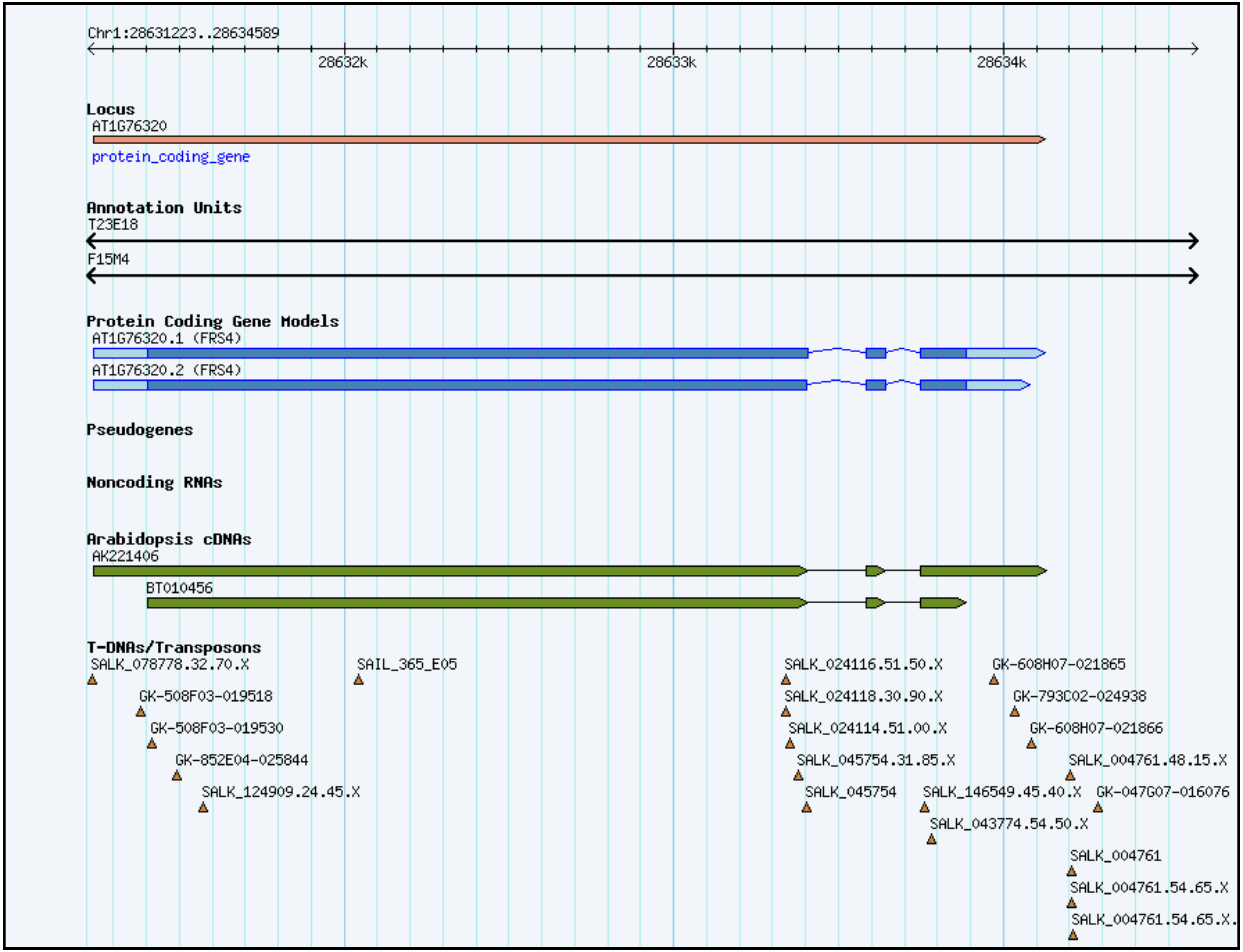

Chr1:28631223..28634589
28632k
28633k
28634k
Locus
AT1G76320
protein_coding_gene
Annotation Units
T23E18
F15M4
Protein Coding Gene Models
AT1G76320.1 (FRS4)
AT1G76320.2 (FRS4)
Pseudogenes
Noncoding RNAs
Arabidopsis cDNAs
AK221406
BT010456
T-DNAs/Transposons
SALK_078778.32.70.X
SAIL_365_E05
SALK_024116.51.50.X
GK-608H07-021865
GK-508F03-019518
SALK_024118.30.90.X
GK-793C02-024938
GK-508F03-019530
SALK_024114.51.00.X
GK-608H07-021866
GK-852E04-025844
SALK_045754.31.85.X
SALK_004761.48.15.X
SALK_124909.24.45.X
SALK_045754
SALK_146549.45.40.X
GK-047G07-016076
SALK_043774.54.50.X
SALK_004761
SALK_004761.54.65.X
SALK_004761.54.65.X.

AT1G79740

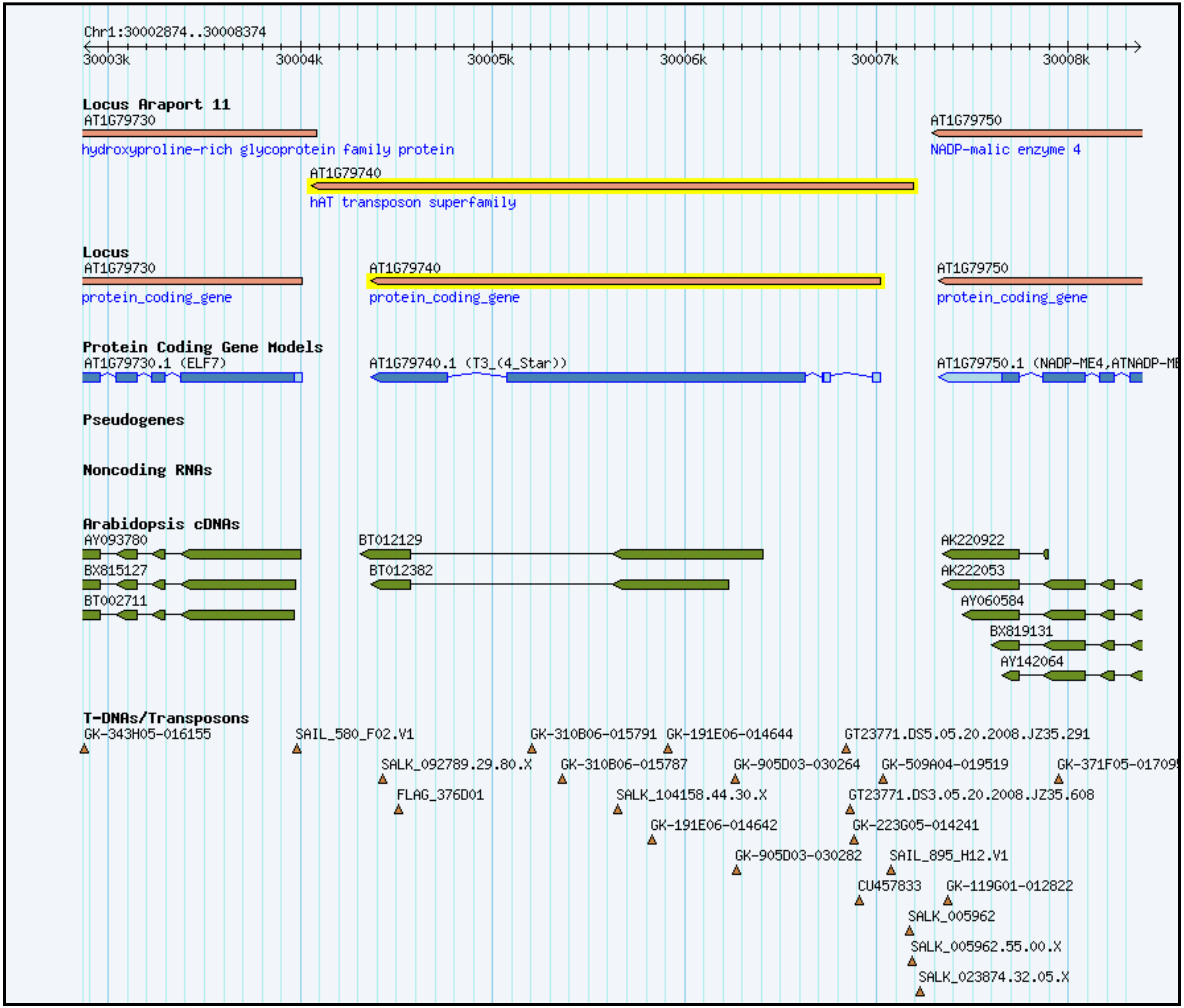
Chr1:30002874..30008374
30003k
30004k
30005k
30006k
30007k
30008k
Locus Araport 11
AT1G79730
hydroxyproline-rich glycoprotein family protein
AT1G79750
NADP-malic enzyme 4
AT1G79740
hAT transposon superfamily
Locus
AT1G79730
protein_coding_gene
AT1G79740
protein_coding_gene
AT1G79750
protein_coding_gene
Protein Coding Gene Models
AT1G79730.1 (ELF7)
AT1G79740.1 (T3_(4_Star))
AT1G79750.1 (NADP-ME4,ATNADP-ME
Pseudogenes
Noncoding RNAs
Arabidopsis cDNAs
AY093780
BX815127
BT002711
BT012129
BT012382
AK220922
AK222053
AY060584
BX819131
AY142064
T-DNAs/Transposons
GK-343H05-016155
SAIL_580_F02.V1
SALK_092789.29.80.X
FLAG_376D01
GK-310B06-015791
GK-310B06-015787
GK-191E06-014644
SALK_104158.44.30.X
GK-191E06-014642
GK-905D03-030264
GK-905D03-030282
GT23771.DS5.05.20.2008.JZ35.291
GT23771.DS3.05.20.2008.JZ35.608
GK-223G05-014241
CU457833
GK-509A04-019519
SAIL_895_H12.V1
GK-119G01-012822
SALK_005962
SALK_005962.55.00.X
SALK_023874.32.05.X
GK-371F05-01709

AT1G80010

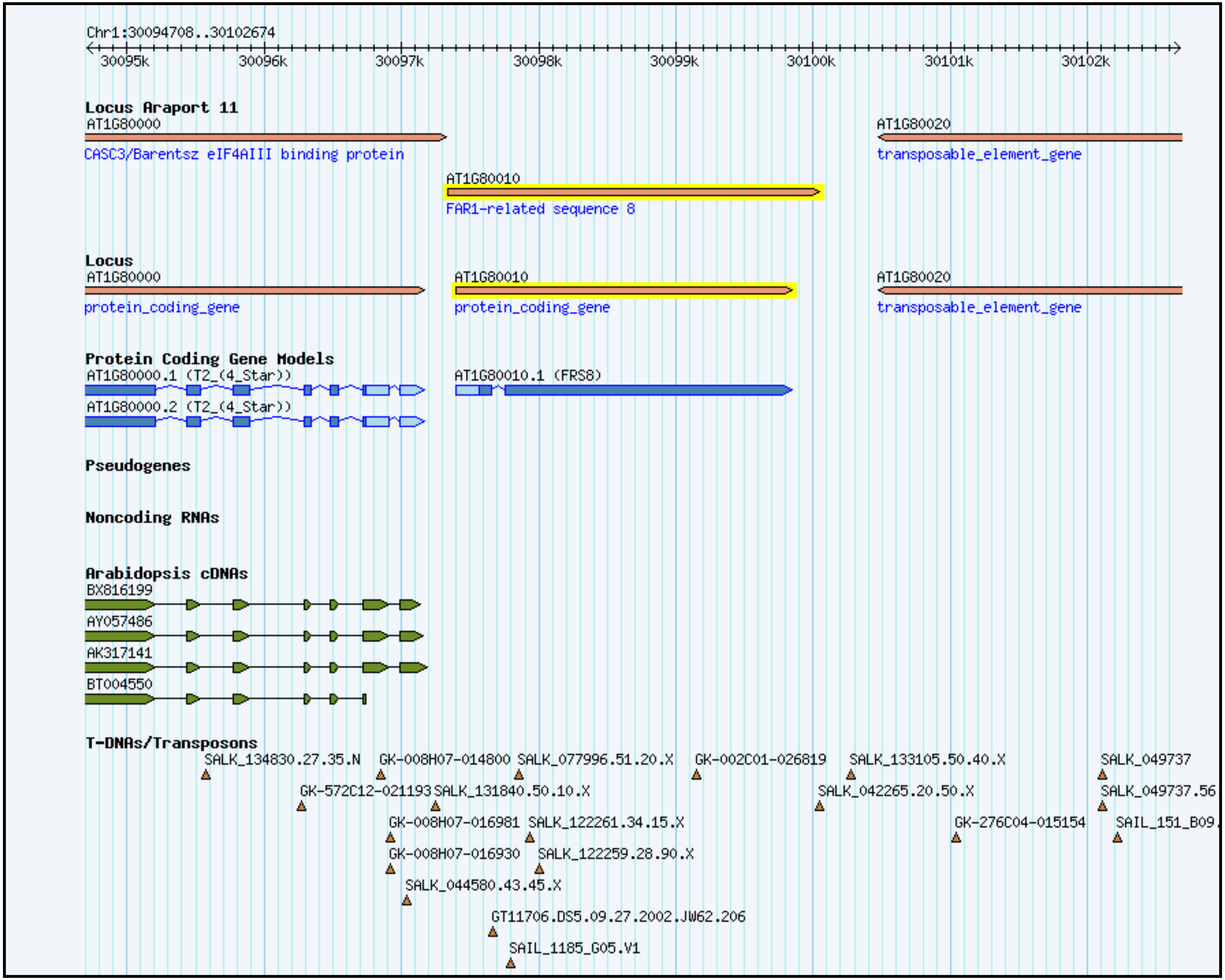

Chr1:30094708..30102674
30095k
30096k
30097k
30098k
30099k
30100k
30101k
30102k
Locus Araport 11
AT1G80000
CASC3/Barentsz eIF4AIII binding protein
AT1G80020
transposable_element_gene
AT1G80010
FAR1-related sequence 8
Locus
AT1G80000
protein_coding_gene
AT1G80010
protein_coding_gene
AT1G80020
transposable_element_gene
Protein Coding Gene Models
AT1G80000.1 (T2_(4_Star))
AT1G80010.1 (FRS8)
AT1G80000.2 (T2_(4_Star))
Pseudogenes
Noncoding RNAs
Arabidopsis cDNAs
BX816199
AY057486
AK317141
BT004550
T-DNAs/Transposons
SALK_134830.27.35.N
GK-008H07-014800
SALK_077996.51.20.X
GK-002C01-026819
SALK_133105.50.40.X
SALK_049737
GK-572C12-021193
SALK_131840.50.10.X
SALK_042265.20.50.X
SALK_049737.56
GK-008H07-016981
SALK_122261.34.15.X
GK-276C04-015154
SAIL_151_B09.
GK-008H07-016930
SALK_122259.28.90.X
SALK_044580.43.45.X
GT11706.DS5.09.27.2002.JW62.206
SAIL_1185_G05.V1

AT1G80020

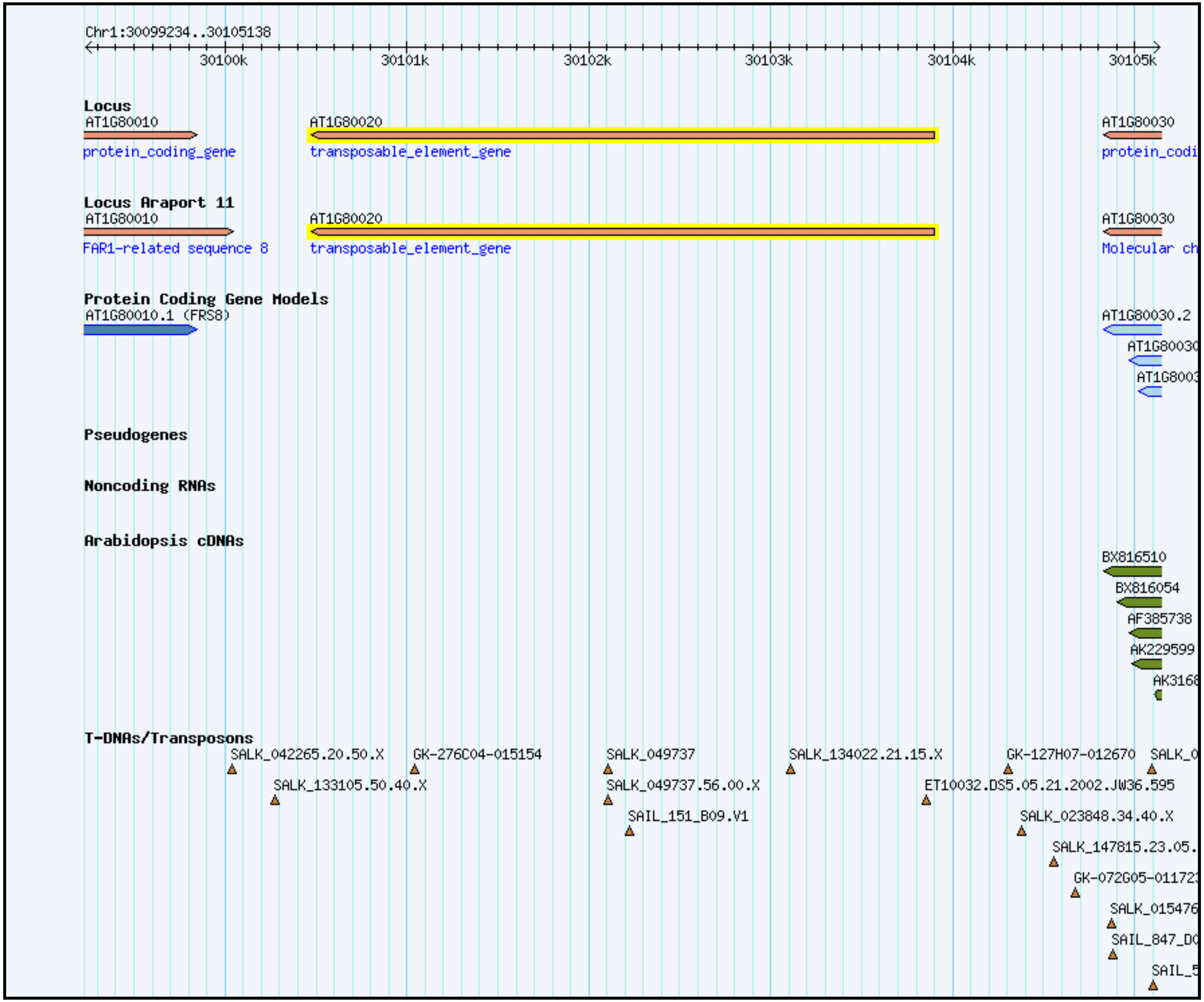

Chr1:30099234..30105138
30100k
30101k
30102k
30103k
30104k
30105k
Locus
AT1G80010
protein_coding_gene
AT1G80020
transposable_element_gene
AT1G80030
Locus Araport 11
AT1G80010
FAR1-related sequence 8
AT1G80020
transposable_element_gene
AT1G80030
Protein Coding Gene Models
AT1G80010.1 (FRS8)
AT1G80030.2
Pseudogenes
Noncoding RNAs
Arabidopsis cDNAs
BX816510
BX816054
AF385738
AK229599
T-DNAs/Transposons
SALK_042265.20.50.X
GK-276C04-015154
SALK_049737
SALK_134022.21.15.X
GK-127H07-012670
SALK_133105.50.40.X
SALK_049737.56.00.X
ET10032.DS5.05.21.2002.JW36.595
SAIL_151_B09.V1
SALK_023848.34.40.X

AT2G27110

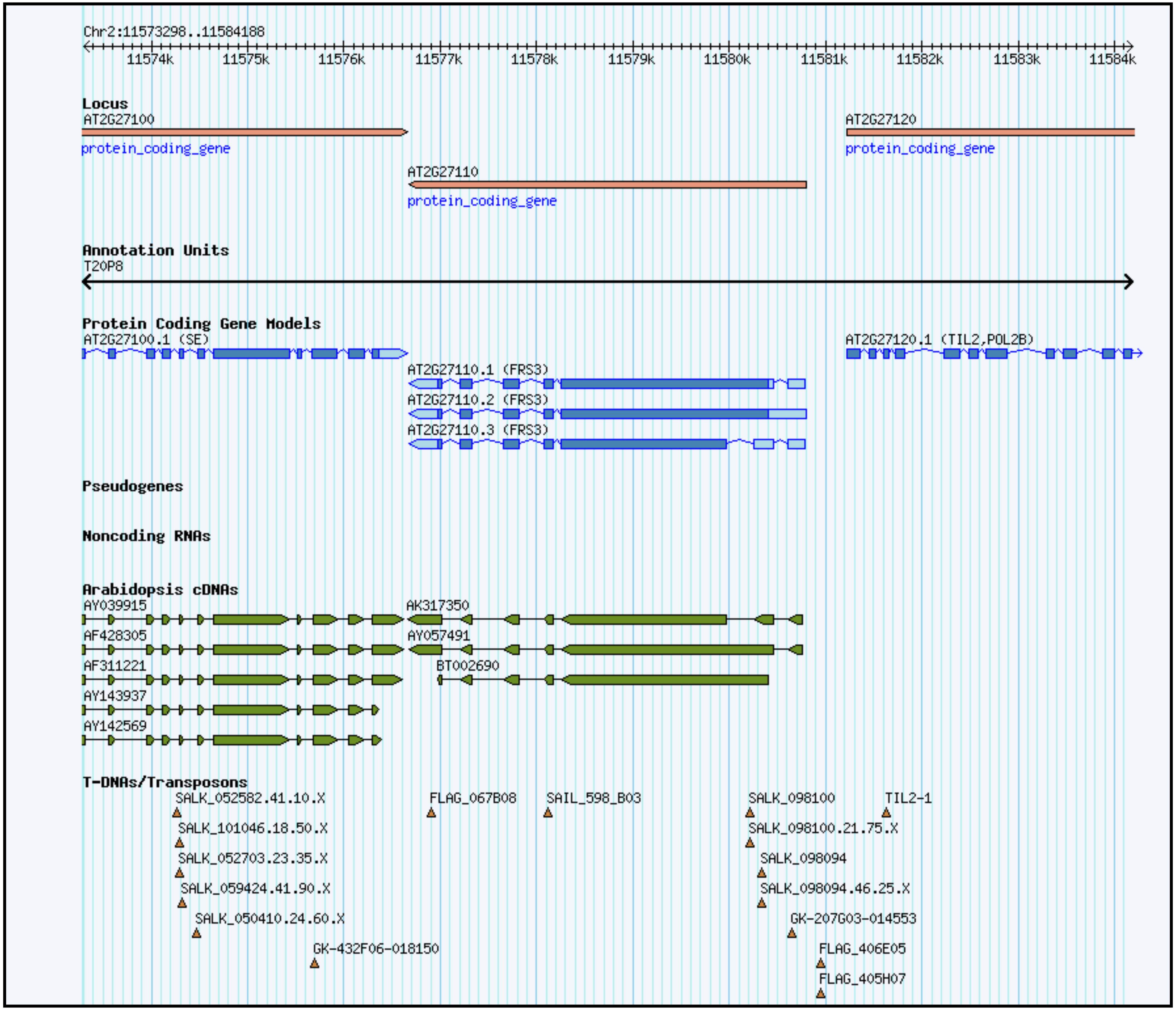
Chr2:11573298..11584188
11574k
11575k
11576k
11577k
11578k
11579k
11580k
11581k
11582k
11583k
11584k
Locus
AT2G27100
protein_coding_gene
AT2G27120
protein_coding_gene
AT2G27110
protein_coding_gene
Annotation Units
T20P8
Protein Coding Gene Models
AT2G27100.1 (SE)
AT2G27120.1 (TIL2,POL2B)
AT2G27110.1 (FRS3)
AT2G27110.2 (FRS3)
AT2G27110.3 (FRS3)
Pseudogenes
Noncoding RNAs
Arabidopsis cDNAs
AY039915
AK317350
AF428305
AY057491
AF311221
BT002690
AY143937
AY142569
T-DNAs/Transposons
SALK_052582.41.10.X
FLAG_067B08
SAIL_598_B03
SALK_098100
TIL2-1
SALK_101046.18.50.X
SALK_098100.21.75.X
SALK_052703.23.35.X
SALK_098094
SALK_059424.41.90.X
SALK_098094.46.25.X
SALK_050410.24.60.X
GK-207G03-014553
GK-432F06-018150
FLAG_406E05
FLAG_405H07

AT2G30640

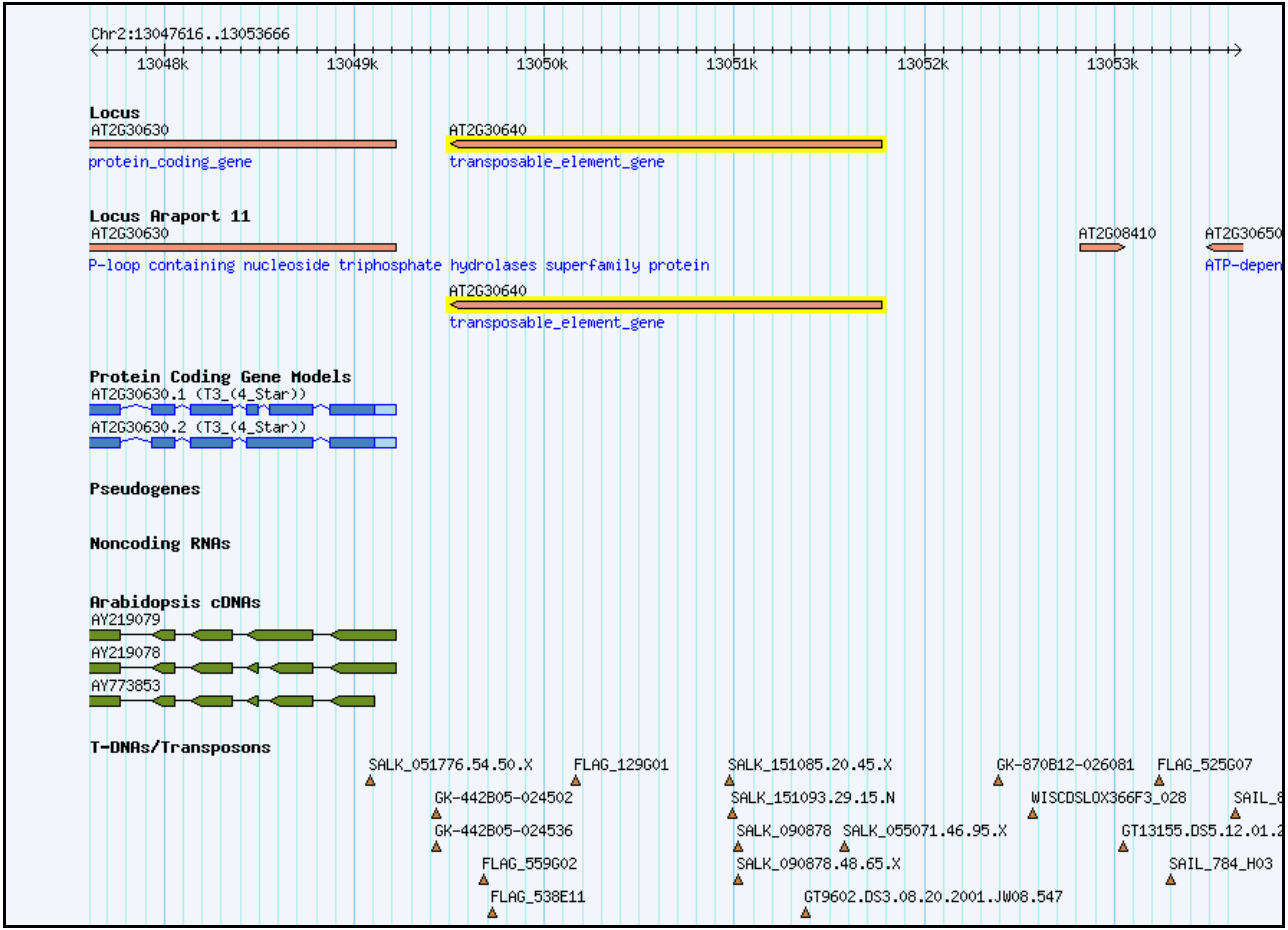

Chr2:13047616..13053666
13048k
13049k
13050k
13051k
13052k
13053k
Locus
AT2G30630
protein_coding_gene
AT2G30640
transposable_element_gene
Locus Araport 11
AT2G30630
P-loop containing nucleoside triphosphate hydrolases superfamily protein
AT2G08410
AT2G30650
ATP-depen
AT2G30640
transposable_element_gene
Protein Coding Gene Models
AT2G30630.1 (T3_(4_Star))
AT2G30630.2 (T3_(4_Star))
Pseudogenes
Noncoding RNAs
Arabidopsis cDNAs
AY219079
AY219078
AY773853
T-DNAs/Transposons
SALK_051776.54.50.X
FLAG_129G01
SALK_151085.20.45.X
GK-870B12-026081
FLAG_525G07
GK-442B05-024502
SALK_151093.29.15.N
WISCDSLOX366F3_028
GK-442B05-024536
SALK_090878
SALK_055071.46.95.X
FLAG_559G02
SALK_090878.48.65.X
SAIL_784_H03
FLAG_538E11
GT9602.DS3.08.20.2001.JW08.547

AT2G32250

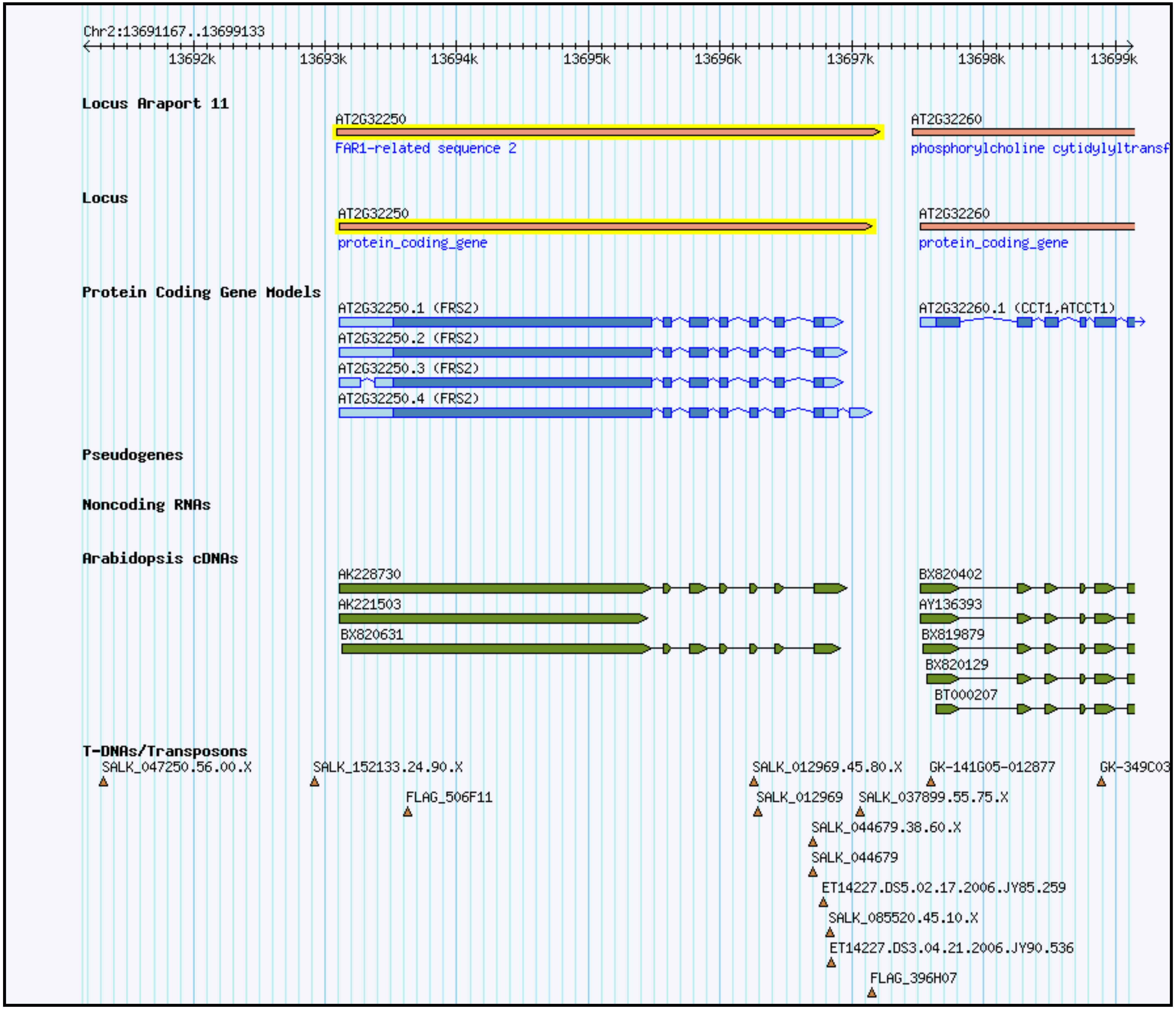

Chr2:13691167..13699133
13692k
13693k
13694k
13695k
13696k
13697k
13698k
13699k
Locus Araport 11
AT2G32250
FAR1-related sequence 2
AT2G32260
phosphorylcholine cytidylyltransf
Locus
AT2G32250
protein_coding_gene
AT2G32260
protein_coding_gene
Protein Coding Gene Models
AT2G32250.1 (FRS2)
AT2G32250.2 (FRS2)
AT2G32250.3 (FRS2)
AT2G32250.4 (FRS2)
AT2G32260.1 (CCT1,ATCCT1)
Pseudogenes
Noncoding RNAs
Arabidopsis cDNAs
AK228730
AK221503
BX820631
BX820402
AY136393
BX819879
BX820129
BT000207
T-DNAs/Transposons
SALK_047250.56.00.X
SALK_152133.24.90.X
FLAG_506F11
SALK_012969.45.80.X
SALK_012969
GK-141G05-012877
SALK_037899.55.75.X
GK-349C03
SALK_044679.38.60.X
SALK_044679
ET14227.DS5.02.17.2006.JY85.259
SALK_085520.45.10.X
ET14227.DS3.04.21.2006.JY90.536
FLAG_396H07

AT3G04605

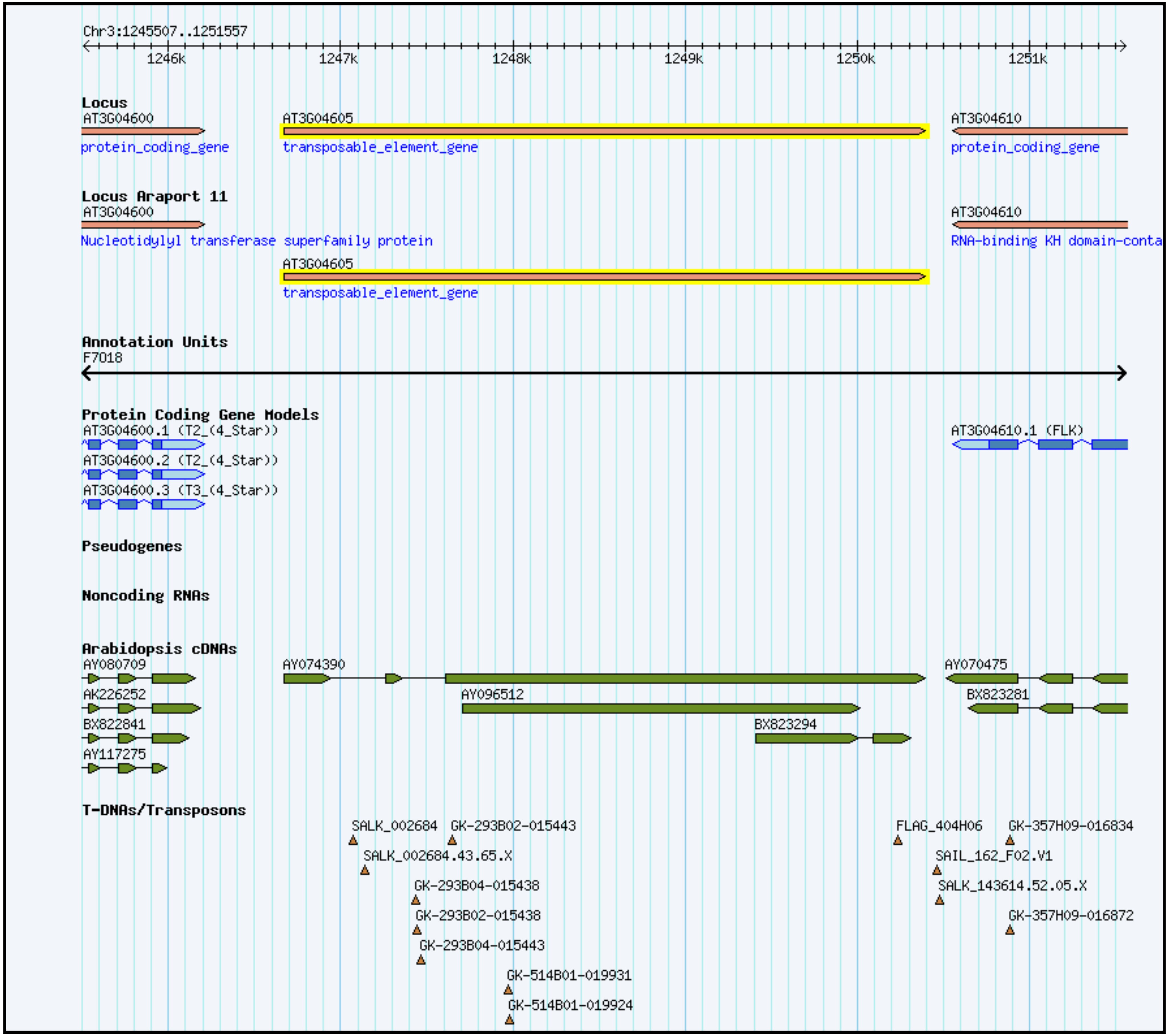
Chr3:1245507..1251557
1246k
1247k
1248k
1249k
1250k
1251k
Locus
AT3G04600
protein_coding_gene
AT3G04605
transposable_element_gene
AT3G04610
protein_coding_gene
Locus Araport 11
AT3G04600
Nucleotidylyl transferase superfamily protein
AT3G04610
RNA-binding KH domain-conta
AT3G04605
transposable_element_gene
Annotation Units
F7O18
Protein Coding Gene Models
AT3G04600.1 (T2_(4_Star))
AT3G04600.2 (T2_(4_Star))
AT3G04600.3 (T3_(4_Star))
AT3G04610.1 (FLK)
Pseudogenes
Noncoding RNAs
Arabidopsis cDNAs
AY080709
AK226252
BX822841
AY117275
AY074390
AY096512
BX823294
AY070475
BX823281
T-DNAs/Transposons
SALK_002684
GK-293B02-015443
SALK_002684.43.65.X
GK-293B04-015438
GK-293B02-015438
GK-293B04-015443
GK-514B01-019931
GK-514B01-019924
FLAG_404H06
GK-357H09-016834
SAIL_162_F02.V1
SALK_143614.52.05.X
GK-357H09-016872

AT3G05850

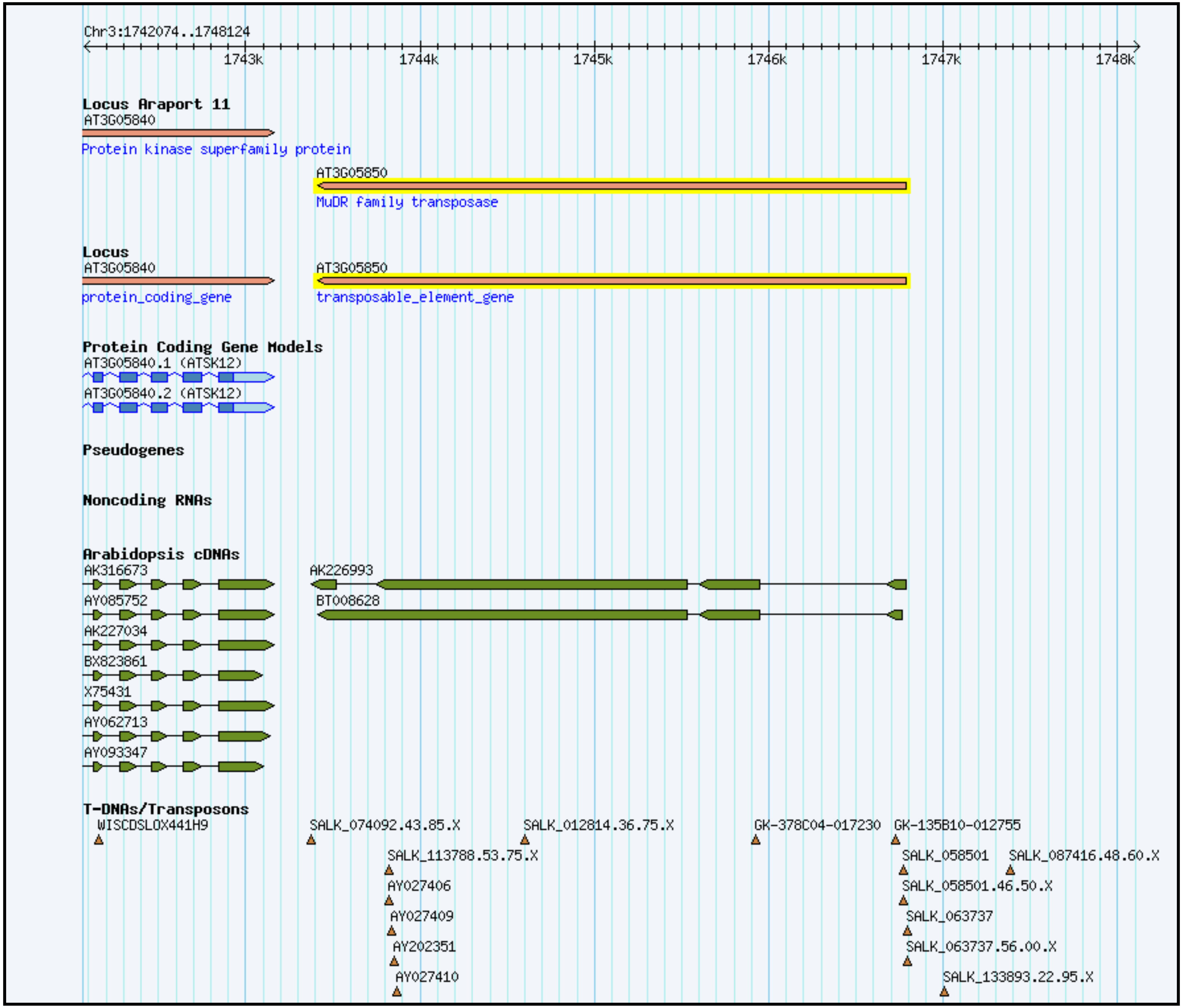
Chr3:1742074..1748124
1743k
1744k
1745k
1746k
1747k
1748k
Locus Araport 11
AT3G05840
Protein kinase superfamily protein
AT3G05850
MuDR family transposase
Locus
AT3G05840
protein_coding_gene
AT3G05850
transposable_element_gene
Protein Coding Gene Models
AT3G05840.1 (ATSK12)
AT3G05840.2 (ATSK12)
Pseudogenes
Noncoding RNAs
Arabidopsis cDNAs
AK316673
AY085752
AK227034
BX823861
X75431
AY062713
AY093347
AK226993
BT008628
T-DNAs/Transposons
WISCDSLOX441H9
SALK_074092.43.85.X
SALK_012814.36.75.X
GK-378C04-017230
GK-135B10-012755
SALK_113788.53.75.X
AY027406
AY027409
AY202351
AY027410
SALK_058501
SALK_087416.48.60.X
SALK_058501.46.50.X
SALK_063737
SALK_063737.56.00.X
SALK_133893.22.95.X

AT3G06250

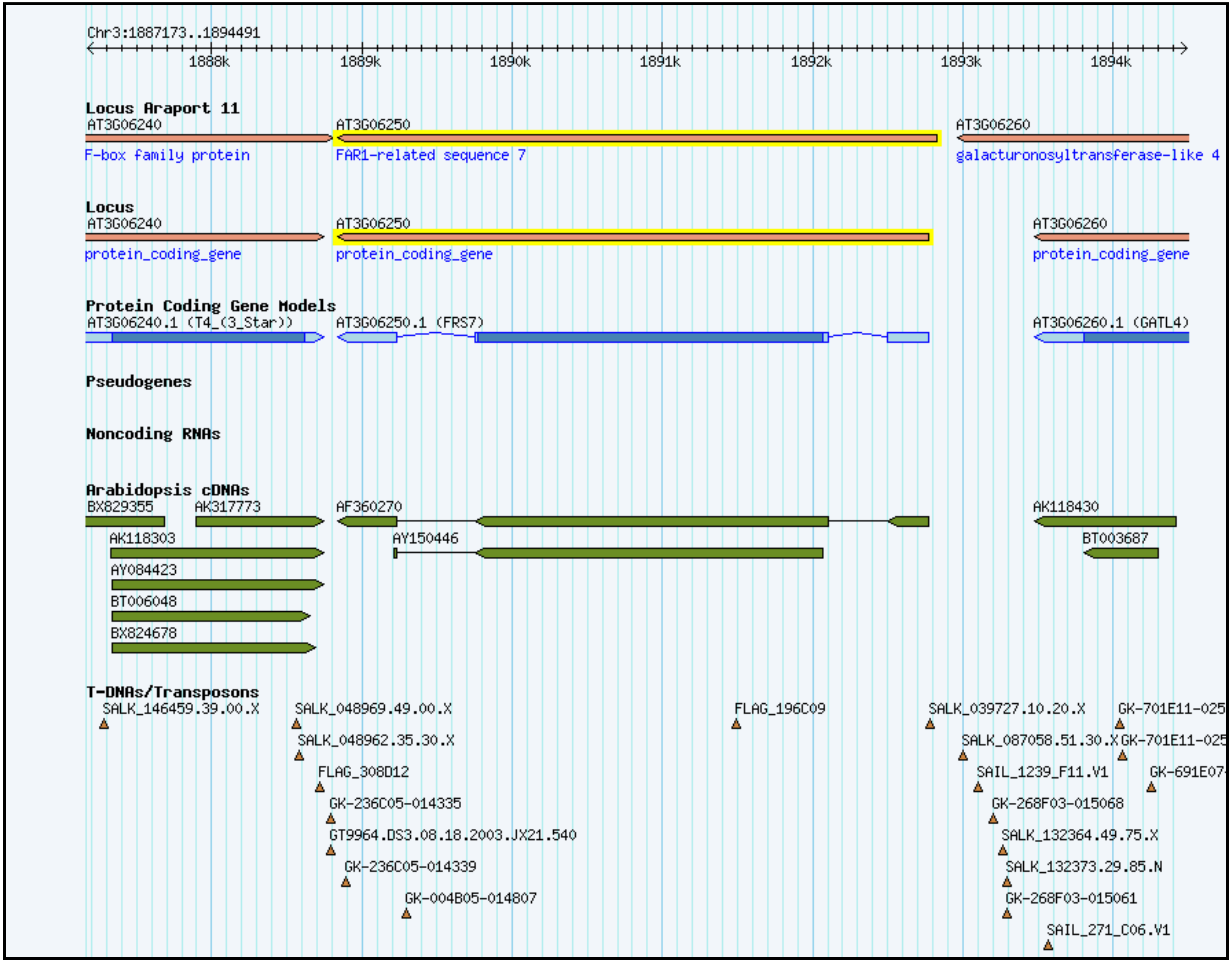

Chr3:1887173..1894491
1888k
1889k
1890k
1891k
1892k
1893k
1894k
Locus Araport 11
AT3G06240
F-box family protein
AT3G06250
FAR1-related sequence 7
AT3G06260
galacturonosyltransferase-like 4
Locus
AT3G06240
protein_coding_gene
AT3G06250
protein_coding_gene
AT3G06260
protein_coding_gene
Protein Coding Gene Models
AT3G06240.1 (T4_(3_Star))
AT3G06250.1 (FRS7)
AT3G06260.1 (GATL4)
Pseudogenes
Noncoding RNAs
Arabidopsis cDNAs
BX829355
AK317773
AF360270
AK118430
AK118303
AY150446
BT003687
AY084423
BT006048
BX824678
T-DNAs/Transposons
SALK_146459.39.00.X
SALK_048969.49.00.X
FLAG_196C09
SALK_039727.10.20.X
SALK_048962.35.30.X
SALK_087058.51.30.X
FLAG_308D12
SAIL_1239_F11.V1
GK-236C05-014335
GK-268F03-015068
GT9964.DS3.08.18.2003.JX21.540
SALK_132364.49.75.X
GK-236C05-014339
SALK_132373.29.85.N
GK-004B05-014807
GK-268F03-015061
SAIL_271_C06.V1

AT3G06940

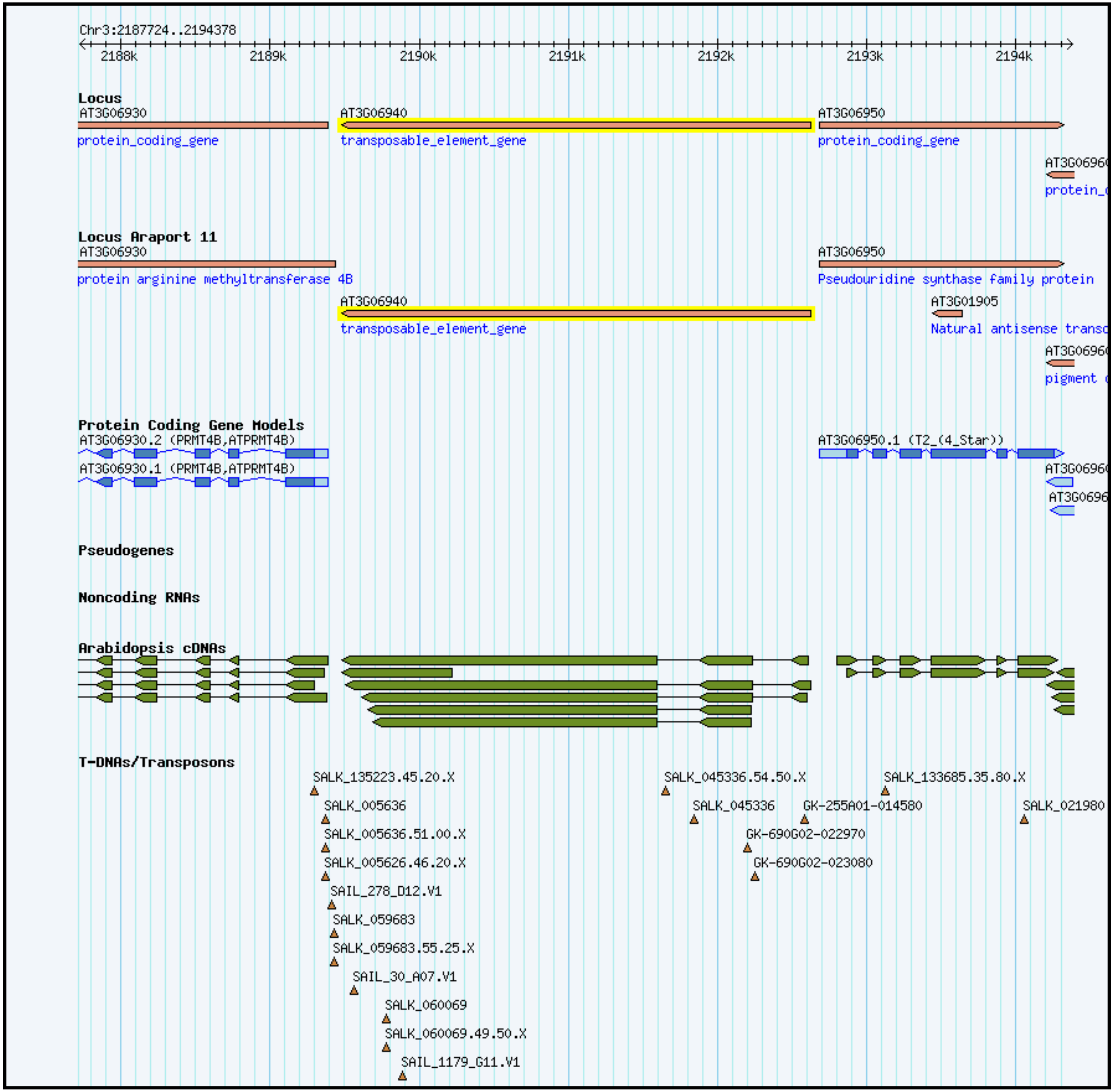

Chr3:2187724..2194378
2188k
2189k
2190k
2191k
2192k
2193k
2194k
Locus
AT3G06930
protein_coding_gene
AT3G06940
transposable_element_gene
AT3G06950
protein_coding_gene
Locus Araport 11
AT3G06930
protein arginine methyltransferase 4B
AT3G06940
transposable_element_gene
AT3G06950
Pseudouridine synthase family protein
AT3G01905
Protein Coding Gene Models
AT3G06930.2 (PRMT4B,ATPRMT4B)
AT3G06930.1 (PRMT4B,ATPRMT4B)
AT3G06950.1 (T2_(4_Star))
Pseudogenes
Noncoding RNAs
Arabidopsis cDNAs
T-DNAs/Transposons
SALK_135223.45.20.X
SALK_005636
SALK_005636.51.00.X
SALK_005626.46.20.X
SAIL_278_D12.V1
SALK_059683
SALK_059683.55.25.X
SAIL_30_A07.V1
SALK_060069
SALK_060069.49.50.X
SAIL_1179_G11.V1
SALK_045336.54.50.X
SALK_045336
GK-690G02-022970
GK-690G02-023080
GK-255A01-014580
SALK_133685.35.80.X
SALK_021980

AT3G13010

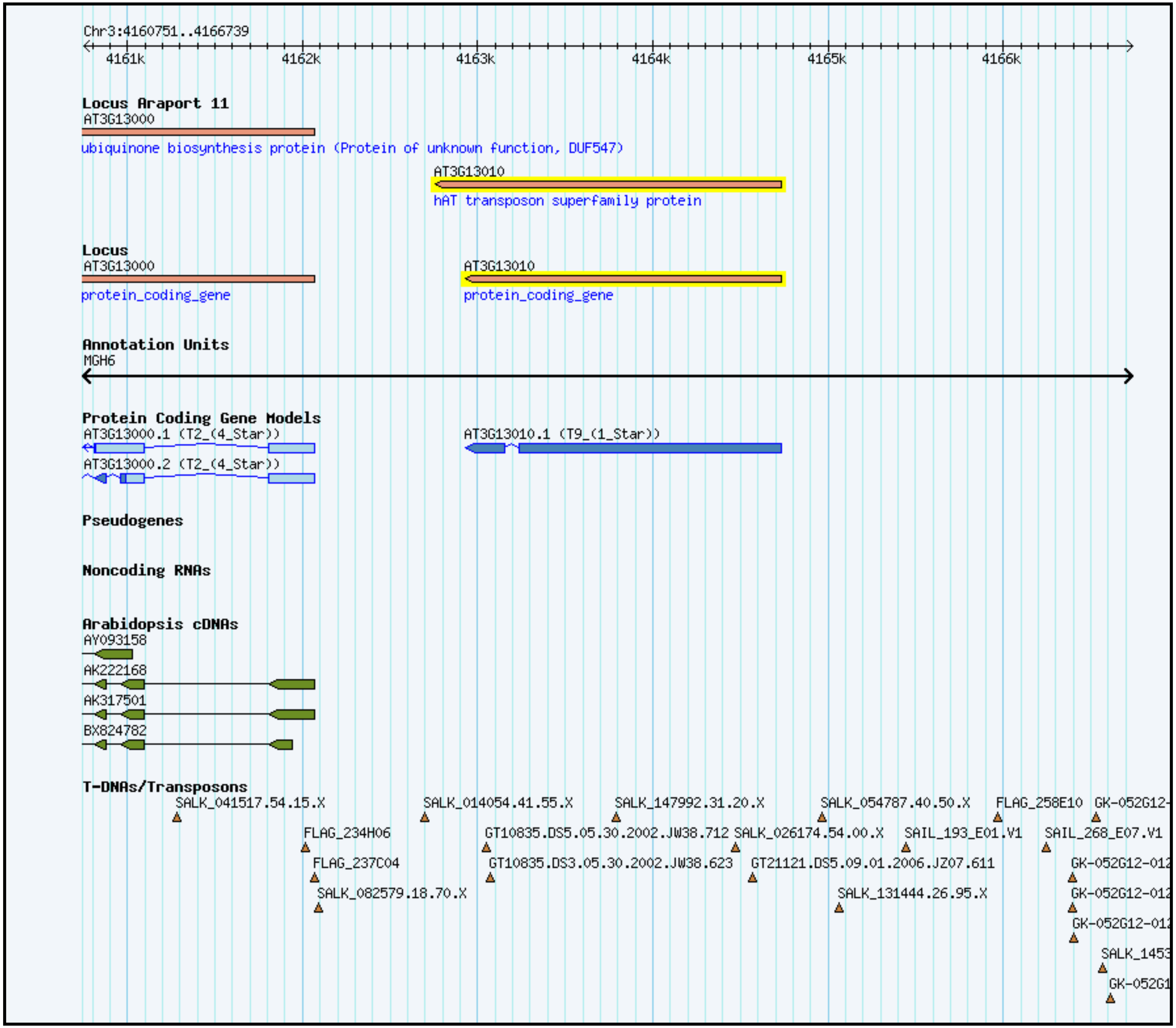
Chr3:4160751..4166739
4161k
4162k
4163k
4164k
4165k
4166k
Locus Araport 11
AT3G13000
ubiquinone biosynthesis protein (Protein of unknown function, DUF547)
AT3G13010
hAT transposon superfamily protein
Locus
AT3G13000
protein_coding_gene
AT3G13010
protein_coding_gene
Annotation Units
MGH6
Protein Coding Gene Models
AT3G13000.1 (T2_(4_Star))
AT3G13000.2 (T2_(4_Star))
AT3G13010.1 (T9_(1_Star))
Pseudogenes
Noncoding RNAs
Arabidopsis cDNAs
AY093158
AK222168
AK317501
BX824782
T-DNAs/Transposons
SALK_041517.54.15.X
SALK_014054.41.55.X
SALK_147992.31.20.X
SALK_054787.40.50.X
FLAG_258E10
FLAG_234H06
GT10835.DS5.05.30.2002.JW38.712
SALK_026174.54.00.X
SAIL_193_E01.V1
SAIL_268_E07.V1
FLAG_237C04
GT10835.DS3.05.30.2002.JW38.623
GT21121.DS5.09.01.2006.JZ07.611
SALK_082579.18.70.X
SALK_131444.26.95.X

AT3G13030

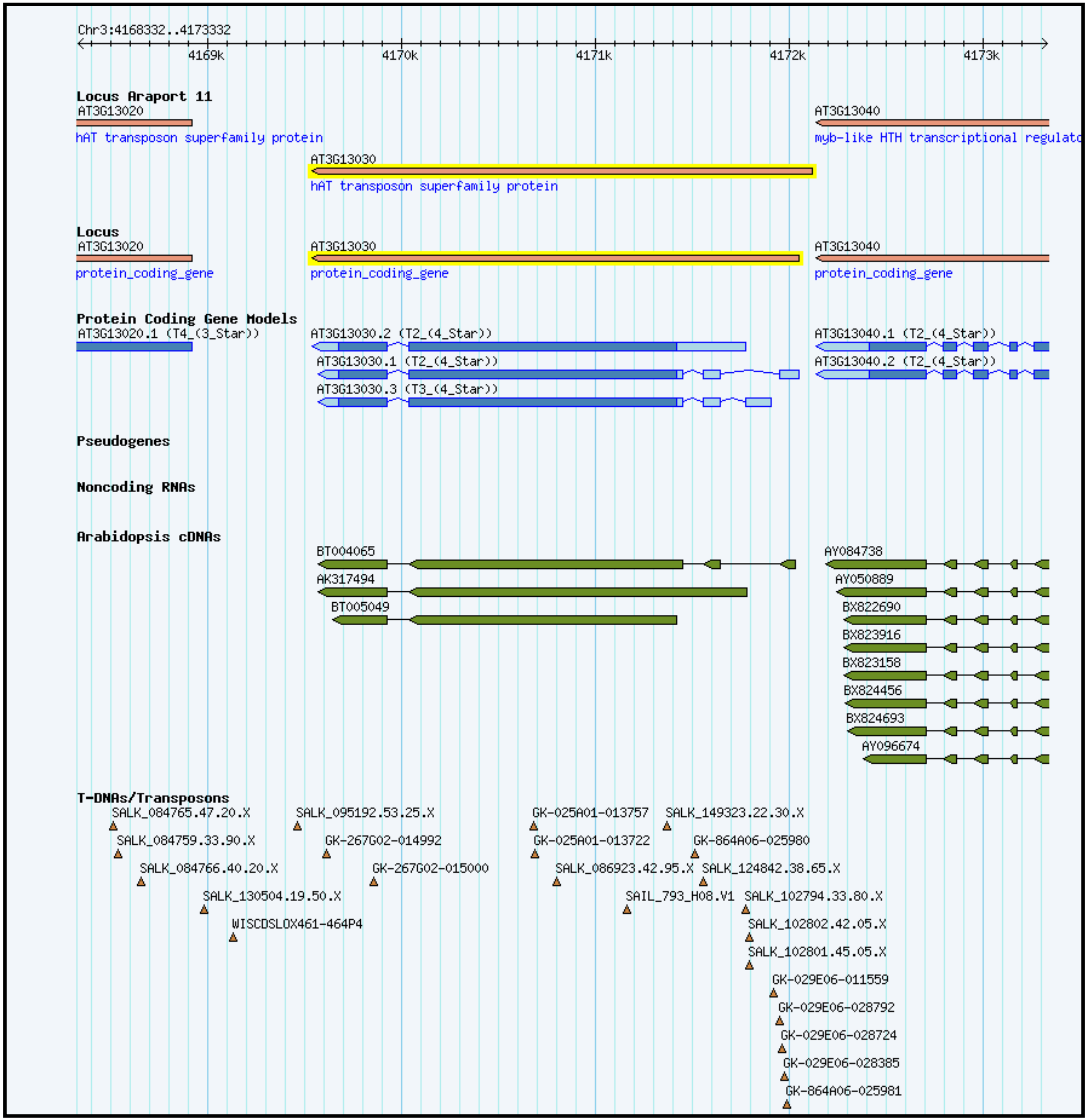

Chr3:4168332..4173332
4169k
4170k
4171k
4172k
4173k
Locus Araport 11
AT3G13020
hAT transposon superfamily protein
AT3G13040
myb-like HTH transcriptional regulato
AT3G13030
hAT transposon superfamily protein
Locus
AT3G13020
protein_coding_gene
AT3G13030
protein_coding_gene
AT3G13040
protein_coding_gene
Protein Coding Gene Models
AT3G13020.1 (T4_(3_Star))
AT3G13030.2 (T2_(4_Star))
AT3G13030.1 (T2_(4_Star))
AT3G13030.3 (T3_(4_Star))
AT3G13040.1 (T2_(4_Star))
AT3G13040.2 (T2_(4_Star))
Pseudogenes
Noncoding RNAs
Arabidopsis cDNAs
BT004065
AK317494
BT005049
AY084738
AY050889
BX822690
BX823916
BX823158
BX824456
BX824693
AY096674
T-DNAs/Transposons
SALK_084765.47.20.X
SALK_084759.33.90.X
SALK_084766.40.20.X
SALK_130504.19.50.X
WISCDSLOX461-464P4
SALK_095192.53.25.X
GK-267G02-014992
GK-267G02-015000
GK-025A01-013757
GK-025A01-013722
SALK_086923.42.95.X
SAIL_793_H08.V1
SALK_149323.22.30.X
GK-864A06-025980
SALK_124842.38.65.X
SALK_102794.33.80.X
SALK_102802.42.05.X
SALK_102801.45.05.X
GK-029E06-011559
GK-029E06-028792
GK-029E06-028724
GK-029E06-028385
GK-864A06-025981

AT3G17290

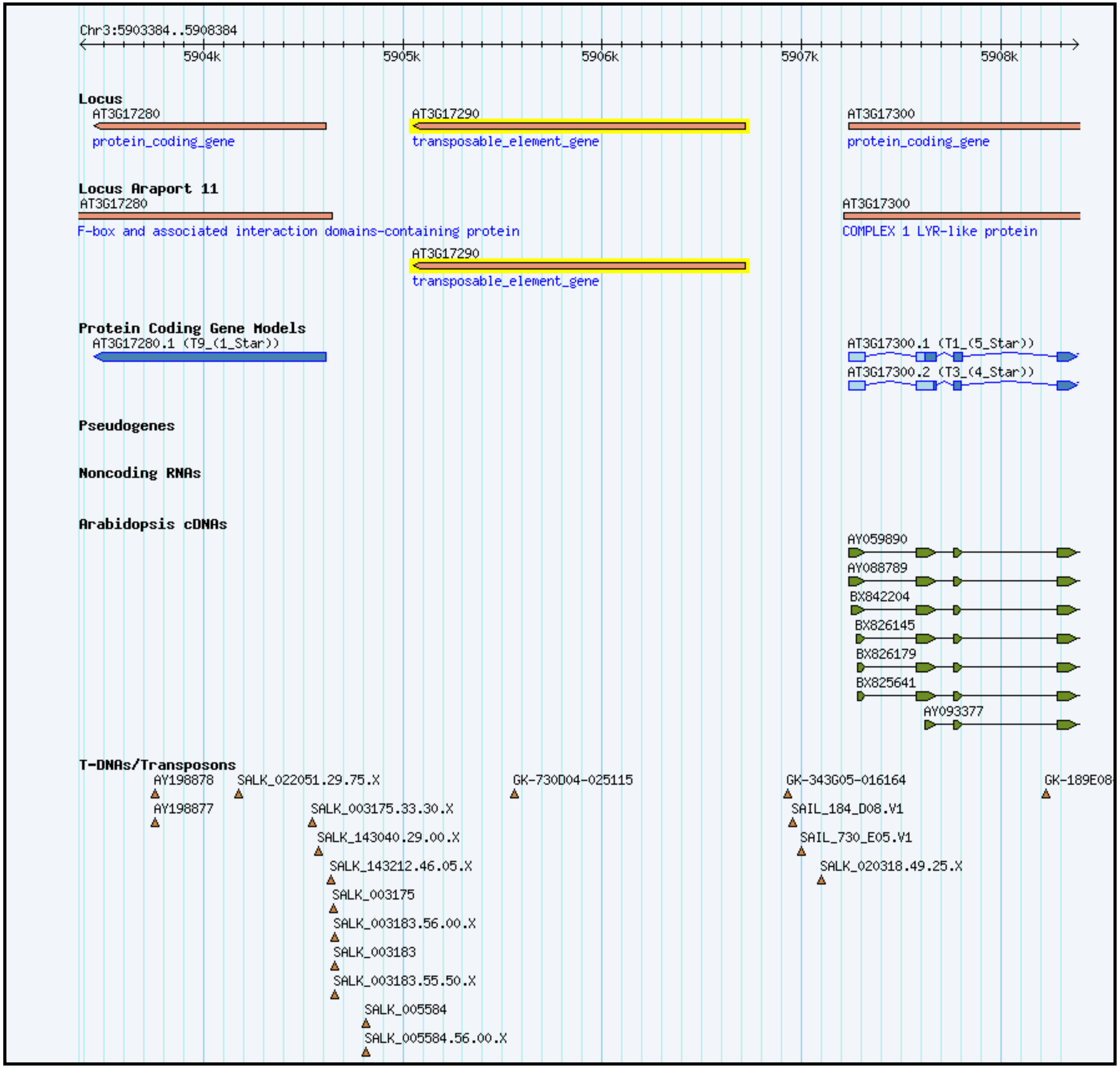

Chr3:5903384..5908384
5904k
5905k
5906k
5907k
5908k
Locus
AT3G17280
protein_coding_gene
AT3G17290
transposable_element_gene
AT3G17300
protein_coding_gene
Locus Araport 11
AT3G17280
F-box and associated interaction domains-containing protein
AT3G17300
COMPLEX 1 LYR-like protein
AT3G17290
transposable_element_gene
Protein Coding Gene Models
AT3G17280.1 (T9_(1_Star))
AT3G17300.1 (T1_(5_Star))
AT3G17300.2 (T3_(4_Star))
Pseudogenes
Noncoding RNAs
Arabidopsis cDNAs
AY059890
AY088789
BX842204
BX826145
BX826179
BX825641
AY093377
T-DNAs/Transposons
AY198878
SALK_022051.29.75.X
GK-730D04-025115
GK-343G05-016164
AY198877
SALK_003175.33.30.X
SAIL_184_D08.V1
SALK_143040.29.00.X
SAIL_730_E05.V1
SALK_143212.46.05.X
SALK_020318.49.25.X
SALK_003175
SALK_003183.56.00.X
SALK_003183
SALK_003183.55.50.X
SALK_005584
SALK_005584.56.00.X

AT3G17450

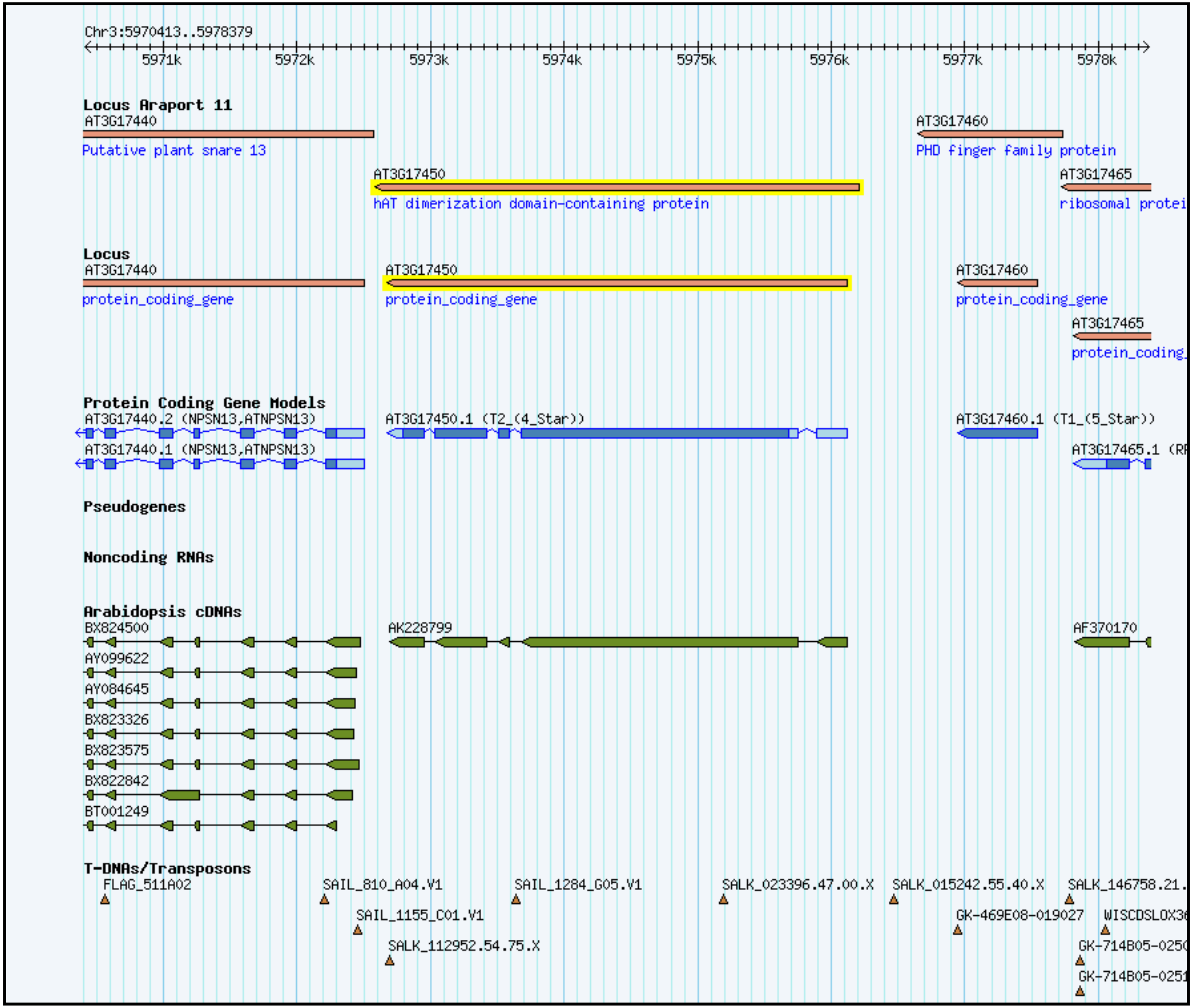
Chr3:5970413..5978379
5971k
5972k
5973k
5974k
5975k
5976k
5977k
5978k
Locus Araport 11
AT3G17440
Putative plant snare 13
AT3G17460
PHD finger family protein
AT3G17450
hAT dimerization domain-containing protein
AT3G17465
ribosomal protei
Locus
AT3G17440
protein_coding_gene
AT3G17450
protein_coding_gene
AT3G17460
protein_coding_gene
AT3G17465
protein_coding
Protein Coding Gene Models
AT3G17440.2 (NPSN13,ATNPSN13)
AT3G17440.1 (NPSN13,ATNPSN13)
AT3G17450.1 (T2_(4_Star))
AT3G17460.1 (T1_(5_Star))
AT3G17465.1 (RP
Pseudogenes
Noncoding RNAs
Arabidopsis cDNAs
BX824500
AY099622
AY084645
BX823326
BX823575
BX822842
BT001249
AK228799
AF370170
T-DNAs/Transposons
FLAG_511A02
SAIL_810_A04.V1
SAIL_1155_C01.V1
SALK_112952.54.75.X
SAIL_1284_G05.V1
SALK_023396.47.00.X
SALK_015242.55.40.X
GK-469E08-019027
SALK_146758.21.
WISCDSLOX3
GK-714B05-025
GK-714B05-025

AT3G22170

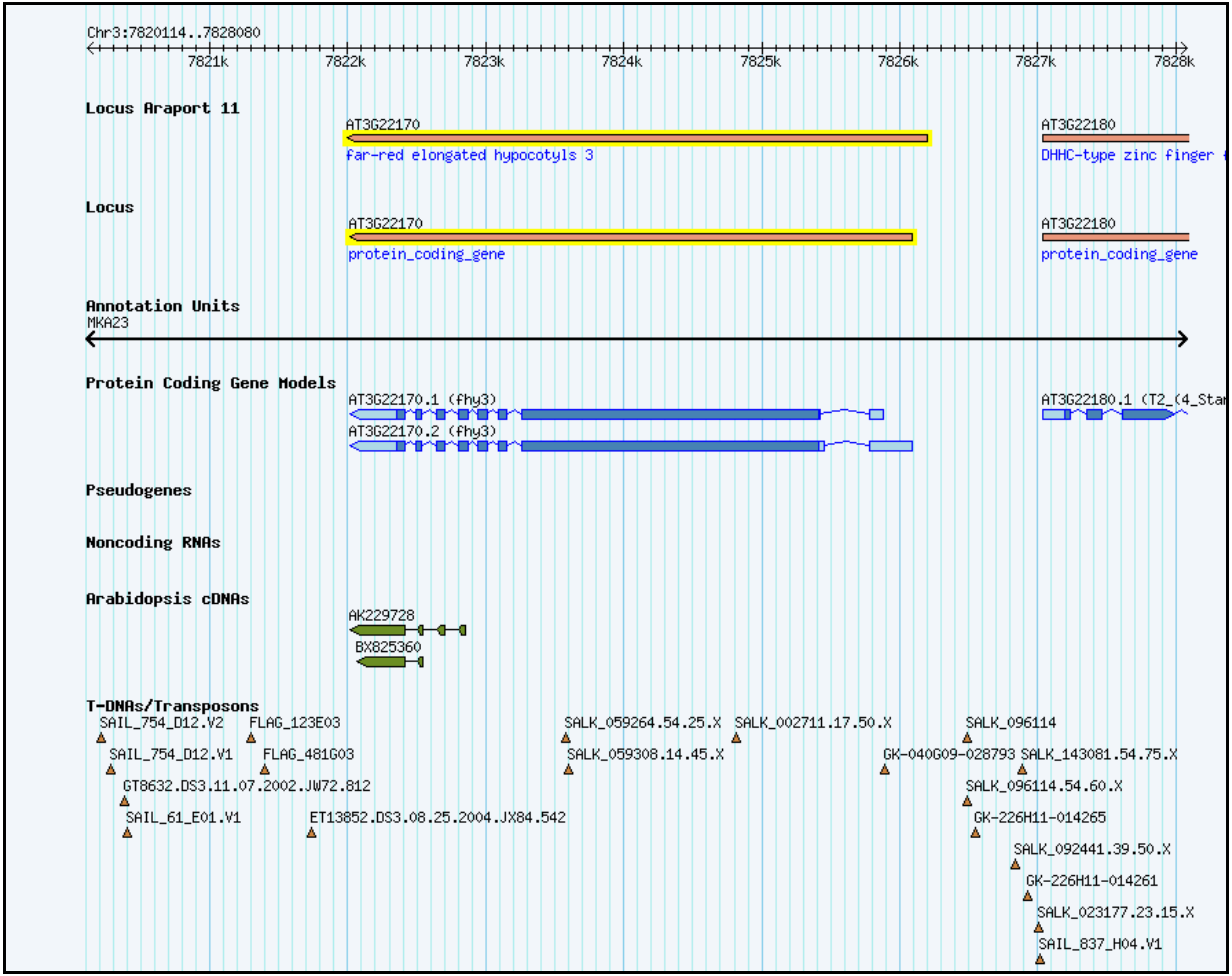
Chr3:7820114..7828080
7821k
7822k
7823k
7824k
7825k
7826k
7827k
7828k
Locus Araport 11
AT3G22170
far-red elongated hypocotyls 3
AT3G22180
DHHC-type zinc finger
Locus
AT3G22170
protein_coding_gene
AT3G22180
protein_coding_gene
Annotation Units
MKA23
Protein Coding Gene Models
AT3G22170.1 (fhy3)
AT3G22170.2 (fhy3)
Pseudogenes
Noncoding RNAs
Arabidopsis cDNAs
AK229728
BX825360
T-DNAs/Transposons
SAIL_754_D12.V2
FLAG_123E03
SALK_059264.54.25.X
SALK_002711.17.50.X
SALK_096114
SAIL_754_D12.V1
FLAG_481G03
SALK_059308.14.45.X
GK-040G09-028793
SALK_143081.54.75.X
GT8632.DS3.11.07.2002.JW72.812
SALK_096114.54.60.X
SAIL_61_E01.V1
ET13852.DS3.08.25.2004.JX84.542
GK-226H11-014265
SALK_092441.39.50.X
GK-226H11-014261
SALK_023177.23.15.X
SAIL_837_H04.V1

AT3G22220

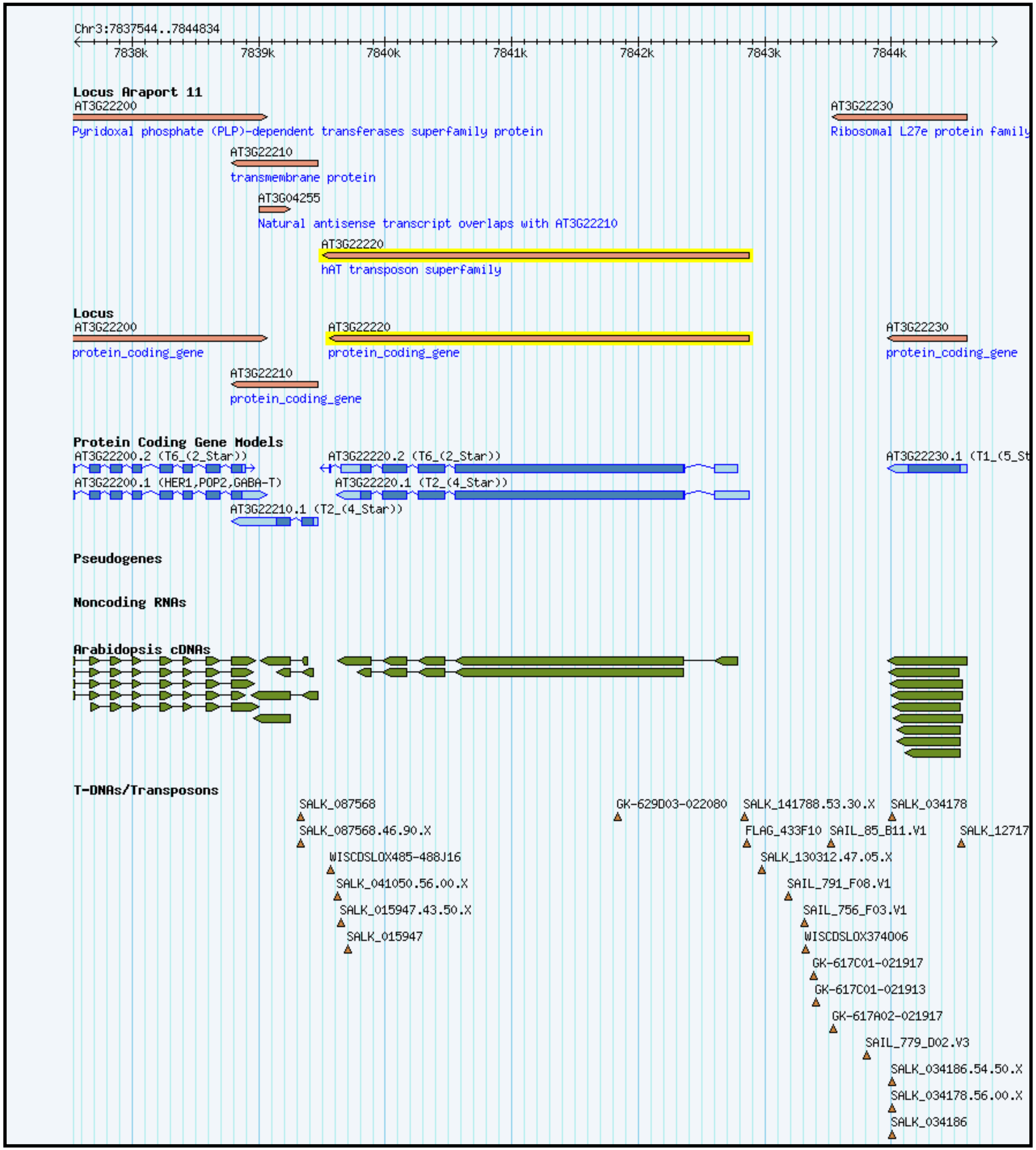

Chr3:7837544..7844834
7838k
7839k
7840k
7841k
7842k
7843k
7844k
Locus Araport 11
AT3G22200
Pyridoxal phosphate (PLP)-dependent transferases superfamily protein
AT3G22230
Ribosomal L27e protein family
AT3G22210
transmembrane protein
AT3G04255
Natural antisense transcript overlaps with AT3G22210
AT3G22220
hAT transposon superfamily
Locus
AT3G22200
protein_coding_gene
AT3G22220
protein_coding_gene
AT3G22230
protein_coding_gene
AT3G22210
protein_coding_gene
Protein Coding Gene Models
AT3G22200.2 (T6_(2_Star))
AT3G22220.2 (T6_(2_Star))
AT3G22230.1 (T1_(5_St
AT3G22200.1 (HER1,POP2,GABA-T)
AT3G22220.1 (T2_(4_Star))
AT3G22210.1 (T2_(4_Star))
Pseudogenes
Noncoding RNAs
Arabidopsis cDNAs
T-DNAs/Transposons
SALK_087568
GK-629D03-022080
SALK_141788.53.30.X
SALK_034178
SALK_087568.46.90.X
FLAG_433F10
SAIL_85_B11.V1
SALK_12717
WISCDSLOX485-488J16
SALK_130312.47.05.X
SALK_041050.56.00.X
SAIL_791_F08.V1
SALK_015947.43.50.X
SAIL_756_F03.V1
SALK_015947
WISCDSLOX374006
GK-617C01-021917
GK-617C01-021913
GK-617A02-021917
SAIL_779_D02.V3
SALK_034186.54.50.X
SALK_034178.56.00.X
SALK_034186

AT3G55350

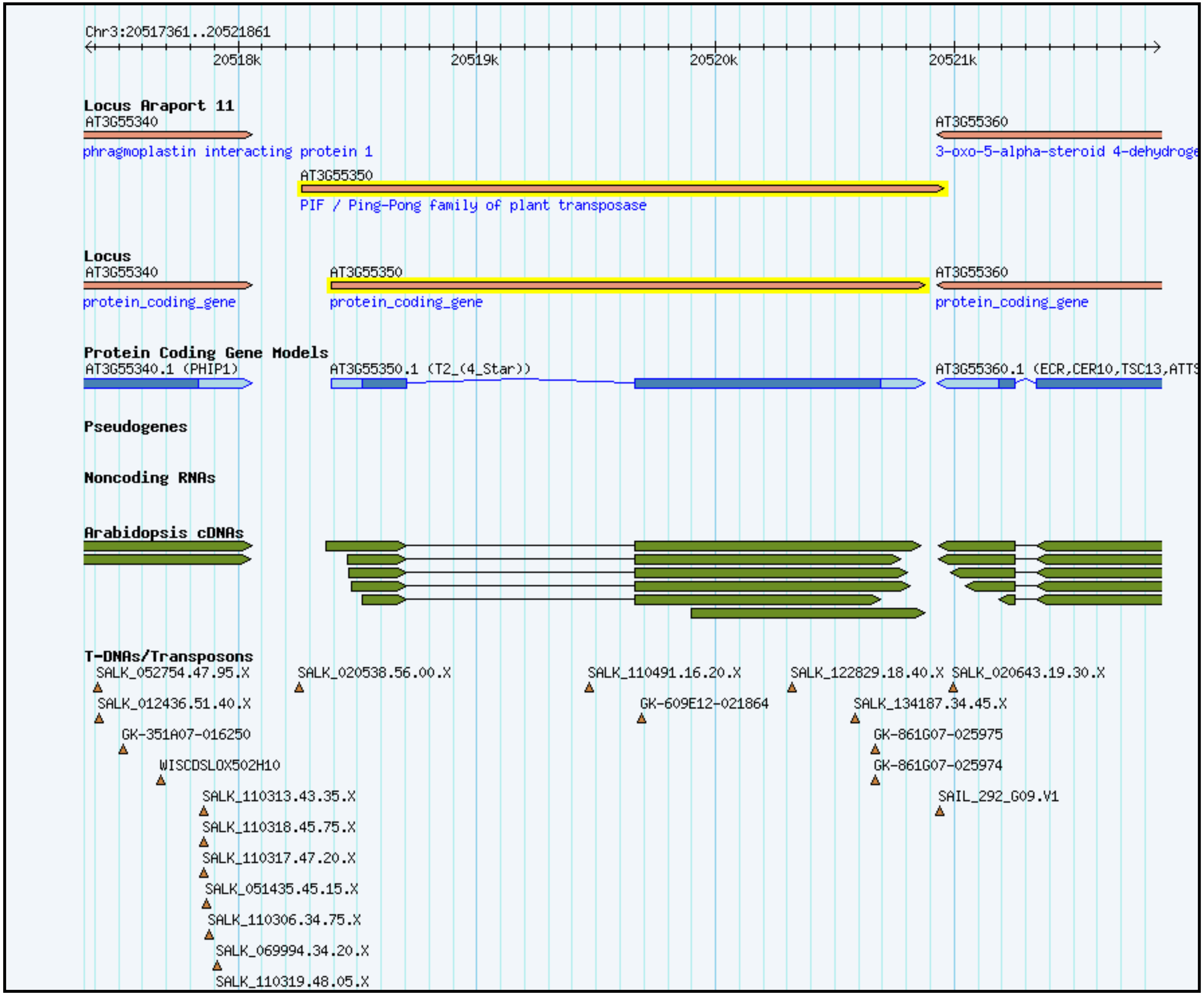
Chr3:20517361..20521861
20518k
20519k
20520k
20521k
Locus Araport 11
AT3G55340
phragmoplastin interacting protein 1
AT3G55360
3-oxo-5-alpha-steroid 4-dehydroge
AT3G55350
PIF / Ping-Pong family of plant transposase
Locus
AT3G55340
protein_coding_gene
AT3G55350
protein_coding_gene
AT3G55360
protein_coding_gene
Protein Coding Gene Models
AT3G55340.1 (PHIP1)
AT3G55350.1 (T2_(4_Star))
AT3G55360.1 (ECR,CER10,TSC13,ATTS
Pseudogenes
Noncoding RNAs
Arabidopsis cDNAs
T-DNAs/Transposons
SALK_052754.47.95.X
SALK_020538.56.00.X
SALK_110491.16.20.X
SALK_122829.18.40.X
SALK_020643.19.30.X
SALK_012436.51.40.X
GK-609E12-021864
SALK_134187.34.45.X
GK-351A07-016250
GK-861G07-025975
WISCDSLOX502H10
GK-861G07-025974
SALK_110313.43.35.X
SAIL_292_G09.V1
SALK_110318.45.75.X
SALK_110317.47.20.X
SALK_051435.45.15.X
SALK_110306.34.75.X
SALK_069994.34.20.X
SALK_110319.48.05.X

AT4G08220

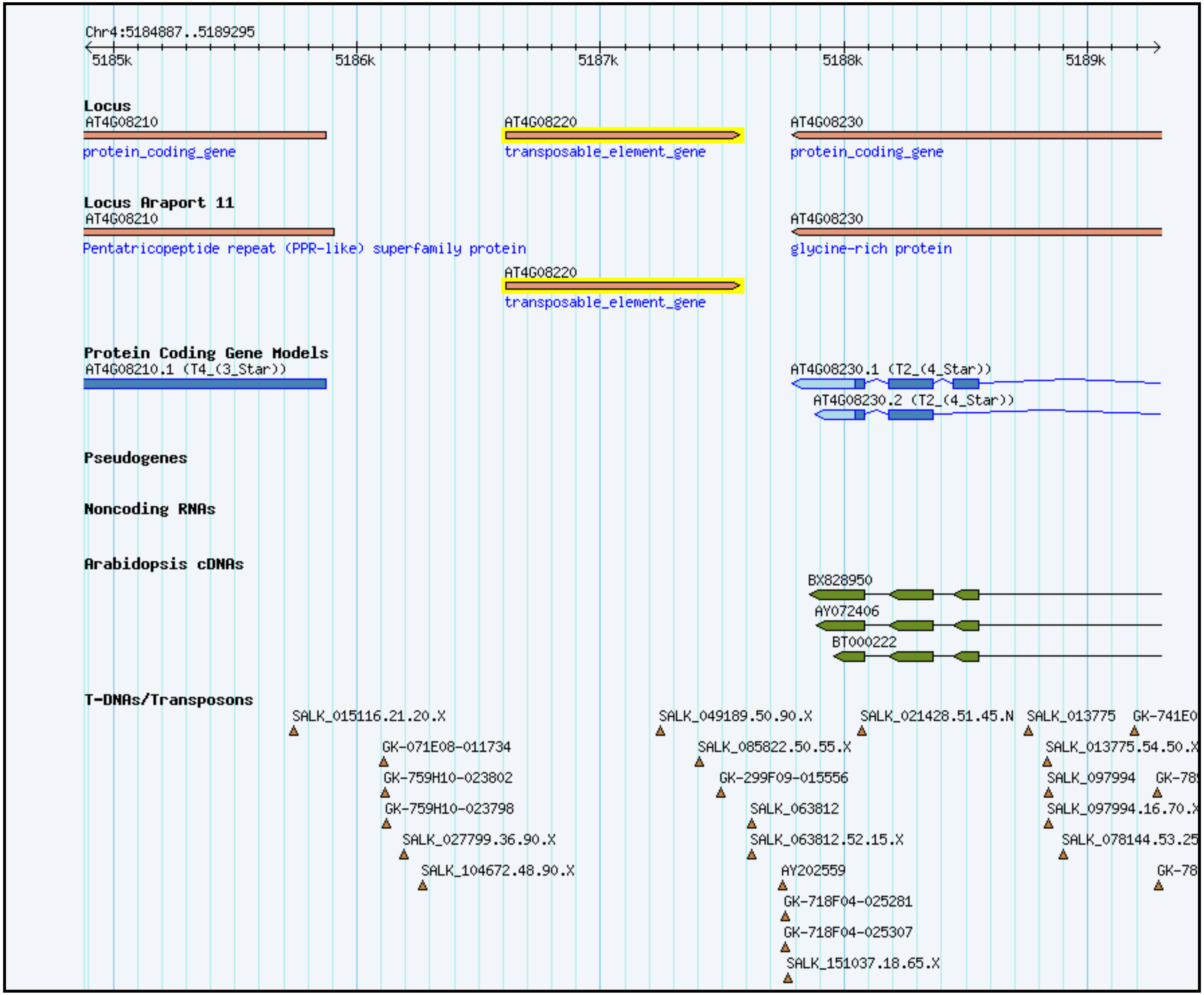
Chr4:5184887..5189295
5185k
5186k
5187k
5188k
5189k
Locus
AT4G08210
protein_coding_gene
AT4G08220
transposable_element_gene
AT4G08230
protein_coding_gene
Locus Araport 11
AT4G08210
Pentatricopeptide repeat (PPR-like) superfamily protein
AT4G08230
glycine-rich protein
AT4G08220
transposable_element_gene
Protein Coding Gene Models
AT4G08210.1 (T4_(3_Star))
AT4G08230.1 (T2_(4_Star))
AT4G08230.2 (T2_(4_Star))
Pseudogenes
Noncoding RNAs
Arabidopsis cDNAs
BX828950
AY072406
BT000222
T-DNAs/Transposons
SALK_015116.21.20.X
GK-071E08-011734
GK-759H10-023802
GK-759H10-023798
SALK_027799.36.90.X
SALK_104672.48.90.X
SALK_049189.50.90.X
SALK_085822.50.55.X
GK-299F09-015556
SALK_063812
SALK_063812.52.15.X
AY202559
GK-718F04-025281
GK-718F04-025307
SALK_151037.18.65.X
SALK_021428.51.45.N
SALK_013775
SALK_013775.54.50.X
SALK_097994
SALK_097994.16.70.X
SALK_078144.53.25

AT4G13120

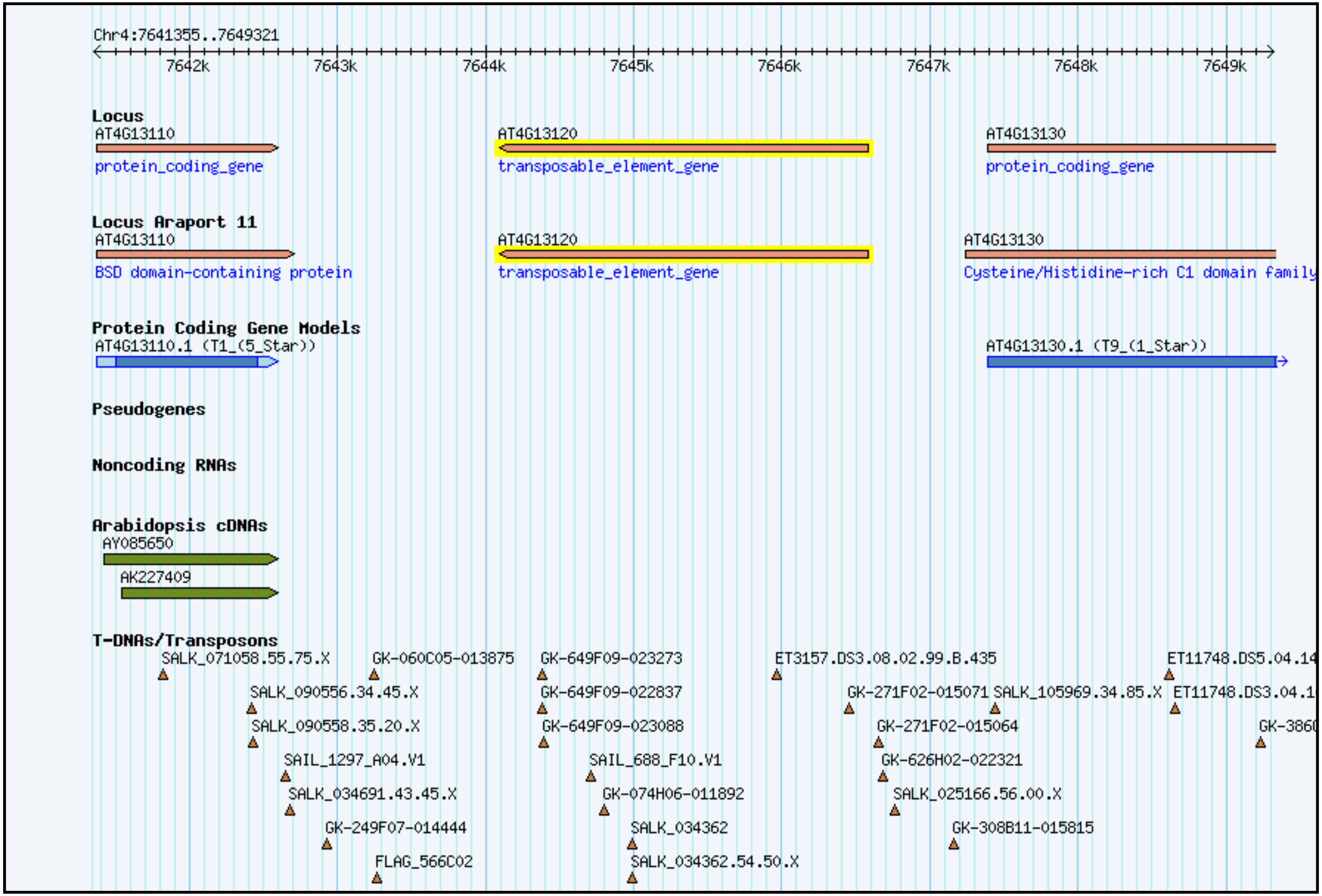

Chr4:7641355..7649321
7642k
7643k
7644k
7645k
7646k
7647k
7648k
7649k
Locus
AT4G13110
protein_coding_gene
AT4G13120
transposable_element_gene
AT4G13130
protein_coding_gene
Locus Araport 11
AT4G13110
BSD domain-containing protein
AT4G13120
transposable_element_gene
AT4G13130
Cysteine/Histidine-rich C1 domain family
Protein Coding Gene Models
AT4G13110.1 (T1_(5_Star))
AT4G13130.1 (T9_(1_Star))
Pseudogenes
Noncoding RNAs
Arabidopsis cDNAs
AY085650
AK227409
T-DNAs/Transposons
SALK_071058.55.75.X
GK-060C05-013875
GK-649F09-023273
ET3157.DS3.08.02.99.B.435
SALK_090556.34.45.X
GK-649F09-022837
GK-271F02-015071
SALK_105969.34.85.X
SALK_090558.35.20.X
GK-649F09-023088
GK-271F02-015064
SAIL_1297_A04.V1
SAIL_688_F10.V1
GK-626H02-022321
SALK_034691.43.45.X
GK-074H06-011892
SALK_025166.56.00.X
GK-249F07-014444
SALK_034362
GK-308B11-015815
FLAG_566C02
SALK_034362.54.50.X

AT4G15020

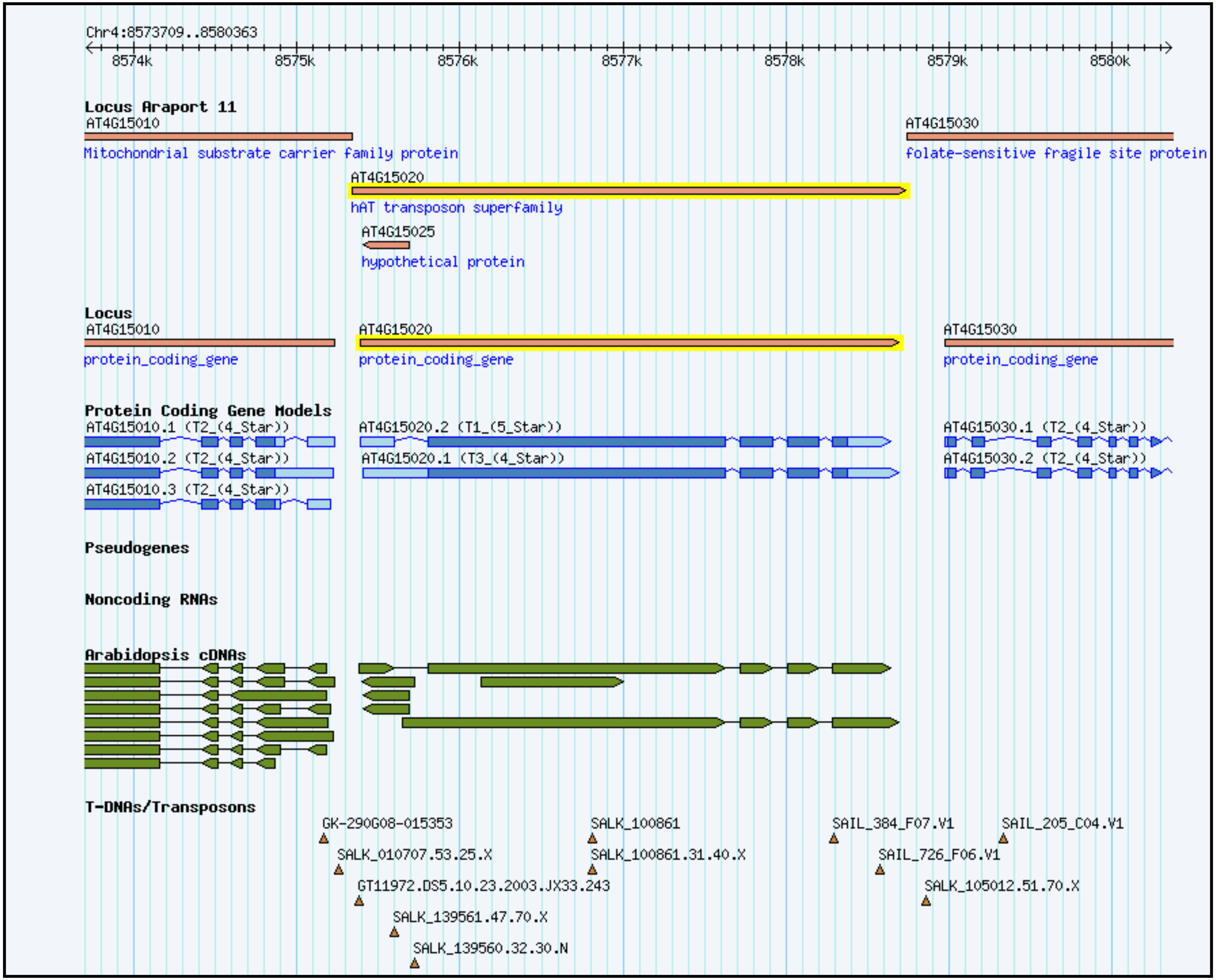
Chr4:8573709..8580363
8574k
8575k
8576k
8577k
8578k
8579k
8580k
Locus Araport 11
AT4G15010
Mitochondrial substrate carrier family protein
AT4G15030
folate-sensitive fragile site protein
AT4G15020
hAT transposon superfamily
AT4G15025
hypothetical protein
Locus
AT4G15010
protein_coding_gene
AT4G15020
protein_coding_gene
AT4G15030
protein_coding_gene
Protein Coding Gene Models
AT4G15010.1 (T2_(4_Star))
AT4G15010.2 (T2_(4_Star))
AT4G15010.3 (T2_(4_Star))
AT4G15020.2 (T1_(5_Star))
AT4G15020.1 (T3_(4_Star))
AT4G15030.1 (T2_(4_Star))
AT4G15030.2 (T2_(4_Star))
Pseudogenes
Noncoding RNAs
Arabidopsis cDNAs
T-DNAs/Transposons
GK-290G08-015353
SALK_010707.53.25.X
GT11972.DS5.10.23.2003.JX33.243
SALK_139561.47.70.X
SALK_139560.32.30.N
SALK_100861
SALK_100861.31.40.X
SAIL_384_F07.V1
SAIL_726_F06.V1
SALK_105012.51.70.X
SAIL_205_C04.V1

AT4G15090

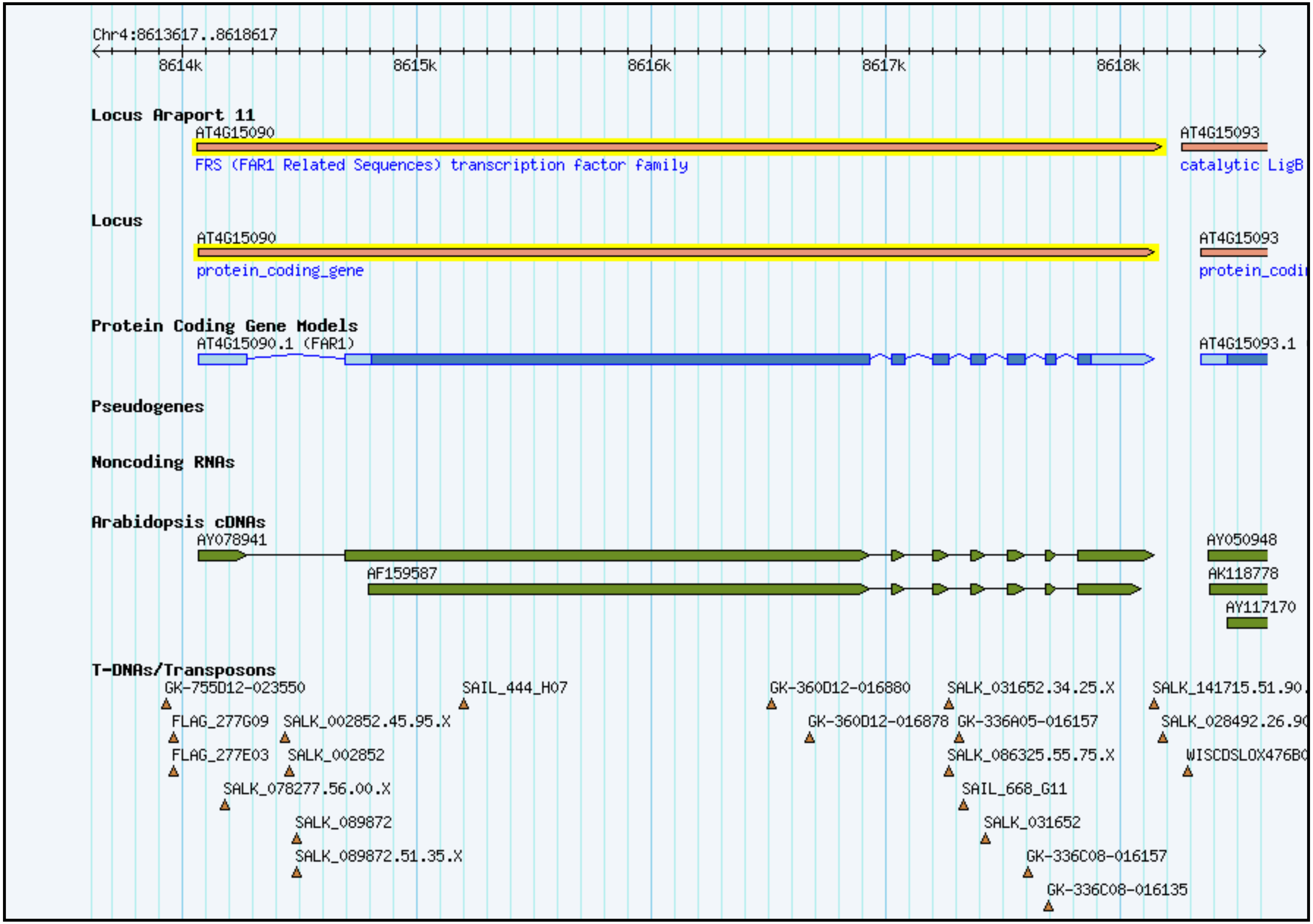

Chr4:8613617..8618617
8614k
8615k
8616k
8617k
8618k
Locus Araport 11
AT4G15090
FRS (FAR1 Related Sequences) transcription factor family
AT4G15093
catalytic LigB
Locus
AT4G15090
protein_coding_gene
AT4G15093
Protein Coding Gene Models
AT4G15090.1 (FAR1)
AT4G15093.1
Pseudogenes
Noncoding RNAs
Arabidopsis cDNAs
AY078941
AF159587
AY050948
AK118778
AY117170
T-DNAs/Transposons
GK-755D12-023550
FLAG_277G09
FLAG_277E03
SALK_002852.45.95.X
SALK_002852
SALK_078277.56.00.X
SALK_089872
SALK_089872.51.35.X
SAIL_444_H07
GK-360D12-016880
GK-360D12-016878
SALK_031652.34.25.X
GK-336A05-016157
SALK_086325.55.75.X
SAIL_668_G11
SALK_031652
GK-336C08-016157
GK-336C08-016135

AT4G19990

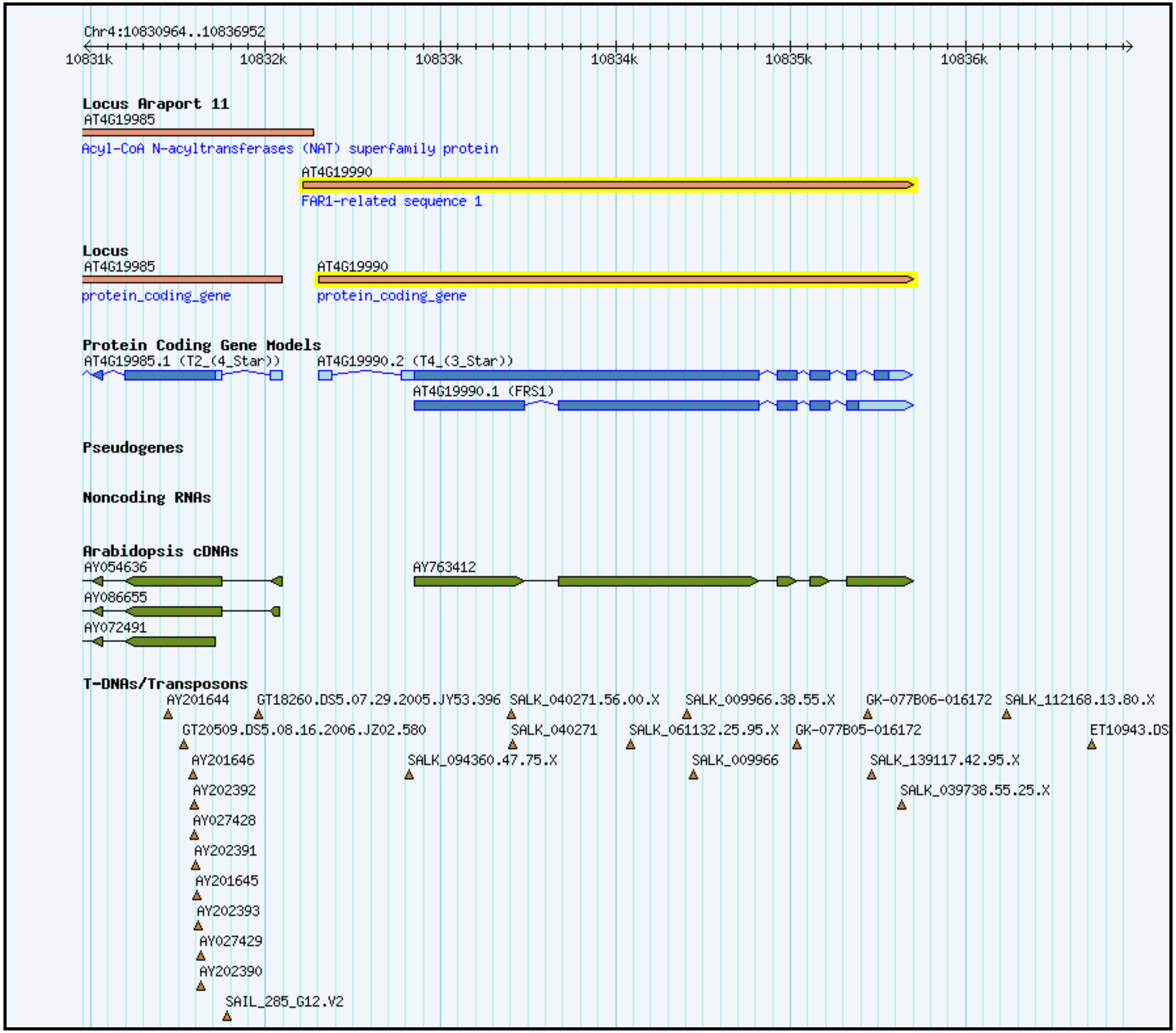

Chr4:10830964..10836952
10831k
10832k
10833k
10834k
10835k
10836k
Locus Araport 11
AT4G19985
Acyl-CoA N-acyltransferases (NAT) superfamily protein
AT4G19990
FAR1-related sequence 1
Locus
AT4G19985
protein_coding_gene
AT4G19990
protein_coding_gene
Protein Coding Gene Models
AT4G19985.1 (T2_(4_Star))
AT4G19990.2 (T4_(3_Star))
AT4G19990.1 (FRS1)
Pseudogenes
Noncoding RNAs
Arabidopsis cDNAs
AY054636
AY763412
AY086655
AY072491
T-DNAs/Transposons
AY201644
GT18260.DS5.07.29.2005.JY53.396
SALK_040271.56.00.X
SALK_009966.38.55.X
GK-077B06-016172
SALK_112168.13.80.X
GT20509.DS5.08.16.2006.JZ02.580
SALK_040271
SALK_061132.25.95.X
GK-077B05-016172
ET10943.DS
AY201646
SALK_094360.47.75.X
SALK_009966
SALK_139117.42.95.X
AY202392
SALK_039738.55.25.X
AY027428
AY202391
AY201645
AY202393
AY027429
AY202390
SAIL_285_G12.V2

AT4G38170

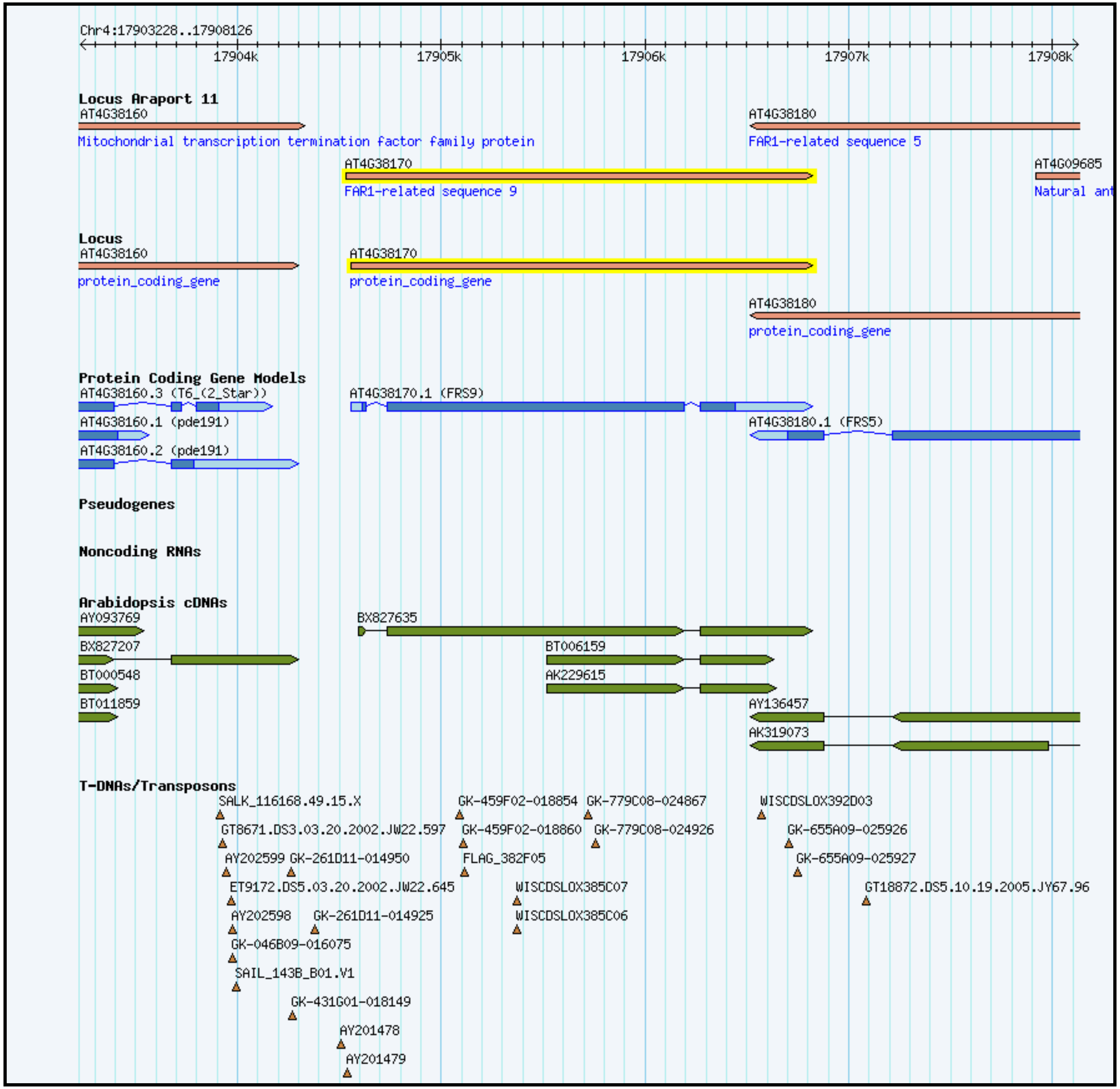
Chr4:17903228..17908126
17904k
17905k
17906k
17907k
17908k
Locus Araport 11
AT4G38160
Mitochondrial transcription termination factor family protein
AT4G38180
FAR1-related sequence 5
AT4G38170
FAR1-related sequence 9
AT4G09685
Natural ant
Locus
AT4G38160
protein_coding_gene
AT4G38170
protein_coding_gene
AT4G38180
protein_coding_gene
Protein Coding Gene Models
AT4G38160.3 (T6_(2_Star))
AT4G38170.1 (FRS9)
AT4G38160.1 (pde191)
AT4G38180.1 (FRS5)
AT4G38160.2 (pde191)
Pseudogenes
Noncoding RNAs
Arabidopsis cDNAs
AY093769
BX827635
BX827207
BT006159
BT000548
AK229615
BT011859
AY136457
AK319073
T-DNAs/Transposons
SALK_116168.49.15.X
GK-459F02-018854
GK-779C08-024867
WISCDSLOX392D03
GT8671.DS3.03.20.2002.JW22.597
GK-459F02-018860
GK-779C08-024926
GK-655A09-025926
AY202599
GK-261D11-014950
FLAG_382F05
GK-655A09-025927
ET9172.DS5.03.20.2002.JW22.645
WISCDSLOX385C07
GT18872.DS5.10.19.2005.JY67.96
AY202598
GK-261D11-014925
WISCDSLOX385C06
GK-046B09-016075
SAIL_143B_B01.V1
GK-431G01-018149
AY201478
AY201479

AT4G38180

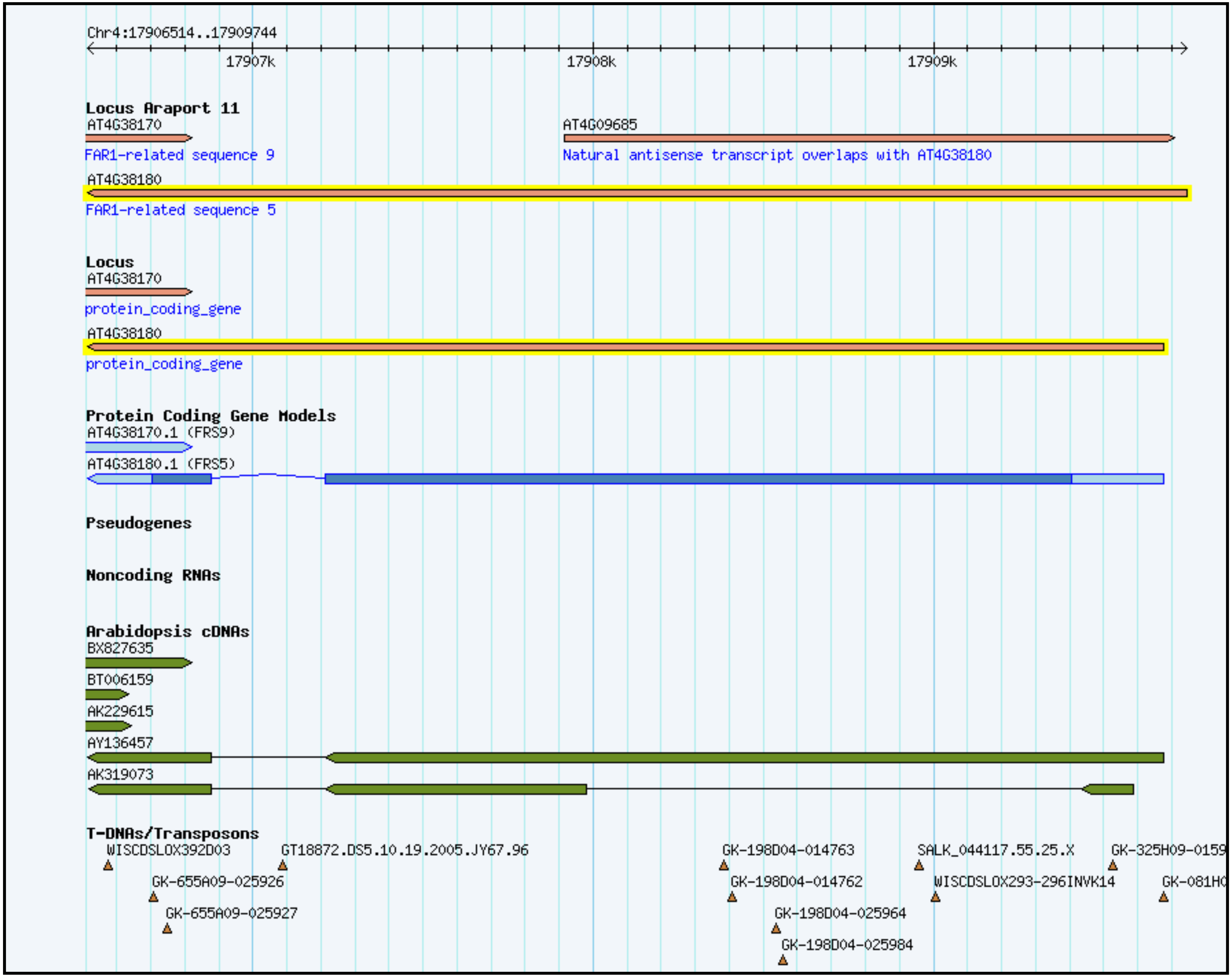
Chr4:17906514..17909744
17907k
17908k
17909k
Locus Araport 11
AT4G38170
FAR1-related sequence 9
AT4G09685
Natural antisense transcript overlaps with AT4G38180
AT4G38180
FAR1-related sequence 5
Locus
AT4G38170
protein_coding_gene
AT4G38180
protein_coding_gene
Protein Coding Gene Models
AT4G38170.1 (FRS9)
AT4G38180.1 (FRS5)
Pseudogenes
Noncoding RNAs
Arabidopsis cDNAs
BX827635
BT006159
AK229615
AY136457
AK319073
T-DNAs/Transposons
WISCDSLOX392D03
GT18872.DS5.10.19.2005.JY67.96
GK-198D04-014763
SALK_044117.55.25.X
GK-655A09-025926
GK-198D04-014762
WISCDSLOX293-296INVK14
GK-655A09-025927
GK-198D04-025964
GK-198D04-025984

AT5G16505

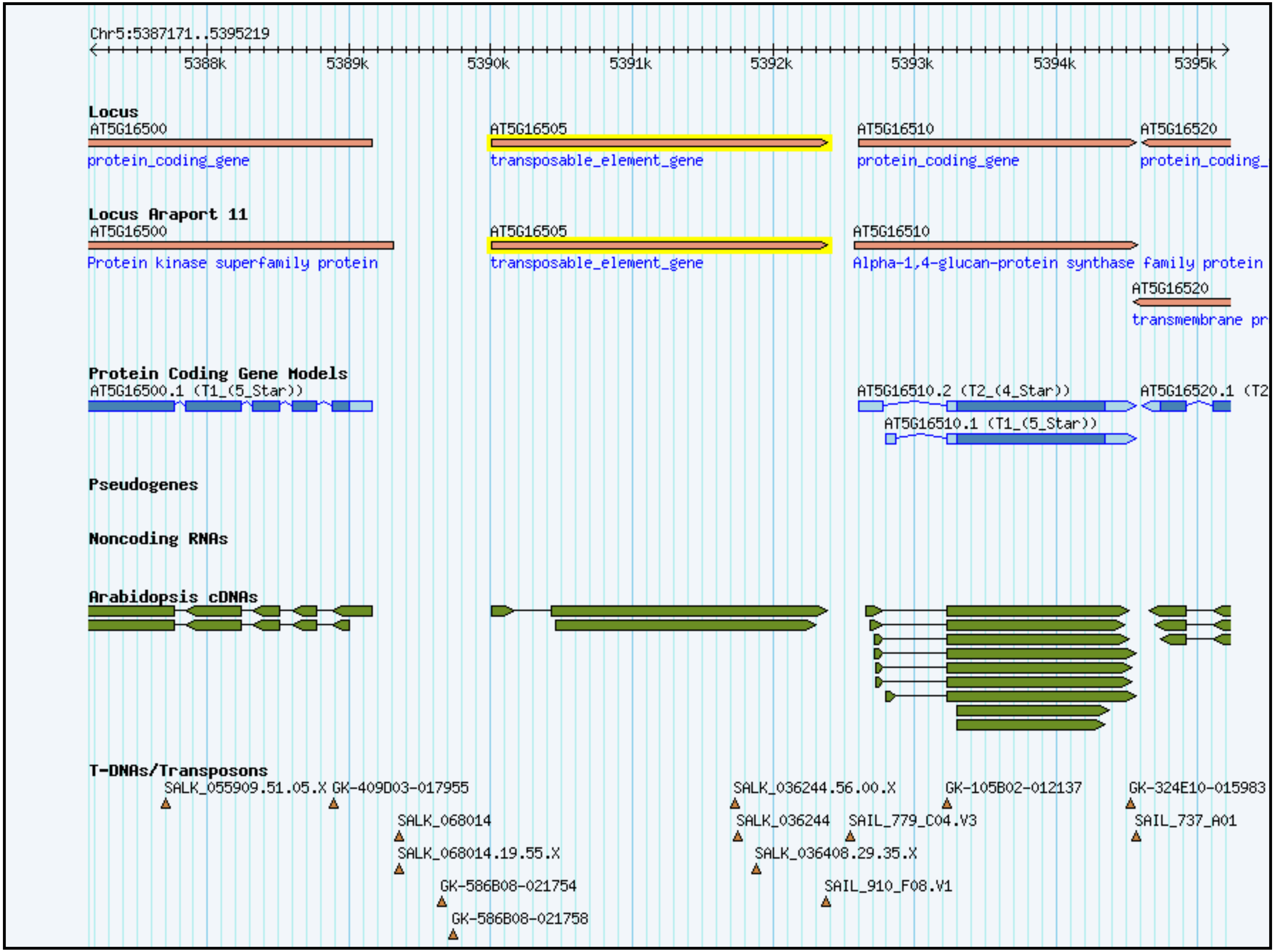
Chr5:5387171..5395219
5388k
5389k
5390k
5391k
5392k
5393k
5394k
5395k
Locus
AT5G16500
protein_coding_gene
AT5G16505
transposable_element_gene
AT5G16510
protein_coding_gene
AT5G16520
protein_coding_
Locus Araport 11
AT5G16500
Protein kinase superfamily protein
AT5G16505
transposable_element_gene
AT5G16510
Alpha-1,4-glucan-protein synthase family protein
AT5G16520
transmembrane pr
Protein Coding Gene Models
AT5G16500.1 (T1_(5_Star))
AT5G16510.2 (T2_(4_Star))
AT5G16520.1 (T2
AT5G16510.1 (T1_(5_Star))
Pseudogenes
Noncoding RNAs
Arabidopsis cDNAs
T-DNAs/Transposons
SALK_055909.51.05.X
GK-409D03-017955
SALK_068014
SALK_068014.19.55.X
GK-586B08-021754
GK-586B08-021758
SALK_036244.56.00.X
SALK_036244
SALK_036408.29.35.X
SAIL_910_F08.V1
GK-105B02-012137
SAIL_779_C04.V3
GK-324E10-015983
SAIL_737_A01

AT5G20880

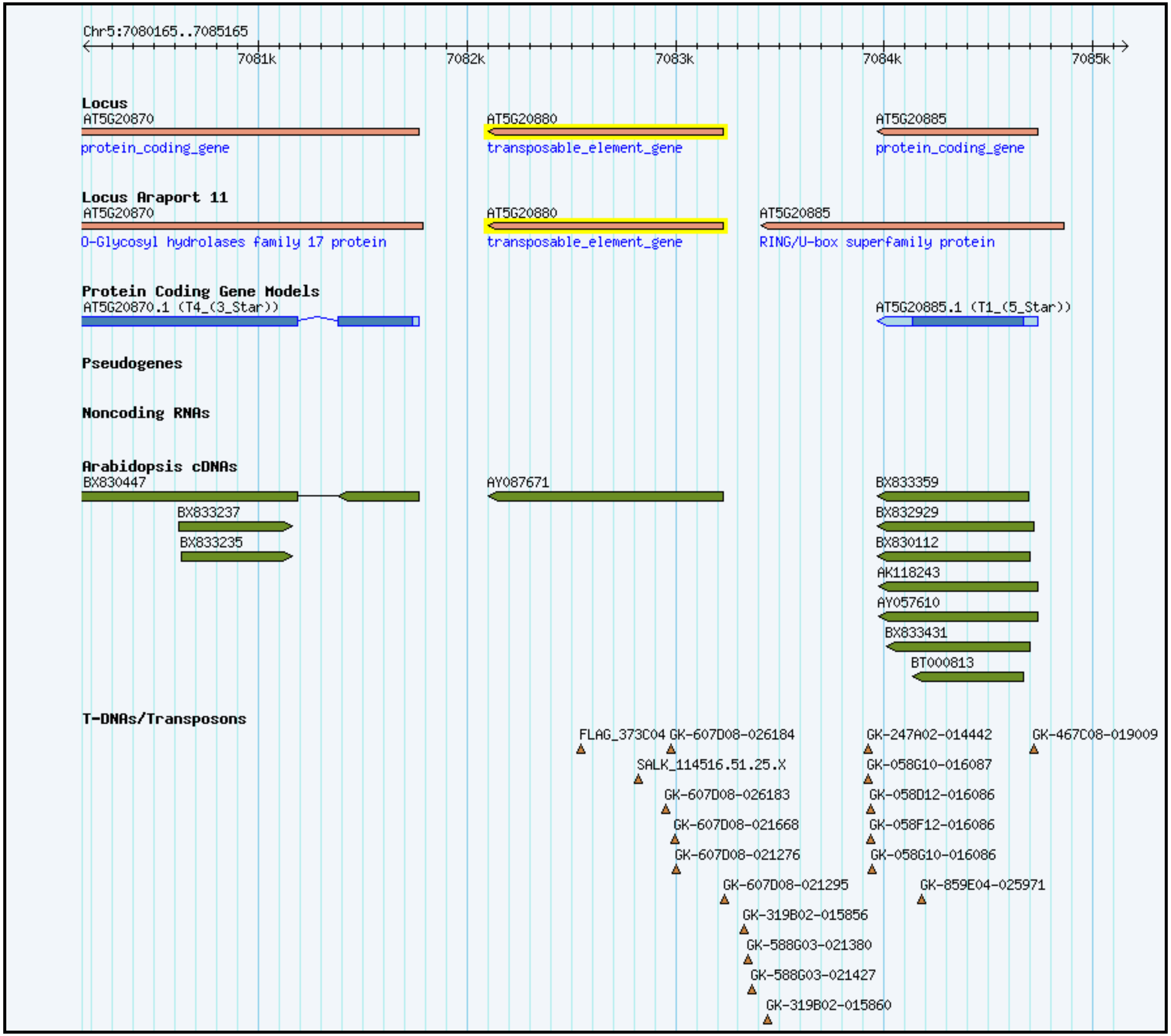
Chr5:7080165..7085165
7081k
7082k
7083k
7084k
7085k
Locus
AT5G20870
protein_coding_gene
AT5G20880
transposable_element_gene
AT5G20885
protein_coding_gene
Locus Araport 11
AT5G20870
O-Glycosyl hydrolases family 17 protein
AT5G20880
transposable_element_gene
AT5G20885
RING/U-box superfamily protein
Protein Coding Gene Models
AT5G20870.1 (T4_(3_Star))
AT5G20885.1 (T1_(5_Star))
Pseudogenes
Noncoding RNAs
Arabidopsis cDNAs
BX830447
BX833237
BX833235
AY087671
BX833359
BX832929
BX830112
AK118243
AY057610
BX833431
BT000813
T-DNAs/Transposons
FLAG_373C04
GK-607D08-026184
SALK_114516.51.25.X
GK-607D08-026183
GK-607D08-021668
GK-607D08-021276
GK-607D08-021295
GK-319B02-015856
GK-588G03-021380
GK-588G03-021427
GK-319B02-015860
GK-247A02-014442
GK-058G10-016087
GK-058D12-016086
GK-058F12-016086
GK-058G10-016086
GK-859E04-025971
GK-467C08-019009

AT5G28530

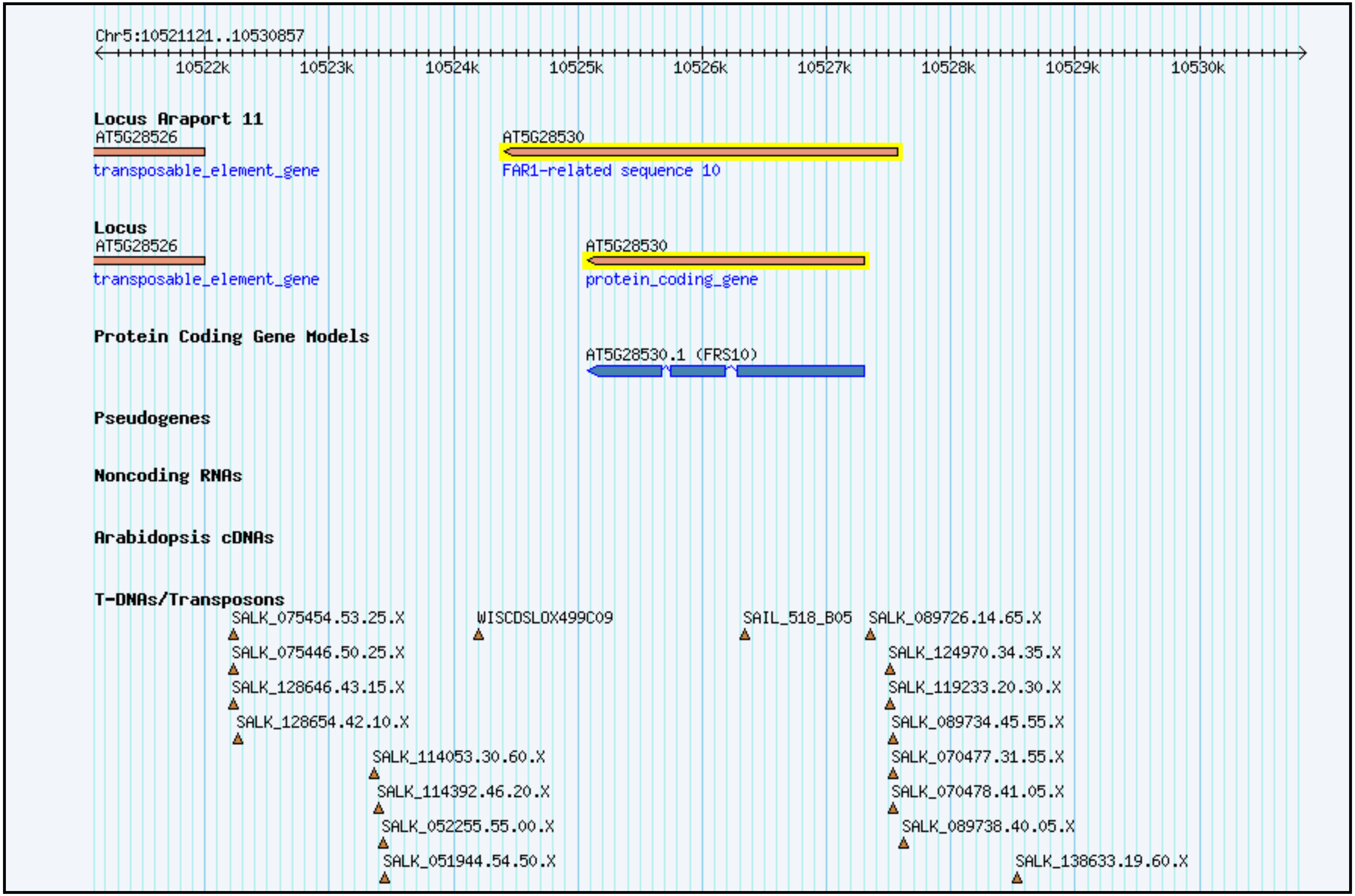

Chr5:10521121..10530857
10522k
10523k
10524k
10525k
10526k
10527k
10528k
10529k
10530k
Locus Araport 11
AT5G28526
transposable_element_gene
AT5G28530
FAR1-related sequence 10
Locus
AT5G28526
transposable_element_gene
AT5G28530
protein_coding_gene
Protein Coding Gene Models
AT5G28530.1 (FRS10)
Pseudogenes
Noncoding RNAs
Arabidopsis cDNAs
T-DNAs/Transposons
SALK_075454.53.25.X
SALK_075446.50.25.X
SALK_128646.43.15.X
SALK_128654.42.10.X
SALK_114053.30.60.X
SALK_114392.46.20.X
SALK_052255.55.00.X
SALK_051944.54.50.X
WISCDSLOX499C09
SAIL_518_B05
SALK_089726.14.65.X
SALK_124970.34.35.X
SALK_119233.20.30.X
SALK_089734.45.55.X
SALK_070477.31.55.X
SALK_070478.41.05.X
SALK_089738.40.05.X
SALK_138633.19.60.X

AT5G34853

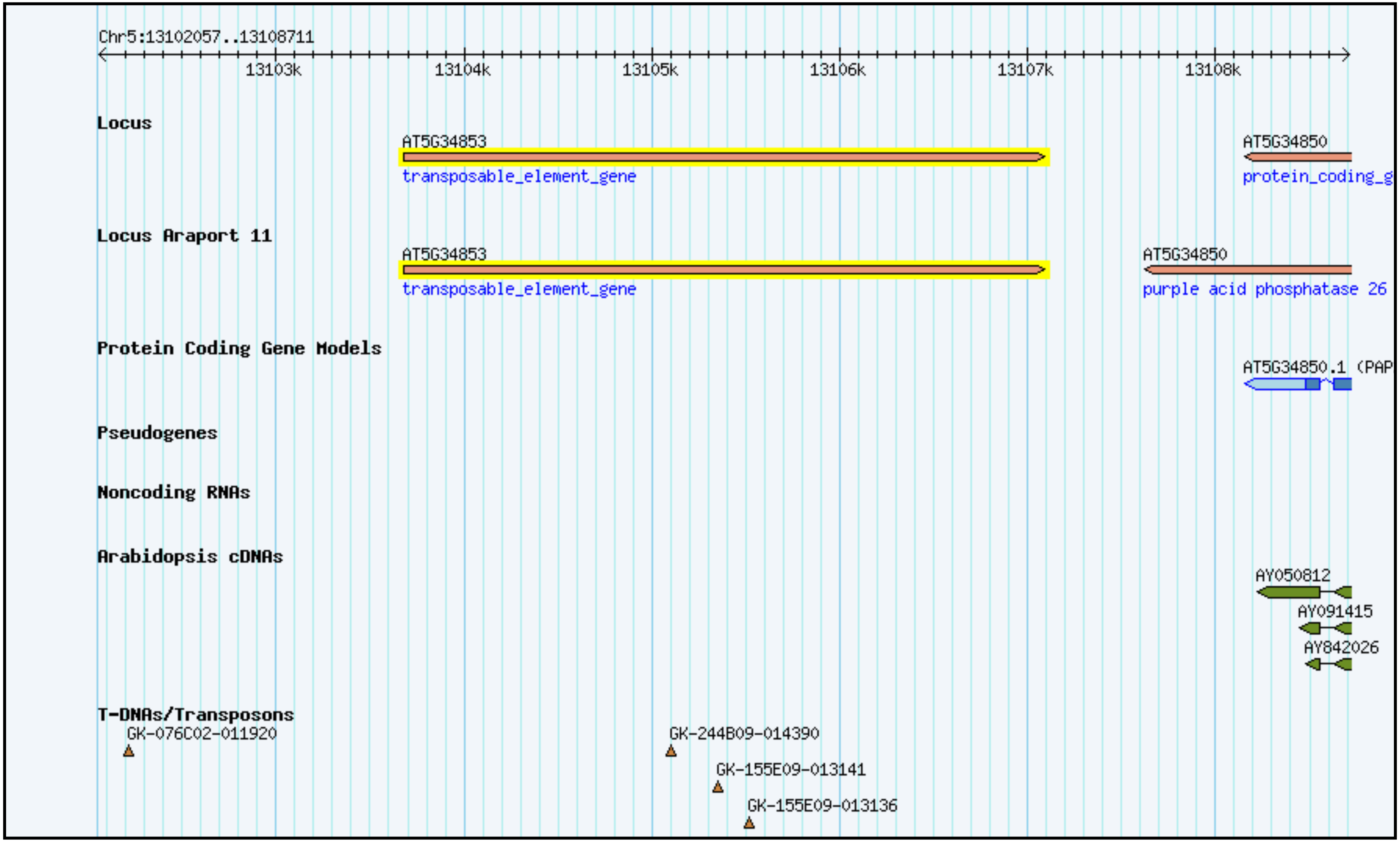
Chr5:13102057..13108711
13103k
13104k
13105k
13106k
13107k
13108k
Locus
AT5G34853
transposable_element_gene
AT5G34850
protein_coding_g
Locus Araport 11
AT5G34853
transposable_element_gene
AT5G34850
purple acid phosphatase 26
Protein Coding Gene Models
AT5G34850.1 (PAP
Pseudogenes
Noncoding RNAs
Arabidopsis cDNAs
AY050812
AY091415
AY842026
T-DNAs/Transposons
GK-076C02-011920
GK-244B09-014390
GK-155E09-013141
GK-155E09-013136

AT5G41710

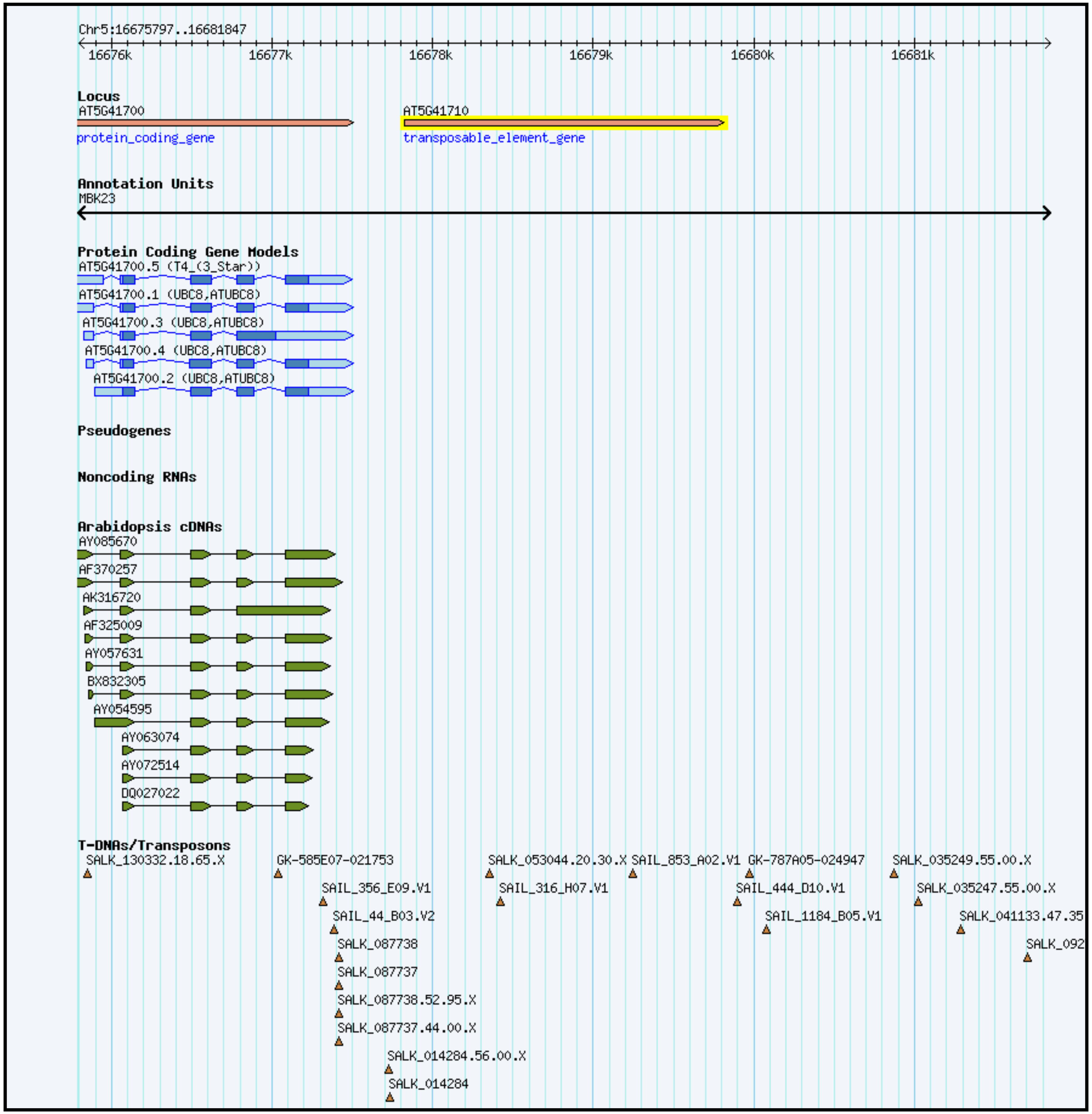
Chr5:16675797..16681847
16676k
16677k
16678k
16679k
16680k
16681k
Locus
AT5G41700
protein_coding_gene
AT5G41710
transposable_element_gene
Annotation Units
MBK23
Protein Coding Gene Models
AT5G41700.5 (T4_(3_Star))
AT5G41700.1 (UBC8,ATUBC8)
AT5G41700.3 (UBC8,ATUBC8)
AT5G41700.4 (UBC8,ATUBC8)
AT5G41700.2 (UBC8,ATUBC8)
Pseudogenes
Noncoding RNAs
Arabidopsis cDNAs
AY085670
AF370257
AK316720
AF325009
AY057631
BX832305
AY054595
AY063074
AY072514
DQ027022
T-DNAs/Transposons
SALK_130332.18.65.X
GK-585E07-021753
SAIL_356_E09.V1
SAIL_44_B03.V2
SALK_087738
SALK_087737
SALK_087738.52.95.X
SALK_087737.44.00.X
SALK_014284.56.00.X
SALK_014284
SALK_053044.20.30.X
SAIL_316_H07.V1
SAIL_853_A02.V1
GK-787A05-024947
SAIL_444_D10.V1
SAIL_1184_B05.V1
SALK_035249.55.00.X
SALK_035247.55.00.X
SALK_041133.47.35
SALK_092

AT5G41980

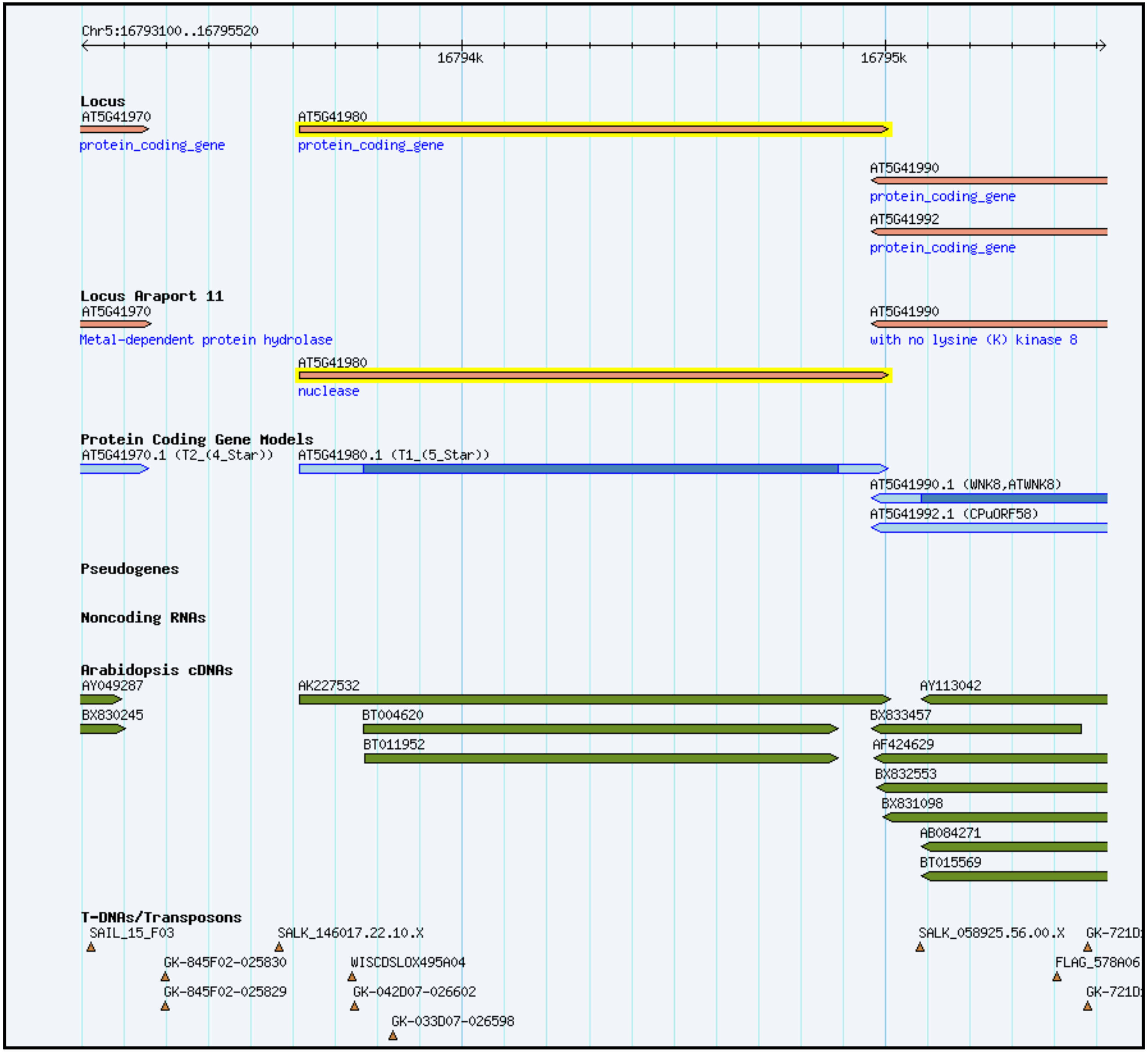
Chr5:16793100..16795520
16794k
16795k
Locus
AT5G41970
protein_coding_gene
AT5G41980
protein_coding_gene
AT5G41990
protein_coding_gene
AT5G41992
protein_coding_gene
Locus Araport 11
AT5G41970
Metal-dependent protein hydrolase
AT5G41990
with no lysine (K) kinase 8
AT5G41980
nuclease
Protein Coding Gene Models
AT5G41970.1 (T2_(4_Star))
AT5G41980.1 (T1_(5_Star))
AT5G41990.1 (WNK8,ATWNK8)
AT5G41992.1 (CPuORF58)
Pseudogenes
Noncoding RNAs
Arabidopsis cDNAs
AY049287
AK227532
AY113042
BX830245
BT004620
BX833457
BT011952
AF424629
BX832553
BX831098
AB084271
BT015569
T-DNAs/Transposons
SAIL_15_F03
SALK_146017.22.10.X
SALK_058925.56.00.X
GK-721D
GK-845F02-025830
WISCDSLOX495A04
FLAG_578A06
GK-845F02-025829
GK-042D07-026602
GK-721D
GK-033D07-026598

AT5G48965

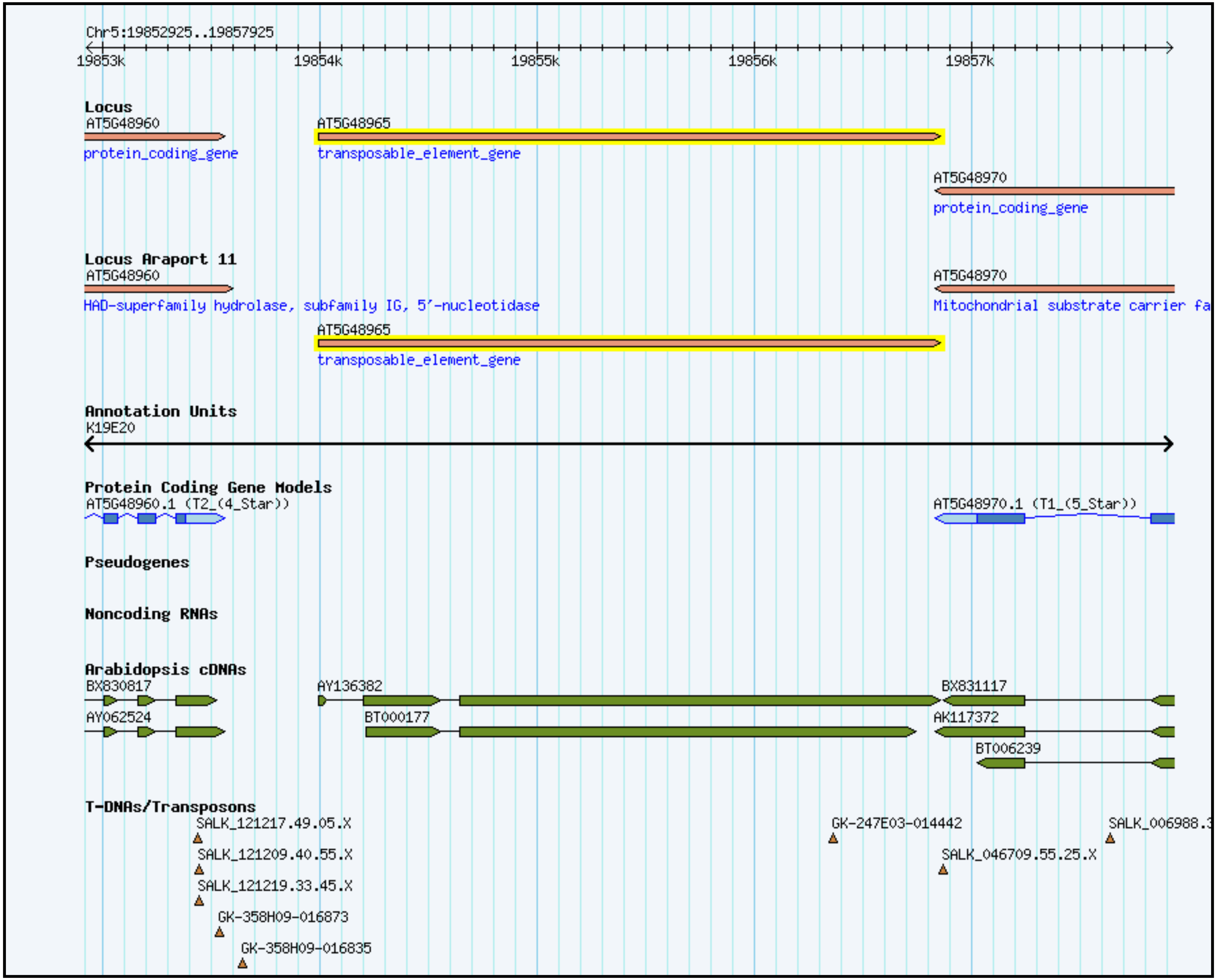
Chr5:19852925..19857925
19853k
19854k
19855k
19856k
19857k
Locus
AT5G48960
protein_coding_gene
AT5G48965
transposable_element_gene
AT5G48970
protein_coding_gene
Locus Araport 11
AT5G48960
HAD-superfamily hydrolase, subfamily IG, 5'-nucleotidase
AT5G48970
Mitochondrial substrate carrier fa
AT5G48965
transposable_element_gene
Annotation Units
K19E20
Protein Coding Gene Models
AT5G48960.1 (T2_(4_Star))
AT5G48970.1 (T1_(5_Star))
Pseudogenes
Noncoding RNAs
Arabidopsis cDNAs
BX830817
AY136382
BX831117
AY062524
BT000177
AK117372
BT006239
T-DNAs/Transposons
SALK_121217.49.05.X
GK-247E03-014442
SALK_006988.3
SALK_121209.40.55.X
SALK_046709.55.25.X
SALK_121219.33.45.X
GK-358H09-016873
GK-358H09-016835

AT5G50315

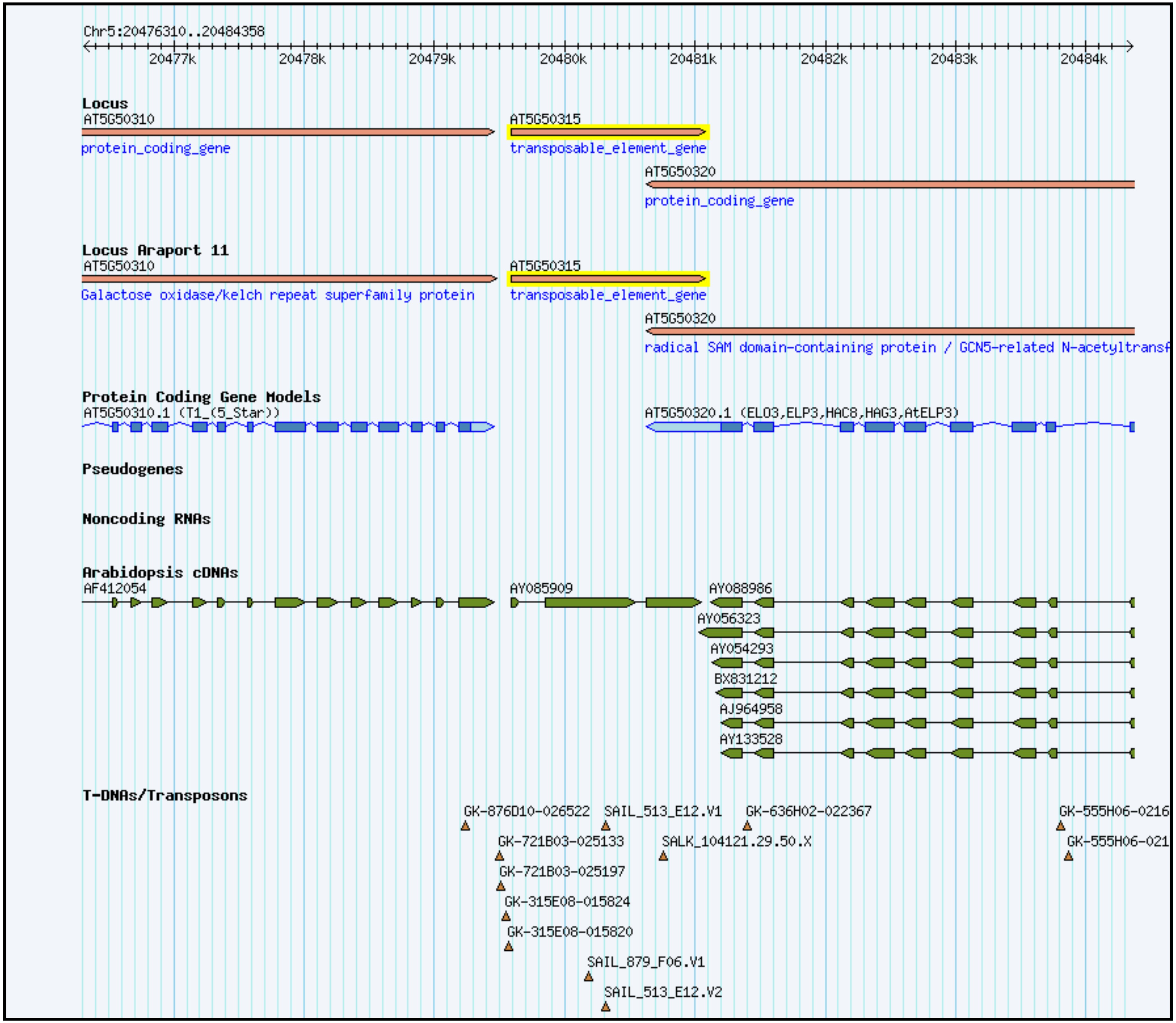

Chr5:20476310..20484358
20477k
20478k
20479k
20480k
20481k
20482k
20483k
20484k
Locus
AT5G50310
protein_coding_gene
AT5G50315
transposable_element_gene
AT5G50320
protein_coding_gene
Locus Araport 11
AT5G50310
Galactose oxidase/kelch repeat superfamily protein
AT5G50315
transposable_element_gene
AT5G50320
radical SAM domain-containing protein / GCN5-related N-acetyltransf
Protein Coding Gene Models
AT5G50310.1 (T1_(5_Star))
AT5G50320.1 (ELO3,ELP3,HAC8,HAG3,AtELP3)
Pseudogenes
Noncoding RNAs
Arabidopsis cDNAs
AF412054
AY085909
AY088986
AY056323
AY054293
BX831212
AJ964958
AY133528
T-DNAs/Transposons
GK-876D10-026522
SAIL_513_E12.V1
GK-636H02-022367
GK-555H06-0216
GK-721B03-025133
SALK_104121.29.50.X
GK-555H06-021
GK-721B03-025197
GK-315E08-015824
GK-315E08-015820
SAIL_879_F06.V1
SAIL_513_E12.V2

AT5G51080

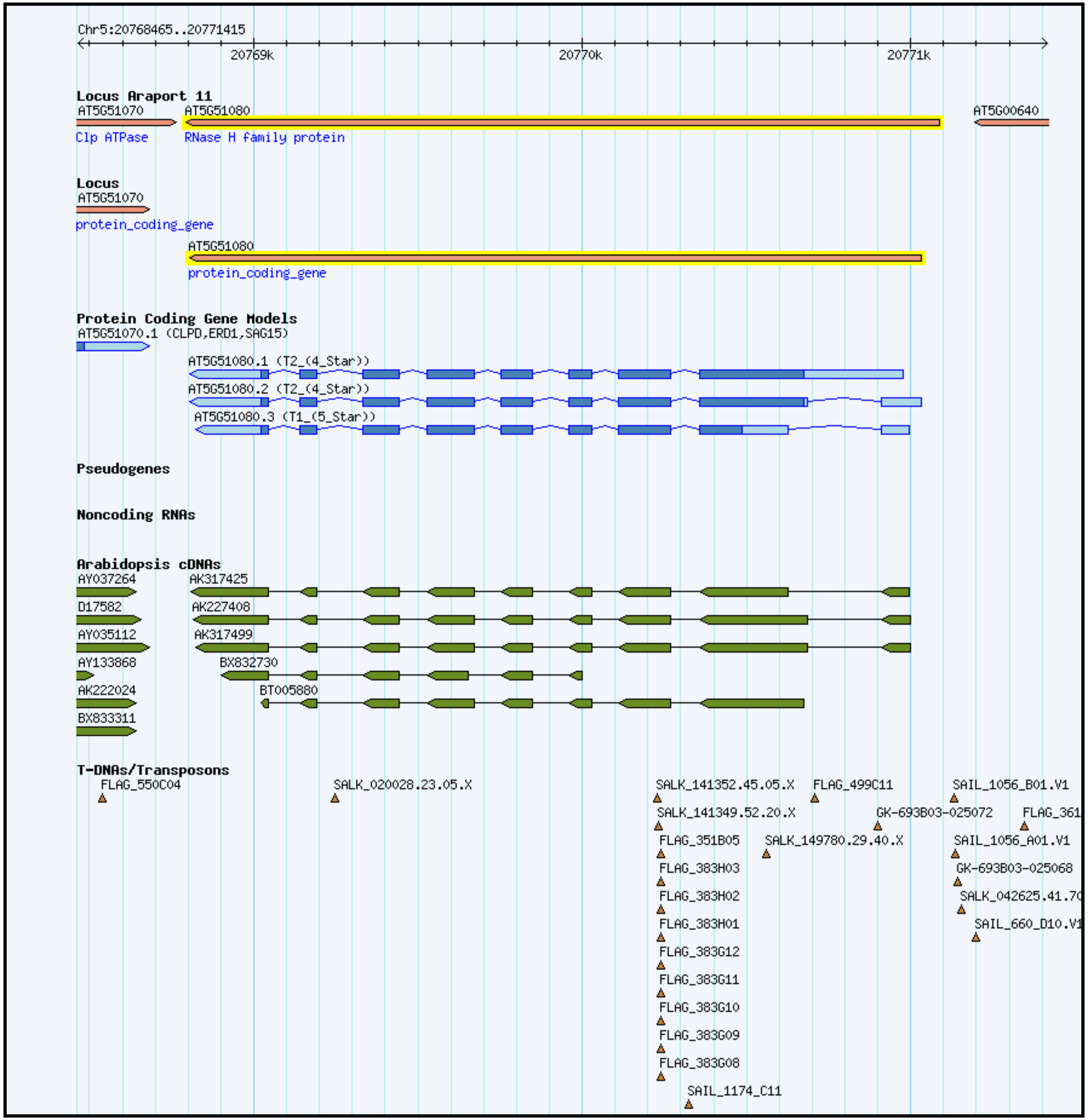
Chr5:20768465..20771415
20769k
20770k
20771k
Locus Araport 11
AT5G51070
AT5G51080
AT5G00640
Clp ATPase
RNase H family protein
Locus
AT5G51070
protein_coding_gene
AT5G51080
protein_coding_gene
Protein Coding Gene Models
AT5G51070.1 (CLPD,ERD1,SAG15)
AT5G51080.1 (T2_(4_Star))
AT5G51080.2 (T2_(4_Star))
AT5G51080.3 (T1_(5_Star))
Pseudogenes
Noncoding RNAs
Arabidopsis cDNAs
AY037264
AK317425
D17582
AK227408
AY035112
AK317499
AY133868
BX832730
AK222024
BT005880
BX833311
T-DNAs/Transposons
FLAG_550C04
SALK_020028.23.05.X
SALK_141352.45.05.X
FLAG_499C11
SAIL_1056_B01.V1
SALK_141349.52.20.X
GK-693B03-025072
FLAG_361
FLAG_351B05
SALK_149780.29.40.X
SAIL_1056_A01.V1
FLAG_383H03
GK-693B03-025068
FLAG_383H02
SALK_042625.41.70
FLAG_383H01
SAIL_660_D10.V1
FLAG_383G12
FLAG_383G11
FLAG_383G10
FLAG_383G09
FLAG_383G08
SAIL_1174_C11
