# Supplementary material for: Abiotic Stress Phenotypes Are Associated with Conserved Genes Derived from Transposable Elements
Source: Front Plant Sci. 2017 Nov 28;8:2027. doi: 10.3389/fpls.2017.02027 (PMC5715367; doi:10.3389/fpls.2017.02027)
Supplement: Supplementary file 5 [file Image2.PDF]

A

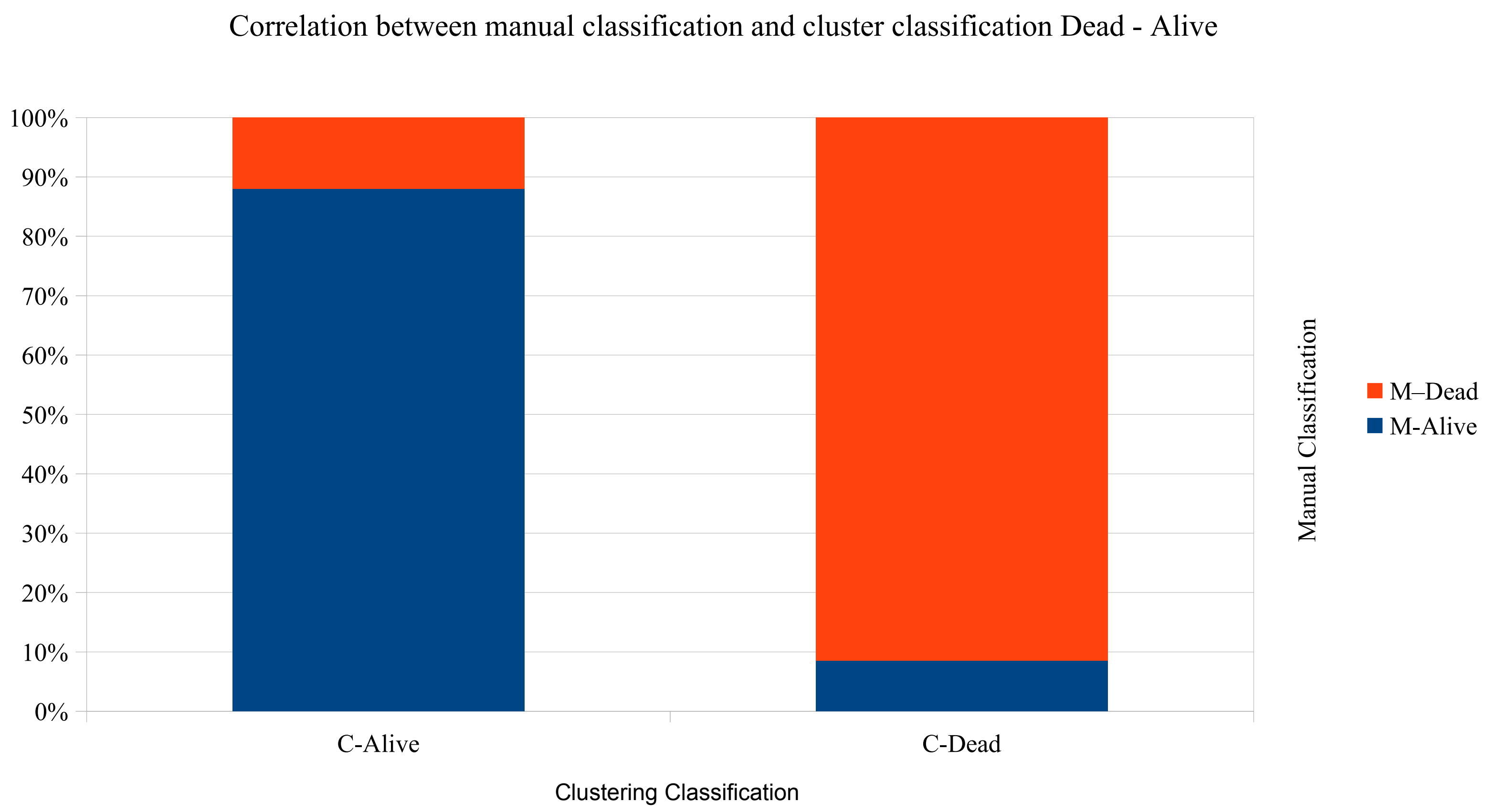
Correlation between manual classification and cluster classification Dead - Alive
100%
90%
80%
70%
60%
50%
40%
30%
20%
10%
0%
C-Alive
C-Dead
Clustering Classification
Manual Classification
M–Dead
M-Alive

B

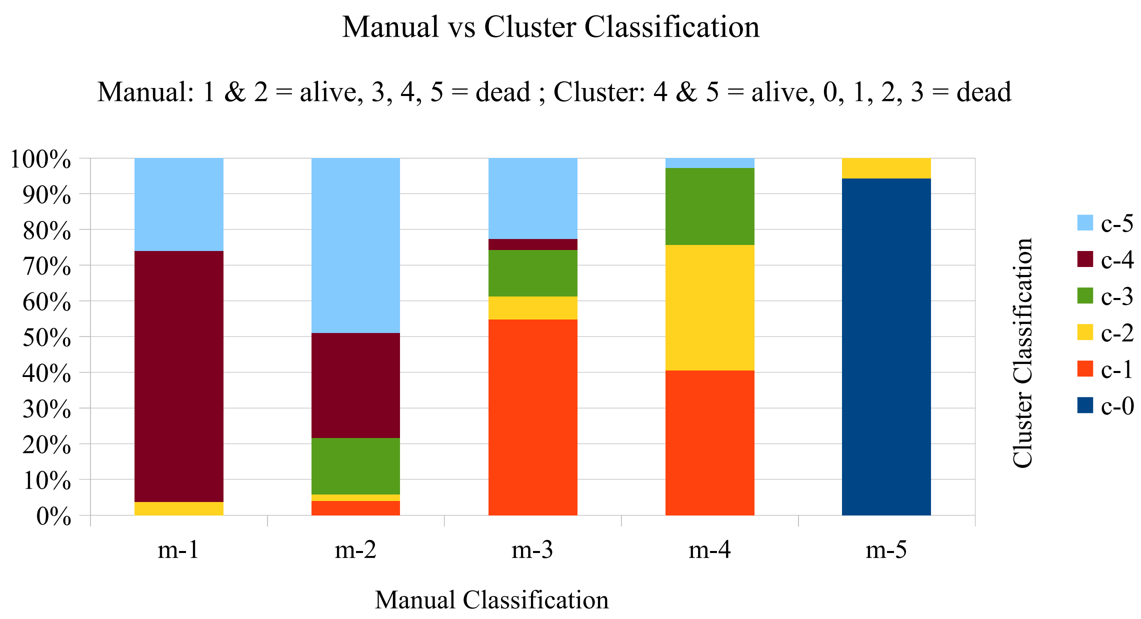
Manual vs Cluster Classification
Manual: 1 & 2 = alive, 3, 4, 5 = dead ; Cluster: 4 & 5 = alive, 0, 1, 2, 3 = dead
100%
90%
80%
70%
60%
50%
40%
30%
20%
10%
0%
m-1
m-2
m-3
m-4
m-5
Manual Classification
Cluster Classification
c-5
c-4
c-3
c-2
c-1
c-0

Supplementary Figure S2. Validation of the cluster classification using manual classification for the high-salt stress assay. The high-salt stress assay was conducted as described in the Materials and Methods. Following the image capture of the plates, a manual scoring was performed , where each seedling was given a numerical ID and a score of alive or dead was recorded. The same plate images were also used for the cluster classification analysis and the categories were attributed (c0-c5) based on the manual scoring of dead/alive. A) Stacked histogram showing the proportion of seedlings defined as alive and dead according to the cluster classification and the percentage of these seedlings that were scored as dead/alive (see legend). n = 216 seedlings. B) Stacked histogram showing the different cluster classification used (c0-c5) to determine which cluster classes reflects the manual scoring of alive/dead. m= manual; c=cluster.
