# Supplementary material for: Abiotic Stress Phenotypes Are Associated with Conserved Genes Derived from Transposable Elements
Source: Front Plant Sci. 2017 Nov 28;8:2027. doi: 10.3389/fpls.2017.02027 (PMC5715367; doi:10.3389/fpls.2017.02027)

Figure S6. Phenotypic profile for all T-DNA mutant lines and wild-type under arsenic stress conditions. Radar plots for the selected morpho-colorimetric traits: area, perimeter, circularity, compactness, major axis, minor axis, eccentricity, and grey intensity peak (hisgreypeak) under the arsenic toxicity assay. Each plot represents a phenotypic profile of particular mutant line including wild-type. The selected traits are assigned to a portion of the circle and the radius size is the independent calculated scaled value. The lines are identified with the same ID as in Table S2. Colors represent the traits and the assignment key is placed in the right bottom corner.

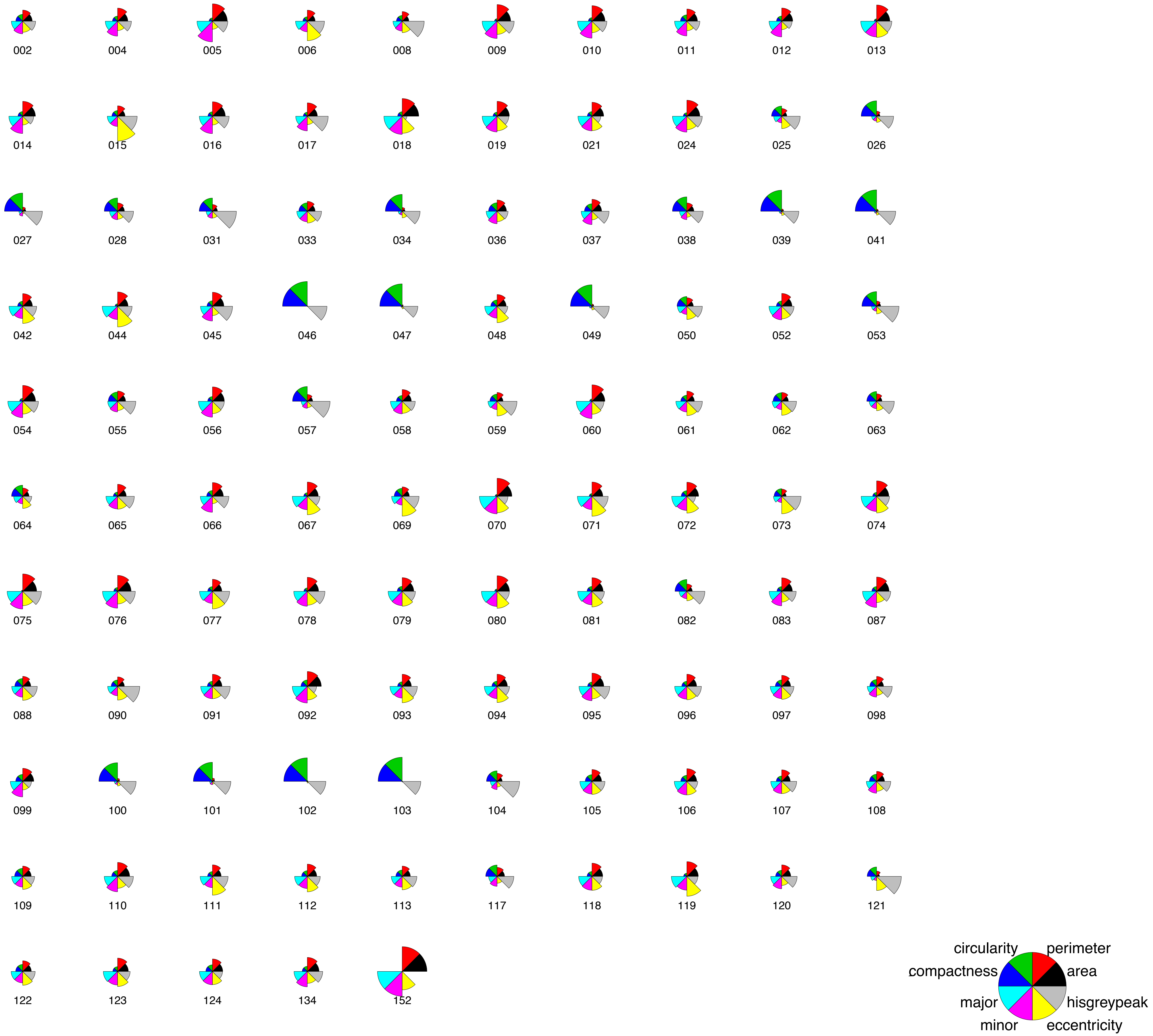

Supplement: Supplementary file 9 [file Image6.PDF]
